# From a drug repositioning to a structure-based drug design approach to tackle acute lymphoblastic leukemia

**DOI:** 10.1038/s41467-023-38668-2

**Published:** 2023-05-29

**Authors:** Magali Saez-Ayala, Laurent Hoffer, Sébastien Abel, Khaoula Ben Yaala, Benoit Sicard, Guillaume P. Andrieu, Mehdi Latiri, Emma K. Davison, Marco A. Ciufolini, Paul Brémond, Etienne Rebuffet, Philippe Roche, Carine Derviaux, Edwige Voisset, Camille Montersino, Remy Castellano, Yves Collette, Vahid Asnafi, Stéphane Betzi, Patrice Dubreuil, Sébastien Combes, Xavier Morelli

**Affiliations:** 1 Centre de Recherche en Cancérologie de Marseille (CRCM), CNRS, INSERM, Aix-Marseille Univ, Institut Paoli-Calmettes, Marseille, France; 2 Institut Necker Enfants Malades (INEM), INSERM, Hôpital Necker Enfants-Malades, Laboratory of Onco-Hematology, Assistance Publique-Hôpitaux de Paris, Université de Paris, Paris, France; 3 Department of Chemistry, Faculty of Science, University of British Columbia, Vancouver, BC Canada; 4 Present Address: Drug Discovery Program, Ontario Institute for Cancer Research (OICR), Toronto, ON Canada; 5 Present Address: Department of Chemistry, Simon Fraser University, Burnaby, BC Canada

**Keywords:** Structure-based drug design, Cheminformatics, Drug discovery and development, Drug development, Acute lymphocytic leukaemia

## Abstract

Cancer cells utilize the main de novo pathway and the alternative salvage pathway for deoxyribonucleotide biosynthesis to achieve adequate nucleotide pools. Deoxycytidine kinase is the rate-limiting enzyme of the salvage pathway and it has recently emerged as a target for anti-proliferative therapies for cancers where it is essential. Here, we present the development of a potent inhibitor applying an iterative multidisciplinary approach, which relies on computational design coupled with experimental evaluations. This strategy allows an acceleration of the hit-to-lead process by gradually implementing key chemical modifications to increase affinity and activity. Our lead compound, OR0642, is more than 1000 times more potent than its initial parent compound, masitinib, previously identified from a drug repositioning approach. OR0642 in combination with a physiological inhibitor of the de novo pathway doubled the survival rate in a human T-cell acute lymphoblastic leukemia patient-derived xenograft mouse model, demonstrating the proof-of-concept of this drug design strategy.

## Abstract (web summary)

Deoxycytidine kinase is the rate-limiting enzyme of the salvage pathway and it has recently emerged as a target for antiproliferative therapies for cancers where it is essential. Here, the authors develop a potent inhibitor applying an iterative multidisciplinary approach, which relies on computational design coupled with experimental evaluations.

## Introduction

DNA synthesis and repair from deoxyribonucleotide triphosphates (dNTPs) are enhanced in cancer; however, cellular dNTP levels are low and only support a few minutes of DNA replication^1–3^. Therefore, dNTP pools are produced “on demand” by redundantly and strongly regulated biosynthetic pathways^4–6^. There are two types of deoxyribonucleotide pathways: (i) the de novo pathway (DNP), where nucleotides are assembled from simpler compounds, and (ii) the salvage pathway (SP), where degraded RNA and DNA are recycled into new nucleotides. The DNP uses glucose and amino acids as carbon and nitrogen sources to produce ribonucleotide diphosphates, which are converted into deoxyribonucleotide diphosphates by the key enzyme ribonucleotide reductase (RNR)^7^. Therapies to block this pathway, such as RNR inhibitors, have been developed in recent decades^8,9^; however, tumor resistance could arise through the upregulation of the alternative SP. The SP recycles preformed nucleoside fragments from the degradation of RNA and DNA into nucleotides through the combined action of metabolic kinases and phosphoribosyl transferases. Nucleobases are salvaged by phosphoribosyl transferases: adenine phosphoribosyl transferase (APRT), which forms AMP from adenine, and hypoxanthine-guanine phosphoribosyl transferase (HPRT) that forms IMP (inosine monophosphate) from hypoxanthine and GMP (guanosine monophosphate) from guanine. In contrast, nucleosides are salvaged by nucleoside kinases. The first enzymatic step in the cytosolic SP is catalyzed by thymidine kinase 1 (TK1), which phosphorylates thymidine (dT). In addition, the deoxycytidine kinase (dCK) catalyzes the 5′-phosphorylation of physiological pyrimidines and purines, including 2′-deoxycytidine (dC), 2′-deoxyadenosine (dA) and 2′-deoxyguanosine (dG). dCK is the rate-limiting enzyme and the key member of the SP due to its broad substrate specificity^10^. dCK is also required for the first phosphorylation of numerous anticancer and antiviral nucleoside analogs, such as gemcitabine, cytarabine and lamivudine^11^. Then, deoxynucleoside monophosphates (dNMPs) are further phosphorylated into deoxynucleoside triphosphates (dNTPs) in two additional steps, performed by nucleoside monophosphate kinase (NMPK) and nucleoside diphosphate kinase (NDPK).

Cancer cells encounter insufficient biosynthetic capacity using the main DNP because of the increased consumption of dNTPs due to enhanced and uncontrolled proliferation. To meet the metabolic requirements for cell growth, cancer cells must engage both the DNP and SP to reach adequate nucleotide pools to support their nucleic acid synthesis rate. While it is poorly understood how the DNP and SP work in tandem, it has been established that the interrelation of these pathways enables the upregulation of the SP upon DNP inhibition, which increases the dependency of specific cancer cells on the SP for nucleotide production^12^. Among cancers, lymphoid leukemias and lymphomas have been shown to be highly sensitive to chemotherapy regimens inhibiting nucleotide synthesis. Indeed, these diseases have shown increased dCK expression relative to normal tissues, as revealed by the RNASeq and quantitative proteomics data of a large panel of human cancer models in the Cancer Cell Line Encyclopedia^13^ and the Human Protein Atlas^14^. Because dCK is the rate-limiting enzyme of the SP, it is a target of choice for antiproliferative therapies for cancers where the SP is essential or upregulated. In this context, dCK has recently been validated as a relevant target in oncology. In a study on acute lymphoblastic leukemia (ALL), a cancer for which treatment options are lacking, Nathanson et al.^12^ confirmed the ability of cancer cells to switch their dNTP synthesis from the DNP to the SP. Cotargeting both pathways for dCTP biosynthesis was well tolerated in mice and efficacious in T-cell and B-cell ALL (T-ALL and B-ALL) models, confirming the combined targeting of both the DNP and SP as an efficacious treatment of these cancer cells. Another study proposed that nucleotide biosynthetic plasticity in leukemia cells is mediated by both ataxia telangiectasia and Rad3-related protein (ATR) signaling and nucleotide metabolic adaptive mechanisms^15^. The authors hypothesized and confirmed that dCK was responsible for an important resistance mechanism to ATR inhibition in leukemia, as dCK activity could compensate for ATRi-induced downregulation of RNR. Thus, dCK is an effective target for dual DNP/SP therapeutic strategies with a synthetic lethality approach.

In a previous study^16^, we identified dCK as an off-target of the selective tyrosine kinase inhibitor (TKi) masitinib, applying a reverse-proteomics approach (drug repositioning). Masitinib is under evaluation in phase III clinical trials for several pathologies, such as mastocytosis and amyotrophic lateral sclerosis, but it can also resensitize gemcitabine-refractory cancer cell lines when used in combination with gemcitabine (a deoxycytidine analog used in chemotherapy)^17,18^. The target responsible for this sensitization is dCK, and we revealed how masitinib, as well as a few other TKis used to treat cancer, such as imatinib, interacts with dCK. We discovered this previously unknown molecular interaction, which results in the activation of dCK. This activation leads to increased phosphorylation of both physiological (e.g., dC) and prodrug (e.g., gemcitabine) substrates of this nucleotide kinase. Crystal structures of dCK in complex with masitinib and imatinib were solved and we were able to describe how dCK is activated by a conformational-dependent mechanism involving a dynamic functional pocket that partially overlaps the substrate binding site. As masitinib was shown to be an excellent modulator of dCK enzymatic activity and the resolution of the crystal structure provided key information about the binding mode, we decided to start a drug design program to develop new derivatives of masitinib, inhibitors of dCK.

Discovery and development of new drugs is a highly costly and slow process and repositioning of old drugs to treat other diseases is increasingly becoming an attractive proposition because it involves the use of compounds with potentially lower overall development costs and shorter development timelines^19^. In this context, in the interface between drug design and drug repositioning, we decided to apply an integrative multidisciplinary drug design approach to repurpose an existing drug on a new target and develop new derivatives to accelerate the process of classic drug discovery, hit identification and hit-to-lead optimization.

We hypothesized that an increased level of interaction with dCK could trigger a switch from activation to inhibition; thus, in this work, we rationally design new masitinib derivatives to increase their binding site complementarity and dCK affinity to induce enzymatic inhibition. Subsequently, it was thought that the combination of the newly designed dCK inhibitors with an RNR inhibitor could efficiently fight cancer cells and prevent escape mechanisms, as both pathways (the DNP and SP) would be simultaneously inhibited. We applied our hit-to-lead integrative approach, Diversity-oriented target-focused synthesis (DOTS), to masitinib. DOTS relies on a highly automatized process of in silico chemical library design and fragment-based drug design coupled to experimental evaluations^20^. In the present study, we design an original library of masitinib derivatives and gradually implement key chemical modifications to increase protein-ligand interactions and activity. The mode of action of these probes is explored: we study dCK affinity for new compounds, solve the 3D crystal structures of the best derivatives in complex with dCK, and evaluate the effect on dCK enzymatic activity. Moreover, we assess the in vitro antiproliferative activity of these new dCK inhibitors, in combination with an RNR inhibitor, in a T-ALL cellular model. Finally, the leading dCK inhibitor OR0642 is studied in vivo in combination with a physiological inhibitor of RNR. The combined therapy doubles the median survival in a human leukemia mouse model as well as in a patient-derived xenograft model, which exhibits the therapeutic potential (at least) for T-ALL treatment. The present work demonstrates the proof-of-concept of this drug design approach, where the repurposing of a drug (masitinib, a TKi-targeting c-KIT) on a new target (dCK) can be used as a starting point for the automated process of design and accelerated optimization of better drugs against cancer.

## Results

### From activation to inhibition: the proof of concept

**Fig. 1:**
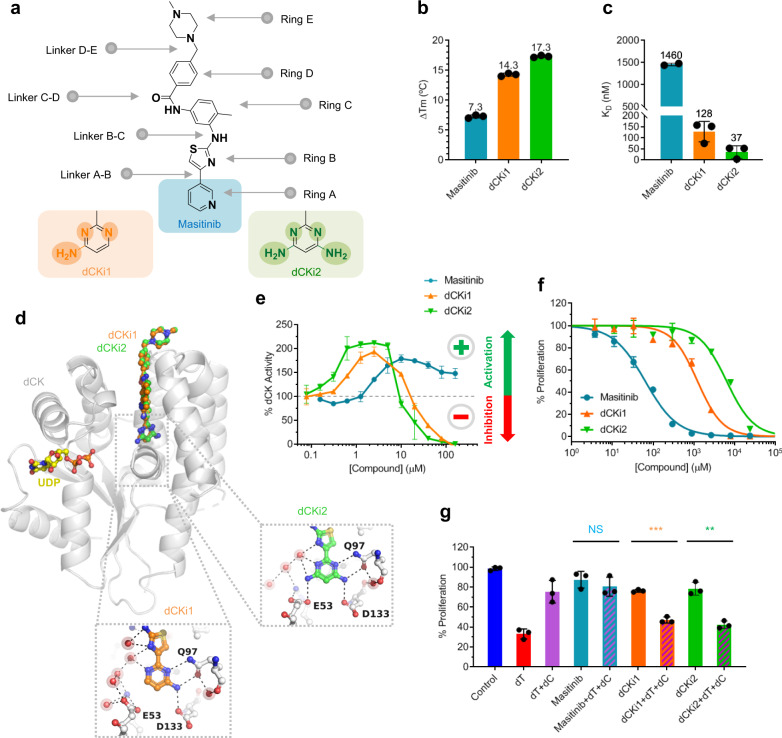
The two first masitinib derivatives show increased affinity to dCK and enzymatic inhibition. **a** Masitinib, dCKi1 and dCKi2 structures showing rings and linkers. **b** dCK thermal stabilization by masitinib, dCKi1 and dCKi2 measured by TSA (*n* = 3). **c** dCK binding to masitinib^16^, dCKi1 and dCKi2 determined by ITC (*n* = 3). **d** Superimposition of crystal structures of dCK (gray ribbons) in complex with UDP (yellow) and dCKi1 (orange) (PDB 7ZI1) or dCKi2 (green) (PDB 7ZI2). **e** Representative experiment showing the effect of masitinib, dCKi1 and dCKi2 on substrate phosphorylation by dCK in the presence of UTP (*n* = 3). **f** Representative experiment showing the effect of masitinib, dCKi1 and dCKi2 on cell proliferation of the IL3 dependent Ba/F3 cell line (expressing WT human c-KIT) in the absence of IL3 and presence of the Cell Stem Factor (SCF) (proliferation depending on c-KIT) (48 h) (*n* = 3). **g** Effect of masitinib, dCKi1 and dCKi2 (10 µM) on cell proliferation of the leukemia cell line CCRF-CEM (T-ALL) in the absence or presence of dT (111 µM) and dC (1 µM) (72 h). NS represents no statistical significance between masitinib and masitinib+dT + dC. Statistical significance is **P = 0.0011 and ***P = 0.0001 for dCKi1 and dCKi2, respectively (dCKi + dT + dC compared to dCKi alone, two-tailed unpaired t-test). All data are presented as the mean ± SD (*n* = 3).

**Fig. 2:**
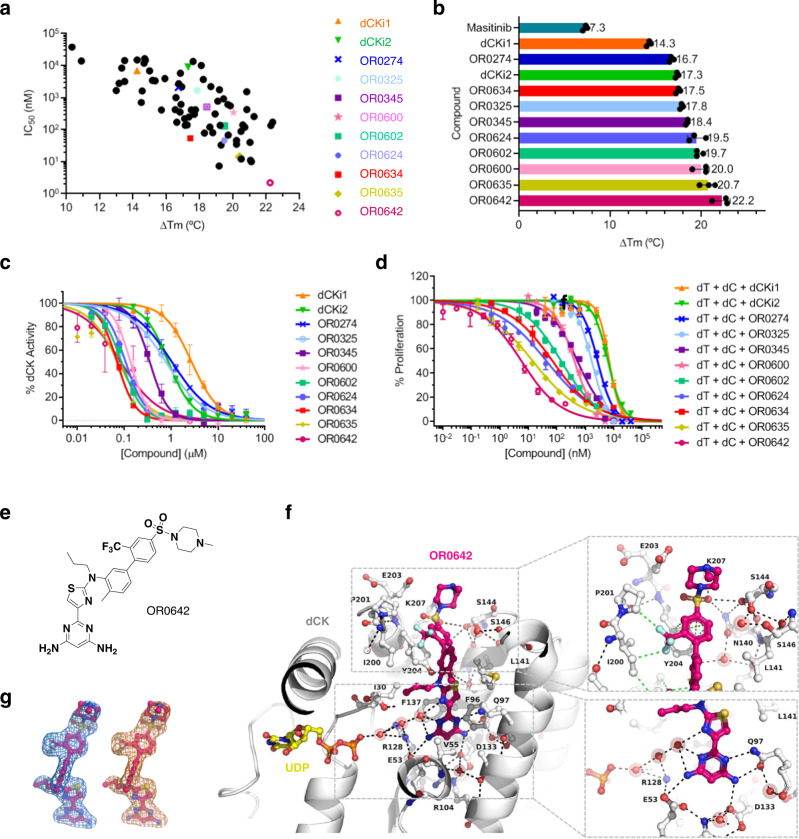
Optimization of dCK inhibitors with improved affinity, enzymatic inhibition and cellular activity. **a** Effect on dCK thermal stabilization (determined by TSA) and dCK cell proliferation assay (CCRF-CEM cell line) produced by the 74 synthetized compounds. Data are presented as the mean. **b** dCK thermal stabilization by selected compounds measured by TSA. Data are presented as the mean ± SD (*n* = 3). **c** Representative curves of one of two independent experiments showing the effect of selected compounds on substrate phosphorylation by dCK in the presence of ATP. Data are presented as the mean of three technical replicates ± SD. **d** Representative experiment showing the effect of selected compounds on cell proliferation of the CCRF-CEM cell line in the presence of dT (200 µM) and dC (1 µM) Data are presented as the mean ± SD (*n* = 3). **e** Chemical structure of OR0642. **f** Structure of dCK (gray ribbon) in complex with OR0642 (magenta) and UDP (yellow) (PDB 7ZI3) showing the hydrogen bond network to dCK (black dashed lines). VdW interactions between OR0642 CF_3_ group and dCK ILE-200 and PRO-201 are shown as green dashed lines in the zoom insert. **g** The meshes around the compound shows the |2Fo|−|Fc| electron density map contoured at 2.0σ around the ligand (blue) and the |Fo|−|Fc| electron density map (orange) contoured at 1.0σ generated with the model compound omitted before refinement (omit map).

The starting point for this work was the exploitation of the crystal structure interactions observed between dCK and masitinib, located in an extension of the enzymatic active pocket that is only accessible in its open form^16^. Surprisingly, this complex was mainly driven by hydrophobic contacts and motivated a structure-activity relationship (SAR) program aimed at improving ligand/dCK complementarity to develop potent dCK inhibitors. As a proof of concept, we first rationally designed and synthesized two masitinib derivatives, named “dCKi1” and “dCKi2” (Fig. 1a), with minor modifications on the pyridine ring (ring A). This ring partially overlaps with the substrate binding site, and we decided to mimic contacts with the surrounding residues, as seen for the 4-aminopyrimid-2-one of the physiological substrate deoxycytidine (dC) when bound in the substrate cavity (Supplementary Fig. 1). The binding of these two analogs to dCK was assessed using a thermal shift assay (TSA) (Fig. 1b) and isothermal titration calorimetry (ITC) (Fig. 1c and Supplementary Fig. 2). As expected, dCKi1 and dCKi2 displayed an increased binding affinity to dCK. The dCK thermal stabilization produced by these new compounds was greatly increased from 7.4 °C for masitinib to 14.3 and 17.3 °C for dCKi1 and dCKi2, respectively. The 1.4 μM dissociation constant (*K*_D_) observed for masitinib, as determined by ITC^16^, was improved more than tenfold following these two minor chemical modifications; we measured *K*_D_ values of 128 nM for dCKi1 and 37 nM for dCKi2. The crystal structures of dCK in complex with dCKi1/uridine diphosphate (UDP) or dCKi2/UDP confirmed a similar binding mode to masitinib. dCKi1 and dCKi2 extend from the substrate binding site to the solvent occupying the allosteric dynamic functional pocket already described for previous dCK inhibitors^21–23^, far away from the phosphate donor site occupied by UDP (see Fig. 1d, Supplementary Fig. 1, and crystallization methods). The structures also revealed additional hydrogen bond contacts between dCK and the new compounds (Fig. 1d), explaining the increased affinity observed previously by the TSA and ITC. The key residues involved in these interactions are GLU-53, GLN-97 and ASP-133, three amino acids located at the substrate binding site. Moreover, biochemical assays were performed to study the enzymatic phosphorylation of dC by dCK in the presence of UTP and dCKis (Fig. 1e) (see enzymatic assay methods for further information about the phosphate donor). These experiments revealed a shift in dCK enzymatic activity. While masitinib is a modulator that only increases dCK enzymatic activity, its derivatives also induce a decrease in dCK enzymatic activity at high concentrations; therefore, dCKi1 and dCKi2 are shifted from activators to inhibitors (in the micromolar range). Furthermore, assays in cells expressing human c-KIT receptor (the main tyrosine kinase targeted by masitinib) were performed and confirmed the decrease in the inhibitory effect on this target following these two minor chemical modifications (Fig. 1f). Finally, we developed a differential metabolic cellular assay to study inhibitors of the DNP and SP and validated whether synergistic inhibition of these pathways by the combination of a dCK inhibitor with an RNR inhibitor could lead to the inhibition of cellular proliferation. We used the physiological RNR inhibitor thymidine (dT), since the dTTP produced via thymidine kinase from exogenously added dT acts as an RNR inhibitor for pyrimidine reduction by allosteric regulation of the R1 subunit^24^. The addition of dC completely prevented dT-induced S-phase cell cycle arrest and restored proliferation by the SP via dCK. The synergistic relationship between the DNP and SP and the impact of exogenously added dT, dC and dCKi on cell cycle arrest are summarized in Supplementary Fig. 3. The results on the human leukemia (T-ALL) cell line CCRF-CEM revealed that while masitinib was unable to inhibit cancer cell proliferation in combination with dT (Fig. 1g), dCKi1 and dCKi2 inhibited their proliferation at a low micromolar concentration (IC_50_ 6.9 and 8.9 µM, respectively) (Fig. 2d).

### Integrated (iterative) approach for hit-to-lead optimization

The successful transition from activator to inhibitor induced by these chemical modifications motivated the development of a ‘hit-to-lead’ optimization strategy with dCKi1/dCKi2 as the starting point. We previously developed a heavily automated iterative and multidisciplinary fragment-based drug design (FBDD) approach, relying on computational design coupled with experimental evaluation via DOTS^20^. This method was designed to handle both growing and linking strategies commonly used in the context of FBDD. Briefly, in silico stages of DOTS originally relied on two main steps: (i) design of a diversity-oriented chemical library by combining an activated form of the initial hit with a collection of available building blocks using compatible medicinal chemistry reactions and (ii) virtual screening of this library using a template-based docking method to prioritize the best interacting compounds. DOTS has previously been validated by our team on other medicinal chemistry programs and successfully generated potential lead compounds in a reduced time frame^20^.

The goal here was to extensively explore the chemical space around dCKi1 and dCKi2 compounds to optimize the series while minimizing the number of synthesized and screened compounds:^25,26^ an ‘integrative multidisciplinary drug design’ accelerated program. However, in contrast to previous growing-based fragment-to-lead projects, multiple chemical elements of the compounds were investigated at the same time. Thus, it was more efficient to perform a systematic exploration of each moiety by plugging in a dictionary of commonly used organic modifications (functional groups, rings with different spacers) than relying on the original DOTS workflow.

Finally, the designed compounds were directly evaluated in SeeSAR software, using the template-based docking mode, to quickly identify the most promising chemical modifications. Besides, additional designs, such as the chimeric compounds between different series, were also manually evaluated within SeeSAR. Finally, a visual analysis of the binding modes, the lack of conformation warnings and common physicochemical properties (e.g., logP or MW) were used to prioritize compounds to be synthesized.

Following the synthesis of each new analog series, the experimental multidisciplinary pipeline was implemented to evaluate each compound using methods from biophysics, biochemistry, structural biology and cellular biology. The dCK affinity of the newly synthesized compounds was determined by the TSA, while the enzymatic activity of dCK was assessed in the presence of UTP and ATP when necessary to determine the modulatory effect of compounds (activator versus inhibitor). Crystal structures were solved, and a model cell line (CCRF-CEM) was used to study the inhibition of cellular proliferation. In this iterative workflow, knowledge obtained in one step is directly engaged for the optimization of a new series of compounds.

### Compound optimization studies by the TSA, enzymatic assay, X-ray crystallography and cellular assay

**Fig. 3:**
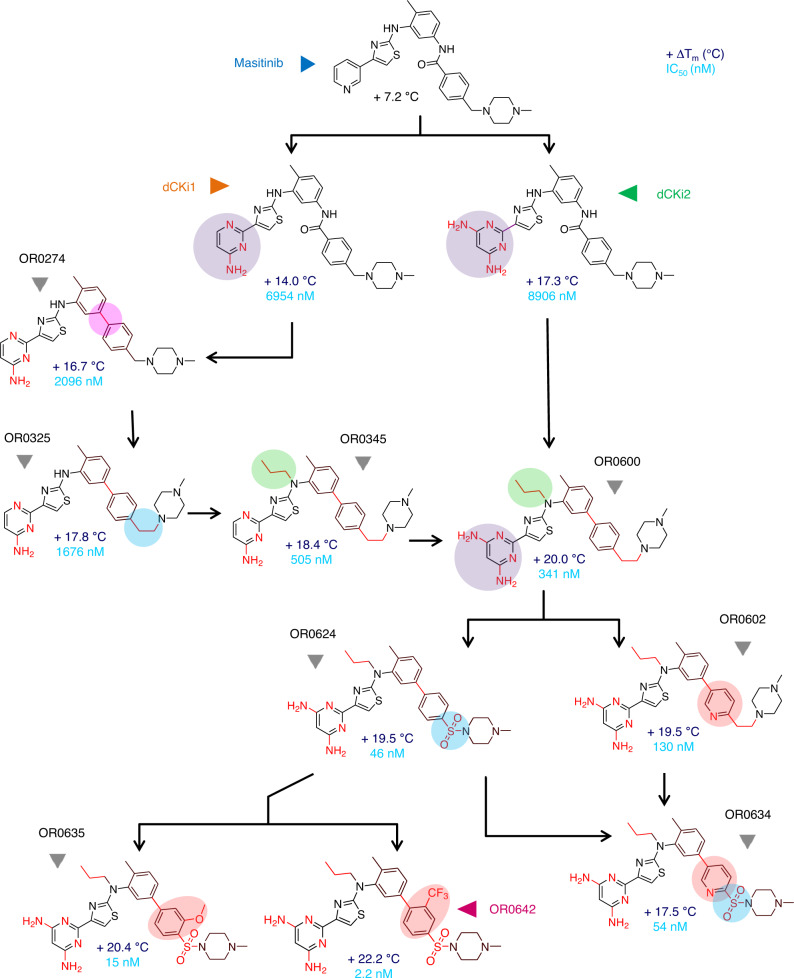
Hit-to-lead decision tree. Decision tree of the hit-to-lead optimization campaign showing the 9 key milestone compounds that drove the SAR optimization from masitinib (blue), dCKi1 (orange) and dCKi2 (green) to ORO0642 (magenta). For each compound the TSA (Δ*T*_m_, °C) and dCK cellular (IC_50_, nM) measured values are indicated. At each step, the modified portion of the molecule is highlighted by a colored disk and the overall modifications with regard to masitinib are highlighted in red.

The 5 masitinib rings (A, B, C, D and E) and 3 of the 4 linkers (Fig. 1a) as well as substructure fusions were sequentially explored during this DOTS/medicinal chemistry campaign. Through several cycles of optimization, more than 1000 analog compounds were designed and evaluated within the 3D structure of the target. The top ranked compounds of each cycle were selected for synthesis and evaluation, finally leading to a total of 74 compounds. The experimental evaluations by the TSA and cellular assay obtained for these 74 compounds are summarized in Supplementary Table 1 and Fig. 2a. The dCK thermal stabilization produced by the binding of these compounds ranged from +4.1 to +22.4 °C (Δ*T*_m_). Using the dCK cellular assay (Supplementary Fig. 3), we studied whether the inhibition of both DNP and SP by the combination of dT with the new dCK inhibitors could lead to the inhibition of cellular proliferation. The results on the human leukemia (T-ALL) cell line CCRF-CEM revealed that new compounds were able to inhibit cancer cell proliferation in combination with dT. The obtained IC_50_ cell proliferation values ranged between 37 µM and 2 nM. From all these synthesized derivatives, 9 compounds permit illustration of the whole optimization process toward the final lead compound. These compounds exhibit key modifications that drive lead optimization forward by increasing biochemical properties, affinity and in vitro activity (Fig. 2b–d). The medicinal chemistry campaign is summarized in Fig. 3, in which these 9 key compounds of the SAR studies, as well as dCKi1 and dCKi2, are placed as milestones of the optimization in a decision tree that led to our lead compound.

After the first modification of cycle A in dCKi1, the linker C–D was studied, as the amide group was predicted to be suboptimal in dCK and is also known as a key element of the TKi pharmacophore. Diverse replacement linkers (e.g., reverse amide, ether, alkane/alkene/alkyne chains of various lengths, and additional rings) were evaluated in silico, and the suppression of the amide moiety to obtain a linear biphenyl core (OR0274) was prioritized as the most advantageous modification, increasing affinity (Δ*T*_m_ + 16.7 °C) and in vitro cellular activity (IC_50_ 2096 nM). The linker D–E was elongated by one carbon (OR0325) to compensate for the loss of the amide group and better accommodate ring E within the binding site. As a consequence, both affinity and in vitro cellular activity were increased (Δ*T*_m_ + 17.8 °C and IC_50_ 1676 nM). It was also predicted that adding a hydrophobic chain on the nitrogen atom from linker B-C could have a favorable impact on affinity by making strong van der Waals (vdW) contacts with several close hydrophobic residues. In an attempt to optimize this linker, we synthesized a series of analogs, and the *N*-propyl derivative (OR0345) showed a very interesting profile, with total loss of dCK enzymatic activation (Supplementary Fig. 4), increased affinity and a 3-fold decrease in cellular IC_50_ (Δ*T*_m_ + 18.4 °C and IC_50_ 505 nM). The X-ray structure confirmed our hypothesis with optimal vdW contacts between the propyl moiety and several hydrophobic residues (ILE-30 and ILE-200) (Supplementary Fig. 5).

Later, a fusion analog consisting of several successful modifications was synthesized (OR0600) by merging the slightly improved dCKi2 with the dCKi1 optimization conclusions. As expected, this derivative exhibited increased affinity, reaching a dCK stabilization of +20 °C (Δ*T*_m_). Moreover, this compound only exhibited enzymatic inhibition (without activation at low concentrations) due to the presence of the *N*-propyl moiety (Supplementary Fig. 4) and improved in vitro cellular activity (IC_50_ 341 nM). From this potent compound, ring D was then explored, and the most interesting compound from this series consisted of a pyridine in place of the phenyl ring in OR0602, for which affinity was maintained, while solubility and enzymatic inhibition were enhanced, reaching the technical limit of enzymatic assay (IC_50_ 77 nM). Moreover, cellular activity was improved by a factor of ~2.4 (IC_50_ 130 nM) without an increase in nonspecific cellular toxicity. In parallel, linker D–E was also widely studied, and the distances and angles were optimized by the introduction of a sulfone moiety resulting in a sulfonamide (OR0624). While dCK thermal stability was similar, cellular in vitro activity was improved more than sevenfold to an observed IC_50_ of 46 nM. Due to these promising results, we decided to merge the structural modifications of OR0602 and OR0624 to obtain the hybrid compound OR0634. This compound increased molecular contacts with dCK, as determined by X-ray crystallography (Supplementary Fig. 6), maintained excellent cellular activity (IC_50_ 54 nM) and revealed better in vivo tolerance (OR0634 was well tolerated by mice even at 80 mg/kg/day, while OR0602 presented unacceptable side effects from 20 mg/kg/day (Supplementary Table 2)). Finally, a large series of modifications of rings C, D and E were undertaken to improve the activity and in vivo stability. Analogs with substitutions in ring D (OR0635 with OMe and OR0642 with CF_3_) provided the best results. For OR0635 and OR0642, dCK stabilization was improved to +20.4 °C and +22.2 °C Δ*T*_m_, respectively, and cellular in vitro activity was enhanced to IC_50_ values of 15 and 2.2 nM, respectively. The in vitro and in vivo stability of the compounds was largely improved, exhibiting half-lives longer than 5 and 7 h, respectively (Supplementary Table 2). OR0642 was well tolerated in mouse models at 80 mg/kg twice/day. During the whole optimization process, our structural data (Supplementary Figs. 5 and 6) confirmed the expected binding modes of the ligands. The X-ray structure of OR0642 (Fig. 2f, g and Supplementary Fig. 7) displays improved contact with dCK following the optimization for our lead compound; (i) the hydrogen bond network engaged in the active site with ring A, (ii) the vdW interactions generated by the CF_3_ moiety with the amino acids ILE-200 and PRO-201 and (iii) the increased contacts between the sulfonamide/ring E moiety and the amino acids ASN-140 and SER-144. Moreover, to study the selectivity of dCK toward c-KIT (the main tyrosine kinase targeted by masitinib), we performed assays in cells expressing the human c-KIT receptor. We confirmed a decrease in the inhibitory effect in c-KIT, showing an IC_50_ ratio of IL3/SCF (unspecific/c-KIT) lower than 1 for all 9 of the key compounds in the SAR studies, while the IC_50_ ratio was more than 100 for masitinib and 7.6 and 2.7 for dCKi1 and dCKi2, respectively (Supplementary Fig. 8). Furthermore, a kinome-wide profiling of OR0642 was performed and none of the 97 kinases tested was inhibited (Supplementary Table 3), which was confirmed by the absence of nonspecific toxicity in a cell proliferation assay (Supplementary Fig. 9). Finally, we generated a CCRF-CEM CRISPR-Cas9 cell line deficient for dCK and performed experiments to study the sensitivity to dT, dC and OR0642 (Supplementary Fig. 10). In the control cell line, dC rescued cell proliferation after dT effect via dCK activity and OR0642 was able to revert this effect, inducing a diminution of cell proliferation. On the contrary, in the dCK^−^ cell line, dC was unable to rescue cell proliferation due to the absence of dCK and the addition of OR0642 did not affect cell proliferation. Consequently, the non-toxic effect of OR0642 and its activity, only observed in presence of dCK, demonstrated that the biologic activity of OR0642 is mediated by dCK and not by off-targets.

Overall, the DOTS multidisciplinary strategy used for SAR optimization was validated. The optimized compound OR0642 exhibits +15 °C of improved dCK thermal stability compared to the parent compound masitinib and +7.9 °C compared to the starting point (dCKi1) of our medicinal chemistry campaign. Our lead compound OR0642 is dCK-selective and also exhibits good physicochemical properties, as highlighted by its ability to cross cellular membranes and more than 1000 times increased potency in the cell proliferation assay when compared with the first dCK inhibitor hit (dCKi1).

Lastly, we synthesized a negative control compound closely related to OR0642 (Supplementary Fig. 11). The OR0659 compound presents the same structure as OR0642, but the diaminopyrimidine (ring A) was replaced by a pyridine (as in the parent compound masitinib) and the CF_3_ substituent in the ring D was removed. As expected, this compound displayed lower binding affinity to dCK (+6.3 °C), lower enzymatic inhibition (IC_50_ > 100 µM), and was unable to inhibit cellular proliferation in the presence of dT + dC, showing the loss of effect on dCK after these two key modifications.

### Nucleotide insufficiency promotes DNA damage and cell death

To explore the mode of action of the combined therapy, we studied cell cycle progression using flow cytometry in CCFR-CEM cells exposed 24 h to OR0642 in the absence or presence of the RNR inhibitor dT and the physiological nucleoside dC (Supplementary Fig. 12). As expected, in the presence of dC, both dT and OR0642 presented a similar cell cycle profile to the DMSO control. However, the combined therapy (dT+OR0642) showed a S-phase arrest due to nucleotide insufficiency after DNP and SP inhibition. Moreover, two previously reported dCK inhibitors (DI-39 and DI-87) were also studied in the same combination conditions and results presented similar cell cycle profiles as OR0642.

We also performed experiments to study nucleotide pools to verify if nucleotide insufficiency to maintain DNA synthesis was at the origin of the biologic effect of the combined treatment (Supplementary Fig. 13). As mentioned previously, dT is converted to dTTP by TK, NMPK and NDPK via the SP. dTTP binds to the allosteric specificity site on RRM1 to favor purines reduction over the pyrimidine CDP reduction, thereby resulting in dCTP insufficiency. As expected, dT addition increased the dTTP pool (Supplementary Fig. 13a) and RNR inhibition by dTTP increased the size of the CDP pool (Supplementary Fig. 13b) and decreased the dCDP pool (Supplementary Fig. 13c), as RNR is not able to reduce CDP into dCDP. This effect was reverted by the addition of dC to mimic physiological conditions, and CDP and dCDP pools were similar to the control (Supplementary Fig. 13b, c). In this condition (dT+dC), dCK was able to phosphorylate dC and the pool of dCTP was restored by dCK via the SP (Supplementary Fig. 13d), even if dT blocked the dCTP production by the DNP via the RNR inhibition. As predicted, the addition of OR0642 alone and the subsequent dCK inhibition did not affect the nucleotide pools (Supplementary Fig. 13a–d), because under basal conditions, DNA synthesis relies primarily on the DNP-produced dCTP^27^. In contrast, the combination therapy dT+OR0642 in presence of dC (dT + dC + OR0642) was able to decrease the dCTP pool (Supplementary Fig. 13d), as both pathways are inhibited (DNP by dT and SP by OR0642), even in presence of dC at physiological concentration. These results confirm our hypothesis and are in complete agreement with previous reports where RNR inhibition triggered a compensatory upregulation of the salvage pathway increasing salvage dCTP biosynthesis, which can be blocked by a dCK inhibitor^12,15^.

Further studies determined that neither dT nor OR0642 alone induced DNA damage or apoptosis in cells in the presence of dC (Supplementary Figs. 14 and 15). In contrast, the combined therapy dT+OR0642 triggered induction of DNA damage, as evidenced by the presence of the histone variant H2A.X phosphorylated on its serine 139 (γH2A.X) determined by flow cytometry (Supplementary Fig. 14a, b) and immunofluorescence microscopy (Supplementary Fig. 14c). Moreover, dT+OR0642 also induced apoptosis, as measured by Annexin V staining by flow cytometry (Supplementary Fig. 15). All these results were compared to the effect induced by other previously described dCK inhibitors (DI-39 and DI-87). Data of combined therapies (dT + dCK inhibitors) were similar with the three compounds (OR0642, DI-39 and DI-87), showing the same mechanism of cell death published before, in which persistent nucleotide insufficiency triggers replication stress that promotes lethal DNA double-stranded breaks.

### Successful synthetic lethality in a T-ALL mouse model

To investigate whether the efficacy observed in vitro could also be observed in vivo, our lead compound OR0642 obtained after SAR optimization was used in a systemic leukemia in vivo mouse model. We studied the in vivo efficacy and tolerability of cotargeting the alternative nucleotide biosynthetic DNP and SP in a NOD-SCID-IL2Rγc null (NSG) mouse model inoculated intravenously with the human CCRF-CEM T-ALL cell line expressing luciferase. The DNP was inhibited with dT, the physiological inhibitor of RNR, and the SP was inhibited with the newly developed dCK inhibitor OR0642.

**Fig. 4:**
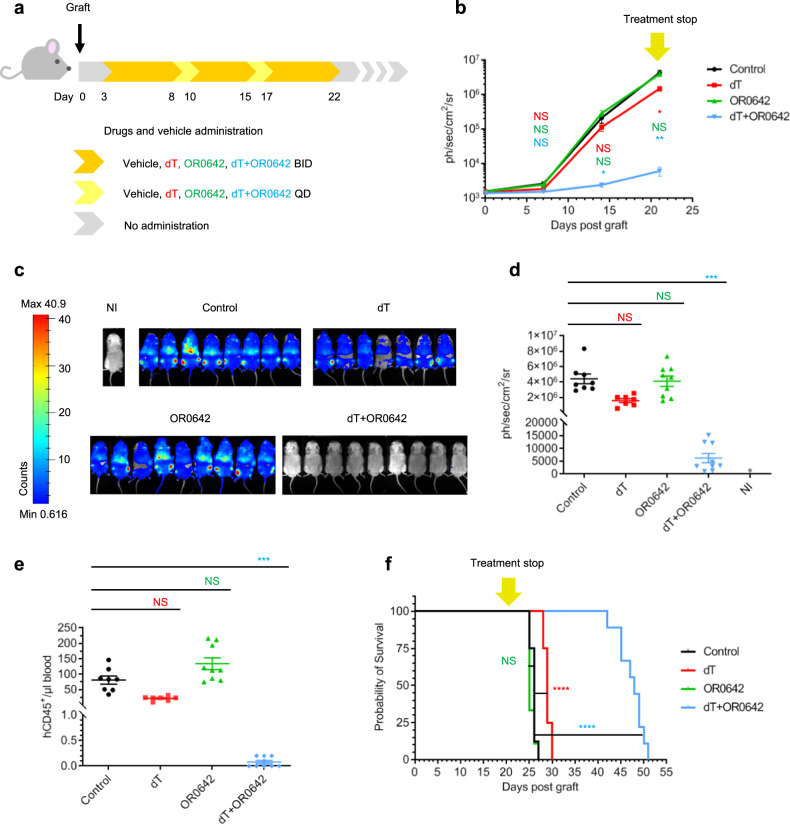
OR0642 in combination with dT has strong antileukemic effect in vivo. **a** Schedule of the vehicle and drugs administration mice groups (vehicle (*n* = 8), dT 1.5 g/kg (*n* = 7), OR0642 40 mg/kg (*n* = 9) and dT 1.5 g/kg + OR0642 40 mg/kg (*n* = 9)) (once/day (QD) and twice/day (BID)) against CCRF-CEM leukemia bearing mice. **b** Effect of treatments on the quantification of whole body radiance of CCRF-CEM leukemia bearing mice (bioluminescence). The yellow arrow highlights the end of the treatment. Data are presented as the mean ± SEM and compared using two-way ANOVA test and Turkey’s multiple comparison test (vehicle (*n* = 8), dT (*n* = 7), OR0642 (*n* = 9) and dT + OR0642 (*n* = 9)). NS represents no statistical significance, *P < 0.05, and **P = 0.001. **c** Representative bioluminescence images of mice at day 21 (NI = non injected mouse; without graft) (*n* = 3). **d** Effect of treatments on the quantification of whole body radiance at day 21. (NI = non injected mouse; without graft). Data are presented as the mean ± SEM. Comparison of bioluminescence data at day 21 was performed using one-way ANOVA Kruskal–Wallis test and Dunn’s multiple comparison test (vehicle (*n* = 8), dT (*n* = 7), OR0642 (*n* = 9) and dT + OR0642 (*n* = 9)). NS represents no statistical significance and ***P = 0.0002. **e** Effect of treatments on hCD45^+^ populations determined by flux cytometry at day 21. Data are presented as the mean ± SEM. Comparison of hCD45^+^ populations was performed using one-way ANOVA Kruskal–Wallis test and Dunn’s multiple comparison test (vehicle (*n* = 8), dT (*n* = 7), OR0642 (*n* = 9) and dT + OR0642 (*n* = 9)). NS represents no statistical significance and ***P = 0.0005. **f** Survival analysis of CCRF-CEM leukemia bearing mice treated with vehicle and drugs. The yellow arrow highlights the end of the treatment. Median survival times were compared using Log-Rank (Mantel–Cox) Test. NS represents no statistical significance and ****P < 0.0001.

Treatment was initiated on day 3 after grafting and was maintained for 19 days with cycles of 5 days (BID, twice/day) and 2 days (QD, once/day) (Fig. 4a). We assessed the efficacy of the combination therapy in comparison with the administration of the vehicle (control) and the administration of each of the two components individually. Leukemic progression was monitored by bioluminescent imaging once a week (Fig. 4b). On day 21, mice in the combination treatment (dT + OR0642) had a more than 100-fold lower bioluminescent signal than control mice or mice treated with either of the compounds individually, indicating a significant decrease in systemic leukemic burden and promising therapeutic efficacy (Fig. 4c, d). Moreover, hCD45^+^ populations in blood samples were measured by flux cytometry on day 21, and combination therapy significantly decreased disease progression (Fig. 4e). As expected, according to the synthetic lethality concept, the two components when administered separately were poorly efficacious in vivo*;* OR0642 did not show any antiproliferative effect, and dT scarcely reduced leukemic progression (Fig. 4b). As a consequence, all the control and single-treated mice died after 25–30 days (Fig. 4f). Importantly, only the combination therapy (dT + OR0642) dramatically reduced disease progression (Fig. 4b) and prolonged survival in mice (median survival of 48 days), even after treatment arrest at day 21 (Fig. 4f), indicating strong synergy between these two therapeutic agents and a significant survival advantage. Therefore, pharmacological cotargeting of both the de novo and salvage biosynthetic pathways, thanks to the new dCK inhibitor OR0642, is well tolerated and highly effective against T-ALL leukemic cells in vivo.

### Predicting sensitivity using T-ALL patient-derived xenograft models

**Fig. 5:**
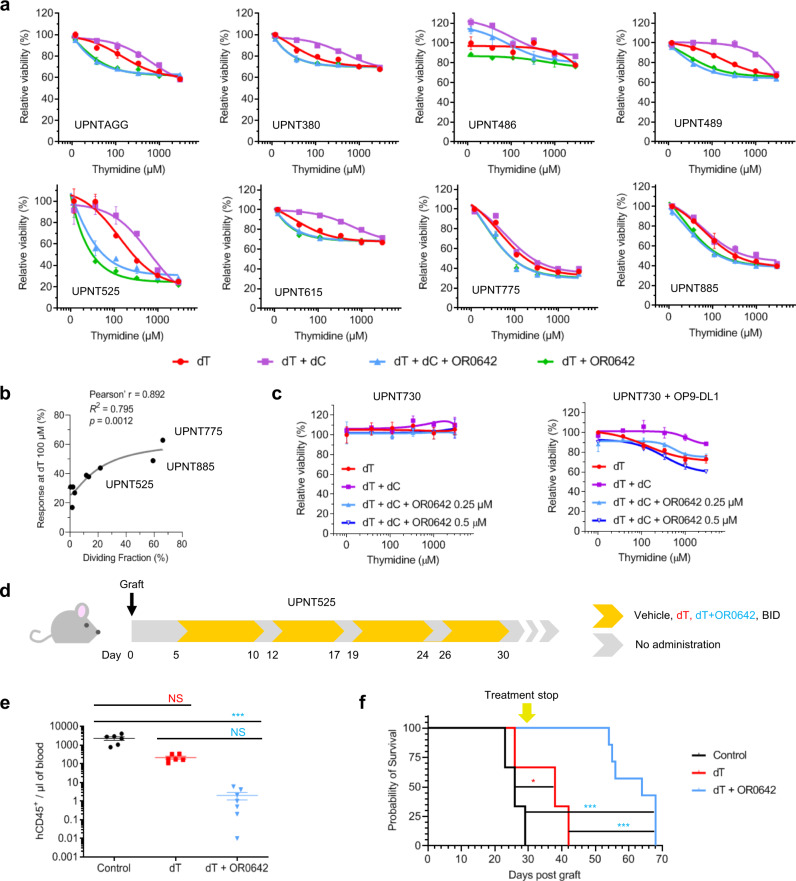
Effect of the combined therapy on PDX models. **a** Effect of a dT concentration range (10 to 3000 µM) on PDX samples in the absence or presence of dC (1 µM) and/or OR0642 (0.25 µM). Data are presented as the mean ± SD (*n* = 3). **b** Correlation between the response at dT 100 µM and the dividing fraction. Two-tailed Pearson r correlation was computed after verification of the normal distribution of the dataset (*P* = 0.0012). **c** Effect of the combined therapy on the PDX sample UPNT730 in the absence and presence of proliferative conditions (OP9-DL1 monolayer) dC (1 µM) and/or OR0642 (0.25 and 0.5 µM). Data are presented as the mean ± SD (*n* = 3). **d** Treatments on the PDX mouse model UPNT525. Schedule of the vehicle and drugs administration mice groups (vehicle (*n* = 6), dT 1.5 g/kg (*n* = 6), and dT 1.5 g/kg + OR0642 40 mg/kg (*n* = 7)) (twice/day (BID)) against UPNT525 bearing mice. **e** Effect of treatments on hCD45^+^ populations determined by flux cytometry at day 19. Data are presented as the mean ± SEM. Comparison of hCD45^+^ populations was performed using one-way ANOVA Kruskal–Wallis test and Dunn’s multiple comparison test (vehicle (*n* = 6), dT (*n* = 6), and dT + OR0642 (*n* = 7)). NS represents no statistical significance and ***P = 0.0002. **f** Survival analysis of UPNT525 bearing mice treated with vehicle and drugs. The yellow arrow highlights the end of the treatment. Median survival times were compared using Log-Rank (Mantel–Cox) Test. NS represents no statistical significance, *P = 0.02, and ***P < 0.0003.

To test the combination therapy in more relevant human T-ALL models, we used established patient-derived xenografts (PDXs) derived from primary-T-ALL samples^28,29^. We evaluated the sensitivity of 8 PDX samples ex vivo to the dCK inhibitor OR0642 combined with a concentration range of dT. Alternatively, we tested the dT + OR0642 association in the presence of dC, as such conditions allow triggering of the SP (Supplementary Fig. 3). Hence, we investigated whether the combination therapy (dT + OR0642) induced a benefit compared to dT treatment alone. As shown in Fig. 5a, the response to dT alone was heterogeneous; 3 PDXs were found to be responders, 4 were partial responders and 1 was resistant. The addition of dC partially reversed the effect of dT and increased the viability of the responder PDX cells, while the combined treatment of dT + OR0642 (in the presence or absence of dC) was able to induce a significant reduction in cellular viability. Therefore, 3 PDXs were classified as responders to the combined therapy (UPNT525, UPNT775, and UPNT885), and 5 were classified as partial responders (UPNTAGG, UPNT380, UPNT486, UPNT489, and UPNT615). We hypothesized that the intrinsic ex vivo proliferative behavior of the PDX could be the key to the efficacy of compound OR0642. To test this, we examined the correlation between the combination response (for a fixed concentration of dT at 100 µM) and the proliferative index of the PDXs (measured by CellTrace in control conditions of culture) (Fig. 5b). As expected, the PDXs exhibiting the highest dividing fraction were the best responders to dT treatment and to the combination with OR0642 (UPNT525, UPNT775, and UPNT885). To demonstrate this, we studied the sensitivity of a resistant PDX, UPNT730 (Fig. 5c), in standard culture conditions and cultured on a monolayer of OP9-DL1 stromal cells to force their proliferation. While the tested PDX was resistant to regular conditions, it was found to be sensitive to both dT and the combined therapy (dT + OR0642) under proliferative conditions, which confirmed our hypothesis. Furthermore, we compared the efficacy of the combined therapy (dT + OR0642) on two responder PDXs in regard to the standard-of-care chemotherapy (vincristine + l-asparaginase + dexamethasone) (Supplementary Fig. 16). Results showed that the new combination was at least as active (UPNT525) or significantly more potent (UPNT775) than the conventional therapy. Finally, we evaluated the efficacy of the combined therapy in vivo in a T-ALL PDX mouse model using the responder UPNT525 (Fig. 5d). The leukemic burdens were determined by flux cytometry on day 19 based on the fraction of hCD45+ cells circulating in the bloodstream of the animals. The treatment with dT alone induced a significant decrease in disease progression, but the combination therapy (dT + OR0642) greatly improved this result, decreasing the leukemic burden by more than 1000 times. As expected, the median survival of the treated groups was increased compared to that of the control group (26 days). The group treated with dT alone presented a median survival of 38 days, and the group treated with the combination therapy (dT + OR0642) impressively increased the median survival to 64 days, which shows the high efficacy of this therapy in a mouse model derived from a patient tumor.

## Discussion

In the present work, we demonstrate the proof-of-concept of this drug design approach, where the repositioning of a drug taking advantage of the identification of an “off-target” has been used as a starting point to accelerate classic drug discovery (hit identification and hit-to-lead optimization) into the automated process of designing and optimizing new drugs on this new target. The strategy has been validated in vivo in a human T-ALL PDX mouse model, a leukemia subgroup with a significant lack of treatment options for resistant and relapsed patients.

Approximately 30% of newly approved drugs for a particular treatment have been repositioned from another therapy, and such repositioning strategies are likely to become more common in cancer drug discovery^30^. There are two main strategies of drug repositioning: on-target and off-target. In on-target drug repositioning, the known pharmacological mechanism of a drug is applied to a new therapeutic purpose. In this strategy, the biological target of the drug is identical, but the disease is different, and the drug produces two different therapeutic effects. On the other hand, in off-target drug repositioning, the drug acts on new targets, out of the original scope, for new therapeutic indications. Therefore, both the targets and the indications are new.

Our previous successful off-target drug repositioning strategy for masitinib revealed how dCK could be maintained in an open conformation to potentiate the activity of nucleoside analog agents upon masitinib binding^16^. The poor complementarity of masitinib in the extended cavity of dCK led us to implement an SAR optimization program that used masitinib’s pyridine moiety (ring A) as the starting anchor point. We hypothesized that an increased level of interaction with dCK could trigger a switch of masitinib derivatives from activators to inhibitors. Such potent SP inhibitors used in combination with RNR inhibitors could efficiently fight cancer cells and prevent escape mechanisms by simultaneously inhibiting both the DNP and SP. The pyrimidine SP and DNP can compensate each other in their ability to sustain cancer cell proliferation, and a synthetic lethal phenotype can be achieved through their simultaneous inhibition^12^.

The DOTS methodology was successfully applied to design more potent dCK inhibitors starting from the dCKi1 ligand. This strategy heavily relies on computational design reinforced by experimental results obtained through methods involving medicinal chemistry, biophysics, biochemistry, structural biology, cellular biology and animal models. From the early optimization steps, we quickly confirmed our hypothesis for the very first synthesized inhibitors, dCKi1 and dCKi2, bearing a pyrimidine ring with additional amine moieties on ring A (dC mimetic). Overall, the 5 rings of masitinib (A, B, C, D, E) and 3 of the 4 linkers (B–C, C–D, D–E) were sequentially optimized using DOTS to achieve better affinity, enzymatic and cellular activity and physicochemical properties.

The key modifications that boosted the lead optimization were the replacement of the ring A pyridine by a 4,6-diaminopyrimidine, followed by the introduction of a biphenyl and/or a phenyl-pyridine for the C–D rings, an *N*-propyl on the B-C linker, a sulfonamide at the D–E linker, and various substitutions on ring D. OR0642, our final lead, is a selective and strong dCK binder that exhibits nanomolar inhibition of dCK enzymatic and cellular activity. Well tolerated in mice, OR0642 doubled the median survival rate of mice submitted to the DNP/SP combination treatment (dT + OR0642), demonstrating the potential of this strategy for T-ALL treatment. Bioluminescent imaging, used to monitor tumor progression, showed a 100-fold lower signal in mice treated with the combination therapy than in those treated with the control or individual therapies, indicating a significant decrease in systemic leukemic burden. This is the confirmation of a phenotypic synthetic lethality mechanism of action. Both individual therapies exhibited slight or no impact on tumor progression, but promising therapeutic efficacy was confirmed for the combination therapy (dT + OR0642), which dramatically reduced disease progression and prolonged mouse survival (48 versus 26 days) even after an early treatment arrest (21 days).

In the 1980s, the clinical, cytokinetic, and biochemical effects of thymidine in patients with hematologic malignancies and solid tumors were investigated^31^. Prolonged dT infusions revealed profound responses in leukemia and lymphoma patients; however, therapeutic responses to dT in these patients were, in general, limited and transient^32^. The ability of cancer cells to switch their dNTP synthesis from the DNP to the SP explains why dT delivered as a single agent showed limited efficacy in clinical trials. Therefore, our combination of dT with the dCK inhibitor OR0642 to cotarget dNTP production by the SP, which is more efficient in killing tumor cells than either treatment alone, would be a better therapeutic approach for these leukemia patients. Despite progress in the treatment of ALL, adult patients continue to have poor prognosis and clinical outcomes despite intensive therapy. Highly aggressive T-ALL is even more concerning since there are still limited treatment options^33^. The long-term survival rate is approaching 50% in T-ALL adults due to the introduction of intensive pediatric-inspired chemotherapy^34^ and better risk stratification based on minimal residual disease (MRD) monitoring; however, T-ALL is also associated with early aggressive relapses and treatment resistance^35,36^. With a relapse incidence close to 20–25% for pediatric cases and 40% for adult cases and a 5-year overall survival rate below 25% for relapsed patients, T-ALL remains a critical clinical challenge. After a patient relapse, the differences between B-ALL and T-ALL become much more important. Patients with B-ALL have several options available after relapse thanks to the development of the CD19 × CD3 bispecific T-cell engager blinatumomab^37^, the anti-CD22 antibody-drug conjugate inotuzumab ozogamicin^38^, or the chimeric antigen receptor (CAR)-T-cell therapy^39^. Other options, such as the proteasome inhibitor bortezomib^40^ and the BCL2 inhibitor venetoclax^41^, have also been reported; nevertheless, their use is currently very limited. In contrast, T-ALL patients upon relapse have limited therapeutic options, restricted to salvage chemotherapy regimens or the chemotherapy agent nelarabine, which is specifically approved for T-ALL after relapse. Due to the lack of treatment options, there are several clinical trials that are investigating agents already used for other cancers in patients with T-ALL, but their efficacy has yet to be proven^42^.

Therefore, innovative, effective and well-tolerated treatments are needed, particularly when allogenic transplant is not an option, as the median survival for patients with relapsed and refractory T-ALL is only 8 months^43^. Other studies had previously shown that pharmacological cotargeting of the DNP and the SP is efficacious against ALL models in mice^12^, but these results were mostly performed in a subcutaneous xenograft tumor model and patient-derived samples were not explored. Here we assessed the sensitivity of PDX samples to this therapeutic strategy cotargeting the DNP and the SP and showed that PDXs presenting a higher dividing fraction were the best responders to treatment with OR0642 and dT. Moreover, the combined therapy was efficient in a T-ALL PDX mouse model, doubling the median survival rate. From our convincing in vitro and animal model results, T-ALL targeting using this synthetic lethality approach represents a promising therapeutic option that we are currently investigating further. Combining OR0642 (an SP inhibitor) with dT (a DNP inhibitor) restricts cancer cells from both their nucleotide sources when the dNTP pool in the cell is sufficient for only a few minutes of DNA replication. Moreover, this synthetic lethality strategy should be highly efficient on all cells exhibiting high reliance on the SP and could be applied on other specific cancer cells showing strong dependency on dCK and SP. Critically, the aggressive nature of T-ALL, which, within days, shows outbursts from the clonal expansion of discrete tumor cells that invade the bone marrow and the blood of patients with tens of billions of blasts, confers sensitivity to antiproliferative drugs. This cardinal property of T-ALL is exemplified in the clinic by the strong and rapid debulking effect of glucocorticoids during the induction course. We surmise that oncogenic addiction to proliferation of T-ALL blasts conveys a vulnerability to dCK inhibitors and positions OR0642 as a promising candidate to treat acute leukemias. These questions will be addressed in later studies.

## Methods

### In silico stages from DOTS approach

The original design approach, relying on encoded chemical reactions and available BBs^20^, was enriched by an alternative ‘generic design’ strategy. A dictionary of functional groups, common rings (from 3-membered to 6-membered rings, decorated or not on ortho/meta/para positions) with varied C/O/N spacer lengths, was first manually designed. As in the original DOTS approach, a python script relying on the RDKit toolkit (Open-Source Cheminformatics Software, http://www.rdkit.org) was used to combine a reference ligand with a predefined attachment point to the previous generic dictionary. The post-processing stages of the designed library also remained the same: compounds with undesired substructures and unreasonable physicochemical properties were discarded, proper microspecies and tautomers were also computed using ChemAxon tools (https://chemaxon.com/). Finally, a 3D conformer of each ligand was generated and superimposed on the original dCKi1 bioactive conformation. The single difference with the original processing stage workflow concerned the removal of the diversity stage, as the goal here was to keep each designed ligand even highly close analog compounds. Then, the designed compounds were evaluated in SeeSAR Software 9 (BioSolveIT, http://www.biosolveit.de/SeeSAR) using the template-based docking mode and dCKi1 as the reference ligand. Several parameters were used to prioritize the compounds to be synthesized at each round (HYDE evaluation^44^, visual inspection of predicted binding modes, quality of torsion angles, absence of inter/intra-clashes, and physicochemical properties such as LogP). Protein-ligand interaction diagrams were calculated with the Molecular Operating Environment Software (2022.02).

### Chemical synthesis

Listing of compounds, general procedures for the synthesis and structural characterization are detailed in Supplementary Information (Supplementary Figs. 17–27 and Supplementary Note 1).

### Protein expression and purification

BL21 pRIL *Escherichia coli* cells were transformed with dCK-plasmids (dCK-C4S-S74E (for crystallization assays) and dCK-C3S-S74E (for TSA, enzymatic and ITC assays)). Protein dCK-C4S-S74E used for crystallization assays contained four mutations in order to generate better quality crystals (four solvent-exposed cysteine residues were mutated to serine; C9S, C45S, C59S and C146S). Protein dCK-C3S-S74E used for other assays contained only three mutated cysteines. The S74E mutation mimics the phosphorylated state of this serine, which favors the open conformation of the enzyme, making it competent for nucleoside binding^45^ to evaluate the binding affinity with compounds. *E. coli* cells were grown in LB media containing 100 μg/ml ampicillin and 34 μg/ml chloramphenicol at 18 °C for 20 h after induction with 1 mM IPTG.

For binding assays (TSA, ITC and crystallography) cells were re-suspended in 50 mM TRIS, pH 8, 500 mM NaCl, 30 mM imidazole and 10% glycerol buffer and one EDTA free anti-protease tablet was added (Roche). After sonication and centrifugation (30,000 × *g*), the supernatant was loaded on a 5 ml HisTrap FF column (GE healthcare) pre-equilibrated with resuspension buffer. Protein was then eluted by resuspension buffer containing 500 mM imidazole. Protein eluted fractions were mixed and concentrated. Protein was further purified by size exclusion chromatography (superdex 200 16/600, GE healthcare) in 20 mM HEPES, pH 7.5, and 200 mM NaCl before concentration to 30 mg/ml and storage at −80 °C.

For enzymatic assays, cells were re-suspended in 20 mM TRIS, pH 8 500 mM NaCl, 0.1% BRIJ-35 and 5% glycerol buffer and one EDTA free anti-protease tablet was added (Roche). After sonication and centrifugation (30,000 × *g*), the supernatant was loaded on a 5 ml HisTrap FF column (GE healthcare) pre-equilibrated with 20 mM TRIS, pH 8, 500 mM NaCl, 0.01% BRIJ-35 and 5% glycerol buffer. The protein was then eluted by the same buffer containing 250 mM imidazole after a wash containing 25 mM imidazole. The eluted fraction was applied on a HiPrep 26/10 desalting column (GE healthcare) pre-equilibrated with 20 mM TRIS, pH 8, 500 mM NaCl, 5% glycerol and 0.01% BRIJ-35 buffer. Finally, protein in a 20% glycerol buffer was flash frozen in liquid nitrogen and stored at −80 °C.

### Thermal shift assay

Thermal shift assay (TSA) experiments were performed in 384-well PCR white plates in the assay buffer containing 20 mM HEPES, pH 7.5 and 200 mM NaCl. Final concentrations were adjusted to 5 μM of protein (dCK-C3S-S74E), 50 μM of compound (final 2% DMSO) and dye diluted to 1:1000, as recommended by manufacturer (Protein Thermal Shift Dye, Thermo Fisher Scientific). Controls with compounds alone or compounds with dye were performed to verify that compounds do not present autofluorescence. Plates were centrifuged at 200 × *g* for 2 min at 4 °C. Thermal melting experiments were carried using a CFX-384 Connect RT-PCR (Bio-Rad). Plates were first equilibrated at 25 °C; then, the plates were heated from 25 to 95 °C by 0.5 °C/min steps. Raw fluorescence data were treated, and the melting temperatures (*T*_m_) were calculated using CFX Manager 3.1 software (Bio-Rad). Δ*T*_m_ represents the difference in *T*_m_ between the protein in the presence of compound and the protein alone (both at 2% DMSO). Each experiment was performed at least twice (with technical triplicates) and data are presented as the mean ± SD.

### Isothermal titration calorimetry

ITC was performed to evaluate the binding between dCK-C3S-S74E and the selected compounds. Purified protein and compounds were diluted in the buffer assay containing 20 mM HEPES, pH 7.5, and 200 mM NaCl. Titrations were carried out on a MicroCal ITC200 microcalorimeter (Malvern) at 25 °C. A first small injection (0.2 μl) was included in the titration protocol in order to remove air bubbles trapped in the syringe prior titration and/or take into account syringe pre-dilution in the cell during equilibration. To avoid compounds solubility problems at high concentration reverse titrations were performed using 15 injections of the titrant (400 µM protein in the syringe) into the analyte (80 μM compound in the cell). Raw data were scaled after setting the zero to the titration saturation heat value. Integrated raw ITC data were fitted to two sites nonlinear least squares fit model using MicroCal Origin 9.1 (Origin Lab) in agreement to the thermogram biphasic profile and the presence of a second binding site confirmed by the X-ray structures (Supplementary Fig. 28). Each experiment was performed three times and data are presented as the mean ± SD.

### Enzymatic assay

Kinetic analyses were performed using a spectrophotometric continuous enzymatic-coupled assay using pyruvate kinase (PK) and lactate dehydrogenase (LDH). Analysis of the effect of compounds on dCK-C3S-S74E activity was performed using dC as substrate and UTP/ATP as phosphate donors. UTP is the physiological dCK-phosphate donor^46^ and it was used to determine activation versus inhibition behavior. ATP was used to calculate IC_50_ values, as it allows the analysis of a dose response curve. The PK/LDH coupled assay is based on the conversion of phosphoenolpyruvate (PEP) and UDP/ADP to pyruvate and UTP/ATP by PK and the subsequent conversion of pyruvate to lactate by LDH. The latter step requires NADH, which is oxidized to NAD^+^. NADH is fluorescent (excitation at 337 nm and emission at 460 nm), but not NAD^+^. Thus, the measurement of fluorescent decrease at 460 nm is a measure of kinase activity. dCK experiments were performed in a buffer containing 50 mM HEPES, pH 7.5, 5 mM MgCl_2_, 1 mM DTT, and 100 mM KCl. Buffer was supplemented with PK (0.01 U/µl), LDH (0.02 U/µl), PEP (1 mM), NADH (300 µM) and compounds at varying concentrations. For UTP assays, protein concentration was 2 μM, substrate 200 µM (dC), and UTP 1 mM. For ATP assays, protein concentration was 100 nM (and 50 nM when indicated), substrate 100 µM (dC), and ATP 1 mM. All measurements were performed on a microplate reader Pherastar FS (BMG Labtech). All assays were performed in triplicate and each experiment was performed at least twice. The data are presented as mean ± SD. The results were plotted as kinetics and the velocity was determined for each condition as the slope of the linear range of the curve. For each compound concentration, the velocity of the reaction (according to *V* = d[*P*]/d*t*) was normalized with respect to the drug free control and velocity ratios were compared. IC_50_ values corresponding to enzymatic inhibition in ATP were calculated by nonlinear regression curves using GraphPad Prism software 9.4.0. Technical limit for IC_50_ calculation (in ATP) was determined by protein concentration (100 nM or 50 nM), so it was not possible to determine IC_50_ values lower than these values.

### Crystallization

Structures were determined using UDP since co-crystallization with the triphosphate form would result anyway in hydrolysis to the diphosphate form after several days of assay. Prior to co-crystallization assays, UDP (5 mM), MgCl_2_ (5 mM) and compound (1 mM) were added to the protein stock dCK-C4S-S74E (12 mg/ml). Crystals were obtained using hanging drop vapor diffusion at 12 °C. Drops were constituted of 1 μl of protein/UDP/MgCl_2_/compound mix and 0.5/1/1.5 μl of the reservoir solution containing 50–80 mM HEPES, pH 7.5 and 0.8–1.2 M trisodium citrate. Prior to data collection, crystals were rapidly soaked in mineral oil and subsequently flash frozen in liquid nitrogen.

### Data collection and processing

X-ray data were collected at the European Synchrotron Radiation Facility (Grenoble, France) on beamlines ID30A-1 and ID30B and at the French Synchrotron SOLEIL (Paris, France) on beamlines Proxima1 and Proxima2. The data reduction and scaling were performed using the XDS suite^47^ or DIALS^48^ via the XIA2 pipeline^49^. The high-resolution cut-off was defined by the CC_1/2_ metric automatically selected by XDS (marked resolution shell)^50^. Data collection and refinement statistics are presented in Supplementary Tables 4 and 5.

### Structure determination and refinement

The structures of dCK-C4S-S74E were solved by molecular replacement using PHASER^51^ and the published dCK-C4S-S74E structure in complex with the inhibitor DI-39 (PDB ID: 4KCG) was used as starting model. The initial molecular replacement solution was further refined using REFMAC5^52^, alternating with cycles of manual rebuilding in COOT^53^. After a few cycles of refinement, ligands were drawn using COOT two-dimensional sketcher to generate 3D coordinates files later imported in the model during refinement. Their associated CIF restraint dictionary required by REFMAC5 was generated from COOT using PRODRG^54^. Structure factors have been deposited at the Protein Data Bank (accession codes: 7ZI1 (dCKi1), 7ZI2 (dCKi2), 7ZI3 (OR0642), 7ZI5 (OR0274), 7ZI6 (OR0325), 7ZI7 (OR0345), 7ZI8 (OR0602), 7ZI9 (OR0624), 7ZIA (OR0634), and 7ZIB (OR0635)). A residual electron density parallel to the C–D rings was observed for several compounds (Supplementary Fig. 28). This indicates a potential low affinity second binding site confirmed by the biphasic ITC thermograms observed with dCKi1 and dCKi2 (Supplemental Fig. 2). The addition of a N-propyl moiety on compounds filled the hydrophobic cavity, shifted the compound and prevented the binding of the second molecule (Supplemental Fig. 28f).

### c-KIT cellular assay

Ba/F3 cells were grown at 37 °C in RPMI 1640 with l-glutamine, supplemented with 100 U/ml penicillin, 100 mg/ml streptomycin, and 10% heat-inactivated fetal calf serum. The generation of Ba/F3 cells expressing wild-type human c-KIT has been previously described by us^55,56^. For the assay of Ba/F3 cell proliferation, plates were seeded with a total of 10,000 cells per well. Cells were supplemented, or not, with either 0.1% IL3 or 250 ng/ml murine SCF. IL3 was obtained from conditioned medium from X63-omIL-3 cells (obtained from Fritz Melchers laboratory (Berlin, Germany)) and murine SCF, which activates c-KIT, was purified from the conditioned medium of SCF-producing CHO cells. Cells were supplemented with compounds at varying concentrations (final 0.1% DMSO). Plated cells were grown for 48 or 72 h at 37 °C and then incubated with 10 µl/well of CellTiter Blue reagent (Promega) for 3 h at 37 °C. The amount of reduced dye formed by metabolically active cells was quantified by its fluorescence measured at 560 nm (excitation) and 590 nm (emission) in a microplate reader (FLUOstar Omega, BMG Labtech). A blank well without cells was used as a background control. Each experiment was performed at least three times (with technical triplicates) and data are presented as the mean ± SD.

### dCK cellular assay

The dCK cellular assay was set up to assess the ability to inhibit dCK activity in a cellular model. The model was developed with a T-cell acute lymphoblastic leukemia cell line (CCRF-CEM), because of its high dependence on the SP, thus on dCK activity. Cellular assays were performed to assess the inhibition of cell proliferation under two conditions: (i) in the presence of the compound alone to determine the nonspecific effect on cells, independent of dCK (nonspecific toxicity) and (ii) in the presence of an inhibitor of the DNP to determine the ability of compounds to inhibit dCK and decrease tumor proliferation (activity on dCK). The inhibitor of the DNP chosen for the experiments was thymidine (dT), a physiological inhibitor of ribonucleotide reductase which arrests cell proliferation in phase S. The dTTP produced via thymidine kinase from dT acts as a RNR inhibitor by an allosteric regulation of the R1 subunit. The addition of the nucleoside deoxycytidine (dC) in the medium allows the rescue of cell proliferation by passing only through the salvage pathway, mediated by dCK. dC needs to be added exogenously to mimic physiological concentrations because it is not present in the cell culture medium, on the contrary to mouse model, in which it is naturally present in the serum. Therefore, cell proliferation under these conditions (+dT +dC) is dCK dependent and it is possible to determine the IC_50_ of dCK inhibitors on the inhibition of cell proliferation. Results are expressed as % cell proliferation relative to the control with DMSO.

CCFR-CEM cells (obtained from O. Hermine laboratory, Paris, France) were grown at 37 °C in RPMI 1640 with L-glutamine, supplemented with 10% heat-inactivated fetal calf serum. The experiments were carried out on 96-well plates under the following conditions: 14,000 cells per well, RPMI 1640 culture medium supplemented with 10% heat-inactivated fetal calf serum, 200 μM of dT, 1 μM of dC and a range of dilutions of each compound ranging from 40 μM to 10 pM, depending on the activity of the compounds (final 0.2% DMSO). Cells were incubated for 72 h under these different conditions and finally, incubated with 10 µl/well of CellTiter-Blue reagent (Promega) for 4 h at 37 °C. The amount of reduced dye formed by metabolically active cells was quantified by its fluorescence measured at 560 nm (excitation) and 590 nm (emission) in a microplate reader (CLARIOstar, BMG Labtech). A blank well without cells was used as a background control. Each experiment was performed at least three times (with technical triplicates) and data are presented as the mean ± SD.

### Kinome assay

Kinome profiling was provided by Eurofins DiscoverX Corporation. Determination of the % control activity for 97 kinases was obtained through an active site-directed competition binding assay that quantitatively measure interactions between compound and kinases. OR0642 was used at 1 μM.

### CCRF-CEM CRISPR-Cas9 cell line deficient for dCK

Stable CCRF-CEM-CRISPR-sgRNA-dCK^−^ cell line was established by lentiviral transduction using purified lentiviral particles (Horizon Discovery) and blasticidin selection (10 µg/ml). Doxycycline-inducible Cas9 integration was confirmed after doxycycline exposition (1 µg/ml) by western blot with CRISPR-Cas9 antibody (Santa Cruz, SC-517386, 1:1000). Cas9-expressing cell line was further transduced with human DCK sgRNA purified lentiviral particles (Horizon Discovery, sequences are provided in Supplementary Table 6) and puromycin selection (1 µg/ml). Finally, two successive gemcitabine selections (2 µM for 3 days and 10 µM for 2 days) were performed to obtain the cell line deficient for dCK. Experiments were performed as in the dCK cellular assay.

### Nucleotides pool measurements

CCRF-CEM cells exposed to compounds (dT (200 µM) and/or dC, OR0642, DI-87 and DI-39 (1 µM)) for 0 and 24 h were washed, counted and pelleted. Pellets were extracted with the cold sampling solution (acetonitrile (4) / methanol (4)/125 mM formic acid (2)), vortexed and placed 1 h at −20 °C. Then centrifuged and evaporated. Central metabolites were separated on an ionic chromatography column IonPac AS11 (250 × 2 mm i.d.; Dionex). Solvent used was KOH at a flow rate of 350 µl/min. The column was then equilibrated for 6 min at the initial conditions before the next sample was analyzed. The volume of injection was 15 µl. High-resolution experiments were performed with an ICS5000+, ion chromatography system (Dionex) system coupled to an Orbitrap Qexactive+ mass spectrometer (Thermo Fisher Scientific, Waltham) equipped with a heated electrospray ionization probe. MS analyses were performed in negative FTMS mode at a resolution of 140,000 (at 400 *m*/*z*) in full-scan mode, with the following source parameters: 325 °C for the capillary temperature, 380 °C for the source heater temperature, 50 a.u. (arbitrary unit) for the sheath gas flow rate, 5 a.u. for the auxiliary gas flow rate, 50% for the S-Lens RF level, and 2.75 kV for the source voltage. Metabolites were determined by extracting the exact mass with a tolerance of 5 ppm. Each experiment was performed three times.

### Cell cycle analysis, Annexin V assays and γH2A.X measurements

CCRF-CEM cells were seeded at 100,000 cells/ml, and treated with DMSO (0.01%) as vehicle control or dT (200 μΜ), and/or dC, OR0642, DI-87 and DI-39 (1 μΜ). For cell cycle analysis, cells were harvested after 24 h, fixed in 4% paraformaldehyde, permeabilized with 0.15% Triton X100, and DNA content was determined using 1 μg/ml DAPI. For apoptosis assay, cells were harvested after 72 h and cell death was assayed using Annexin V-APC and 7AAD following manufacturer’s instructions (Biolegend, 640930). For γH2A.X measurements, cells were harvested after 24 h, fixed in 4% paraformaldehyde and permeabilized with 0.15% Triton X100. Then, cells were stained with the phospho-H2AX (pS139 H2AX, Biolegend, 613402, 1:1000) specific primary antibody, Alexa-Fluor 488 secondary antibody (ThermoFisher, A21202, 1:1000) and DAPI. All acquisitions were performed on a BD Fortessa cytometer, and analysis of flow cytometry data was performed using FlowJo™ Software 10.7.1 (BD Life Sciences). Each experiment was performed three times. Figures exemplifying the gating strategy are provided in the Supplementary Information (Supplementary Figs. 29–31).

### Immunocytochemistry

Cells were seeded, treated and stained as described for flow cytometry analysis, and then spread on a microscope slide using cytospin. Slides were mounted with VECTASHIELD mounting medium with DAPI (Vector Laboratories). Confocal images were captured using a LSM 510 Meta microscope at x63 magnification.

### Evaluation of the in vivo toxicity and pharmacokinetics in mice

Swiss male mice were provided by Janvier Labs and animals were maintained under controlled environmental conditions (temperature (22 ± 2 °C) and humidity (40–60%)) in conventional husbandry on a 12 h light and 12 h dark cycle. To evaluate the in vivo toxicity selected compounds were injected by intraperitoneal (IP) route to 7 weeks-old male Swiss mice (*n* = 2) at 10, 20, 40, 50, or 80 mg/kg by unique injection. Vehicle was saline solution (0.9% NaCl) supplemented with 10% Kolliphor EL. Compounds were administered in their hydrochloride salt forms. Mice were then observed closely for the next one hour, then every hour for 8 h. Observations were continued for 72 h for signs of pain or toxicity such as change in the behavior and in the activity in the cage (decreased locomotion) or change in the appearance (curved back, eyes half-closed, bristle hair, hips hollowed out). In vivo toxicity of compound OR0642 at 40 and 80 mg/kg was also assessed in the NOD-SCID-IL2Rγc null (NSG) mice model for 2 weeks in the presence and absence of dT to confirm the safety of the compound. For pharmacokinetic studies, plasma concentrations were assessed at 15, 30, 60 min, 2, 5 and 8 h, following IP administration (10 ml/kg) of 10, 20, or 40 mg/kg of compounds to male Swiss mice (*n* = 3 mice per time point). Blood samples were collected and spun at 10,000 × *g* prior to collecting the plasma supernatants. All plasma samples were frozen at −80 °C before sample processing. 13 μl of each plasma sample was mixed with 33 μl of acetonitrile to precipitate proteins and extract the compound. Samples were vortexed for 5 min and then placed in an ultrasonic bath for 1 min. The precipitated proteins were sedimented by centrifugation (15,000 × *g*, 5 min, 16 °C). Supernatants were transferred to a microplate to be analyzed by LC–MS/MS. All samples were analyzed with a UHPLC coupled to a Shimadzu LC-MS 8030 triple quadrupole. The solutions of a standard range, prepared with plasma from untreated mice, were analyzed in the same series of injections.

### Animal models

NOD-SCID-IL2R common-γ-chain-knockout mice (NSG) were purchased from Charles River and maintained under pathogen-free conditions on a 12 h light and 12 h dark cycle. Temperature was maintained between 20 and 24 °C and the hygrometry between 40 and 60%. Sex was not considered in the study design and studies were realized in male mice, as toxicity and pharmacokinetics were performed previously in male mice. Healthy 6–9-week-old male mice received 100,000 luciferase transduced-CCRF-CEM cells in the tail vein on Day 0 and were randomly assigned in four groups to IP injections from Day 3 (Vehicle (*n* = 8), dT (*n* = 7), OR0642 (*n* = 9) and dT + OR0642 (*n* = 9)). Treatments were administered during 19 days at cycles of 5 days (BID) and 2 days (QD). Doses administered were: dT at 1.5 g/kg, OR0642 at 40 mg/kg and dT + OR0642 at 1.5 g/kg + 40 mg/kg, respectively. Vehicle was saline solution (0.9% NaCl) supplemented with 10% Kolliphor EL. OR0642 was administered in its hydrochloride salt form (OR0642-HCl). Drug administration protocol is detailed in Fig. 4a. Bioluminescence analysis was performed once/week using PhotonIMAGER Optima (Biospace Lab) following addition of endotoxin-free luciferin (30 mg/kg, Promega). Peripheral blood was obtained at Day 21 to determine the fraction of human blasts using flow cytometry. Mononuclear cells were labeled with Pacific blue-conjugated anti-hCD45 (Biolegend, 304029, 1:50), APC eFluor780-conjugated anti-mCD45 (ThermoFisher Scientific, 47-0451-82, 1:200) and live/dead Fixable Far Red Dead Cell Stain kit (ThermoFisher Scientific, L10120) to determine the fraction of human blasts (Live Dead^−^/hCD45^+^/mCD45^−^ cells) using flow cytometry. Analyses were performed on a Life Science Research Fortessa flow cytometer with DIVA software 9.0.1 (BD Biosciences). The number of ALL cells/L peripheral blood was determined by using CountBright beads (Invitrogen, C36950) using described protocol. Daily monitoring of mice for symptoms of disease (ruffled coat, hunched back, weakness and reduced motility) determined the time of killing for injected animals with signs of distress. Patient-derived xenograft was generated from a primary-T-ALL (UPNT525) sample^28,29^. Briefly, we injected 1,000,000 cells in 100 μl of PBS by intravenous (IV) retro orbital injection in NSG mice. At day 5 post transplantation, mice were randomly assigned into treatment groups and treated with vehicle (*n* = 6), dT at 1.5 g/kg (*n* = 6) or dT at 1.5 g/kg + OR0642 at 40 mg/kg combination (*n* = 7) by IP injection 5 days a week (BID). Drug administration protocol is detailed in Fig. 5d. Disease propagation was monitored at day 19 by hCD45 detection in peripheral blood. Daily monitoring of mice for symptoms of disease (ruffled coat, hunched back, weakness and reduced motility) determined the time of killing for injected animals with signs of distress. Kaplan–Meier curves, bioluminescence quantifications and statistical analyses were generated using Prism (GraphPad Software 8.0.2). Figures exemplifying the gating strategy are provided in the Supplementary Information (Supplementary Figs. 32 and 33).

### Ex vivo testing on PDX samples

Bone marrow-infiltrating blasts from terminally mice bearing PDX as stated above were collected as established before^28^. Extensive analysis (immunophenotype and molecular analysis) of matched primary and PDX samples used in the study are shown in Supplementary Table 7, showing that they remain stable. Briefly, after euthanasia, bone marrows from tibiae, hips, femurs, and vertebrae were isolated and blasts collected for subsequent ex vivo experiments. All samples used contained ≥90% blasts as determined by flow cytometry. For the cytotoxic evaluation of OR0642, blasts were then stained with CellTraceTM Violet (CTV, Thermo Fisher Scientific, C34557) prior seeding at 10,000 cells into 96-well plates under normal conditions or pre-plated with OP9-DL1 cells^57^. Cells were cultured in RPMI 1640 supplemented with 20% fetal bovine serum, 50 µg/ml streptomycin, 50 UI penicillin, 4 mM L-glutamine, and a cocktail of human cytokines as previously established (50 ng/ml human stem cell factor, 20 ng/ml hFLT3-L, 10 ng/ml hIL-7 and 20 nM insulin (Miltenyi Biotec))^28^. An escalation dose of dT (0 to 3,000 µM) was used as a control to evaluate the blasts sensitivity to thymidine-induced inhibition of the deoxyribonucleotide metabolism pathway. Rescue by dC supplementation (1 µM) was also evaluated. OR0642 was then tested alone, or combined with dT or dT + dC at two concentrations (0.25 and 0.5 µM). Cell viability was evaluated by flow cytometry 72 h post-treatment. Proliferation indexes were calculated using the detection of CTV in the control replicates (DMSO) and the proliferation modeling tool of FlowJo™ Software 10.7.1 (BD Life Sciences). A control peak of undivided CTV-stained cells was systematically used to set the undivided fraction.

### Statistics

For enzymatic and TSA assays data are representative of at least two independent experiments with technical triplicates and data are presented as the mean ± SD. For ITC and cellular assays (dCK, c-KIT and CRISPR models) (cell cycle, nucleotide pool, Annexin V and DNA damage assays) each experiment was performed at least three times with technical triplicates for cellular models and data are presented as the mean ± SD. Statistical comparison of cellular assays was performed using two-tailed unpaired *t* test (GraphPad Prism Software (9.4.0)). IC_50_ values (enzymatic and cellular assays) were calculated by nonlinear regression curves using GraphPad Prism Software (9.4.0). Pearson r correlation (two-tailed) was computed after verification of the normal distribution of the dataset. Bioluminescence data were presented as the mean ± SEM and comparison was performed using two-way ANOVA test and Turkey multiple comparison test. Comparison of bioluminescence data at day 21 and hCD45^+^ populations (day 19 and day 21) were performed using one-way ANOVA Kruskal–Wallis test and Dunn’s multiple comparison test. Median survival times were compared using log-rank (Mantel–Cox) test. *P* values less than 0.05 were considered significant. Statistical analyses for animal studies were generated using GraphPad Prism Software (8.0.2).

### Reporting summary

Further information on research design is available in the Nature Portfolio Reporting Summary linked to this article.

## Supplementary information

Supplementary information

Reporting Summary

## Data Availability

Structural data for dCK in complex with dCKi1, dCKi2, OR0642, OR0274, OR0325, OR0345, OR0602, OR0624, OR0634, and OR0635 have been deposited in the Protein Data Bank (PDB), with the accession codes 7ZI1, 7ZI2, 7ZI3, 7ZI5, 7ZI6, 7ZI7, 7ZI8, 7ZI9, 7ZIA, and 7ZIB, respectively. The starting model for molecular replacement is also available in the PDB (4KCG). The other supplementary data that support the findings of this study are available from the corresponding author upon request. Source data are provided with this paper.

## Source data

Source Data

## Code availability

The DOTS scripts used in this study to automatically build chemical libraries have been deposited in the Zenodo database under accession code 10.5281/zenodo.7802787.
