# Supplementary material for: From a drug repositioning to a structure-based drug design approach to tackle acute lymphoblastic leukemia
Source: Nat Commun. 2023 May 29;14:3079. doi: 10.1038/s41467-023-38668-2 (PMC10227015; doi:10.1038/s41467-023-38668-2)
Supplement: Supplementary file 1 — Supplementary information [file 41467_2023_38668_MOESM1_ESM.pdf]

Supplementary information

From a drug repositioning to a structure-based drug design approach to tackle acute lymphoblastic leukemia

Magali Saez-Ayala*, Laurent Hoffer, Sébastien Abel, Khaoula Ben Yaala, Benoit Sicard, Guillaume P. Andrieu, Mehdi Latiri, Emma K. Davison, Marco A. Ciufolini, Paul Brémond, Etienne Rebuffet, Philippe Roche, Carine Derviaux, Edwige Voisset, Camille Montersino, Remy Castellano, Yves Collette, Vahid Asnafi, Stéphane Betzi, Patrice Dubreuil*, Sébastien Combes* and Xavier Morelli*.

<u>**Supplementary Tables:**</u>

Supplementary Table 1. Summary of 74 masitinib derivatives synthesized (compound name, chemical structure, and results obtained in experimental evaluations by Thermal Shift Assay, Enzymatic Assays, and dCK Cellular Assay).

Supplementary Table 2. Results of the acute toxicity studies and plasma pharmacokinetic parameters.

Supplementary Table 3. KINOMEscan™ Profiling of OR0642.

Supplementary Table 4. Data collection and refinement statistics of crystal structures of dCK in complex with dCKi1, dCKi2, OR0642, OR0274, and OR0325.

Supplementary Table 5. Data collection and refinement statistics of crystal structures of dCK in complex with OR0345, OR0602, OR0624, OR0634, and OR0635.

Supplementary Table 6. Sequences of human lentiviral DCK sgRNA.

Supplementary Table 7. Stability of oncogenetic lesions and the immunophenotype in patient primary vs patient-derived xenograft samples.

<u>**Supplementary Figures:**</u>

Supplementary Figure 1. Structure of dCK in complex with derivatives dCKi1 (PDB 7ZI1) and dCKi2 (PDB 7ZI2).

Supplementary Figure 2. Representative calorimetric titrations determined by Isothermal Titration Calorimetry corresponding to the interaction of dCK with dCKi1 and dCKi2.

Supplementary Figure 3. dCK cellular assay performed to assess the ability of compounds to inhibit dCK activity in a cellular model (CCRF-CEM).

Supplementary Figure 4. Effect of selected compounds on dCK enzymatic activity in the presence of UTP.

Supplementary Figure 5. Structure of dCK in complex with derivatives OR0274 (PDB 7ZI5), OR0325 (PDB 7ZI6), and OR0345 (PDB 7ZI7).

Supplementary Figure 6. Structure of dCK in complex with derivatives OR0602 (PDB 7ZI8), OR0624 (PDB 7ZI9), OR0634 (PDB 7ZIA), and OR0635 (PDB 7ZIB).

Supplementary Figure 7. Structure of dCK in complex with OR0642.

Supplementary Figure 8. Effect of selected compounds on the c-KIT cellular model.

Supplementary Figure 9. OR0642 effect on CCRF-CEM cell line.

Supplementary Figure 10. OR0642 loss its activity on the CCRF-CEM CRISPR Cas9 cell line deficient for dCK.

Supplementary Figure 11. Evaluation of the negative control compound OR0659 closely related to OR0642.

Supplementary Figure 12. The combination treatment dT+OR0642 induces cell cycle arrest.

Supplementary Figure 13. The combination treatment dT+OR0642 induces nucleotide imbalance.

Supplementary Figure 14. dCK inhibitors combined with dT induce DNA double strand breaks.

Supplementary Figure 15. OR0642 synergizes with dT to induce apoptosis.

Supplementary Figure 16. Ex vivo evaluation of the combined therapy vs conventional chemotherapy (VAD).

Supplementary Figure 17. Synthesis of *N*-(3-((4-(4-aminopyrimidin-2-yl)thiazol-2-yl)amino)-4-aryl) benzamide series.

Supplementary Figure 18. Synthesis of *N*-([1,1'-biaryl]-3-yl)-4-(4-aminopyrimidin-2-yl)thiazol-2-amine series.

Supplementary Figure 19. Synthesis of 4-(4-aminopyrimidin-2-yl)-*N*-(3-(arylethynyl)aryl)thiazol-2-amine series.

Supplementary Figure 20. Synthesis of 4-(4-aminopyrimidin-2-yl)-*N*-(2-methyl-5-(4-((4-methylpiperazin-1-yl)methyl)phenoxy)phenyl)thiazol-2-amine and 4-(4-aminopyrimidin-2-yl)-*N*-(2-methyl-5-(4-((4-methylpiperazin-1-yl)methyl)benzyloxy)phenyl)thiazol-2-amine.

Supplementary Figure 21. Synthesis of *N*-(3-((4-(4,6-diaminopyrimidin-2-yl)thiazol-2-yl)amino)-4-methylphenyl)-4-((4-methylpiperazin-1-yl)methyl)benzamide dCKi2.

Supplementary Figure 22. Linear synthesis of *N*-([1,1'-biaryl]-3-yl)-4-(4,6-diaminopyrimidin-2-yl)thiazol-2-amine series.

Supplementary Figure 23. Convergent synthesis of *N*-([1,1'-biaryl]-3-yl)-4-(4,6-diaminopyrimidin-2-yl)thiazol-2-amine series and analogues.

Supplementary Figure 24. Chemical structures of DI-87 enantiomers; (*R*)-DI-87 and (*S*)-DI-87.

Supplementary Figure 25. ^{1}H NMR spectrum of OR0642.

Supplementary Figure 26. ^{13}C NMR spectrum of OR0642.

Supplementary Figure 27. ^{19}F NMR spectrum of OR0642.

Supplementary Figure 28. Residual electron densities in dCKi1, dCKi2, OR0274, and OR0325 structures.

Supplementary Figure 29. Flow cytometry gating strategy for cell cycle analysis.

Supplementary Figure 30. Flow cytometry gating strategy for apoptosis assay.

Supplementary Figure 31. Flow cytometry gating strategy for DNA damage assay.

Supplementary Figure 32. Flow cytometry gating strategy in the CDX model.

Supplementary Figure 33. Flow cytometry gating strategy in the PDX model.

Supplementary Notes:

Supplementary Note 1. Synthesis of compounds. General procedures for the synthesis and characterization of new compounds.

Supplementary Table 1. Summary of 74 masitinib derivatives synthesized. Compound name, chemical structure, and results obtained in experimental evaluations by Thermal Shift Assay, Enzymatic Assays, and dCK Cellular Assay are detailed. Stabilization of dCK determined by Thermal Shift Assay is expressed in +ΔTm (°). dCK enzymatic activity determined in the presence of UTP is expressed with the letters A or/and I (Activation *vs* Inhibition). dCK enzymatic inhibition in the presence of ATP is expressed in IC_{50} (nM) (^{A} [dCK] = 50 nM, instead of 100 nM). Inhibition in dCK Cellular Assay is expressed in IC_{50} (nM) (cellular proliferation inhibition on the T-cell acute lymphoblastic leukemia cell line CCRF-CEM in the presence of 200 µM dT and 1 µM dC). Data are presented as the mean of independent experiments ± SD.

COMPOUND NAME	CHEMICAL STRUCTURE	SMILES	THERMAL SHIFT ASSAY +ΔTm (°)	ENZYMATIC ASSAY (Activation/ Inhibition) IC_{50} (nM)	dCK CELLULAR ASSAY IC_{50} (nM)
**dCKi1**	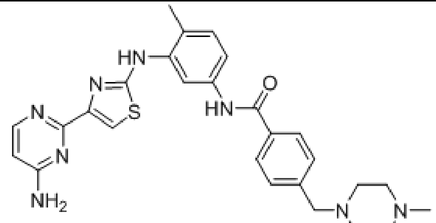	NC1=NC(C2=CSC(NC3=CC(NC(C4=CC=C(CN5CCN(C)CC5)C=C4)=O)=CC=C3C)=N2)=NC=C1	14.3 ± 0.4	A/I 2661 ± 608	6954 ± 2532
**dCKi2**	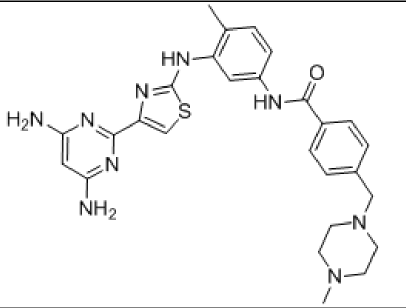	NC1=NC(C2=CSC(NC3=CC(NC(C4=CC=C(CN5CCN(C)CC5)C=C4)=O)=CC=C3C)=N2)=NC(N)=C1	17.3 ± 0.2	A/I 903 ± 37	8906 ± 3339
**OR0105**	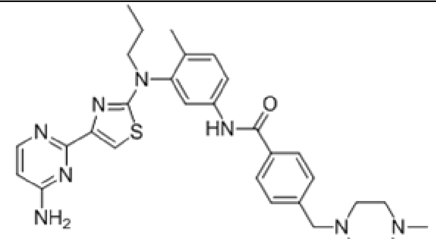	CCCN(C3=CC(NC(C4=CC=C(CN5CCN(C)CC5)C=C4)=O)=CC=C3C)C1=NC(C2=NC=CC(N)=N2)=CS1	14.5 ± 0.2	I 2135 ± 49	1620 ± 254
**OR0125**	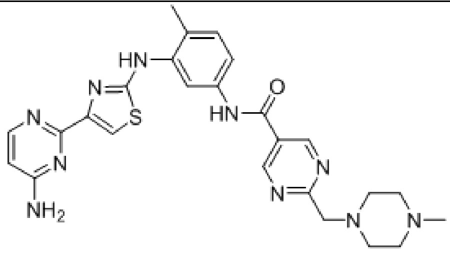	NC1=NC(C2=CSC(NC3=CC(NC(C4=CN=C(CN5CCN(C)CC5)N=C4)=O)=CC=C3C)=N2)=NC=C1	10.3 ± 0.0		37669 ± 4383
**OR0143**	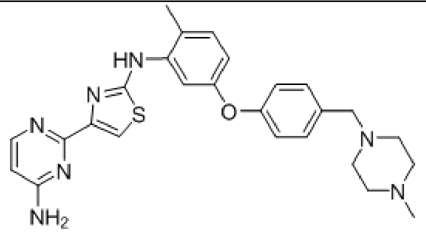	NC1=NC(C2=CSC(NC3=CC(OC4=CC=C(CN5CCN(C)CC5)C=C4)=CC=C3C)=N2)=NC=C1	13.2 ± 0.2		2794 ± 1736

**OR0146**	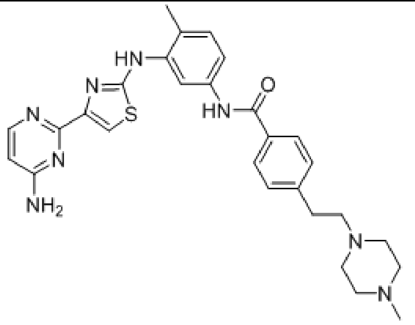	NC1=NC(C2=CSC(NC3=CC(NC(C4=CC=C(CCN5CCN(C)CC5)C=C4)=O)=CC=C3C)=N2)=NC=C1	14.8 ± 0.4	A/I 3460 ± 349	14622 ± 1191
**OR0153**	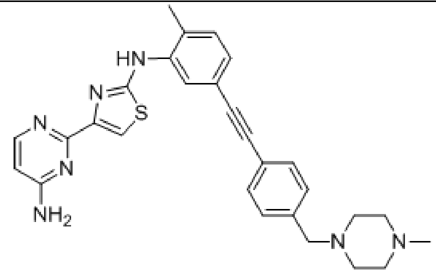	NC1=NC(C2=CSC(NC3=CC(C#CC4=CC=C(CN5CCN(C)CC5)C=C4)=CC=C3C)=N2)=NC=C1	16.9 ± 0.4	A/I 2043 ± 684	2175 ± 388
**OR0155**	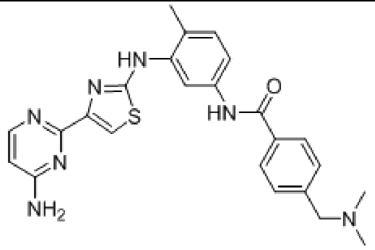	NC1=NC(C2=CSC(NC3=CC(NC(C4=CC=C(CN(C)C)C=C4)=O)=CC=C3C)=N2)=NC=C1	10.9 ± 0.4	A/I 7782 ± 2326	13809 ± 394
**OR0156**	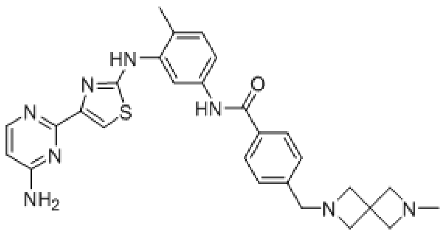	NC1=NC(C2=CSC(NC3=CC(NC(C4=CC=C(CN5CC6(CN(C)C6)C5)C=C4)=O)=CC=C3C)=N2)=NC=C1	12.6 ± 0.1	A/I 6385 ± 2075	>40000
**OR0232**	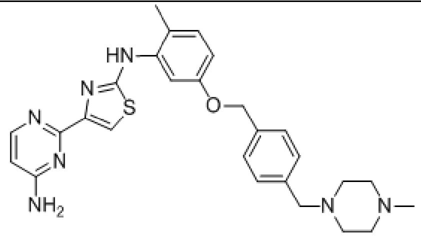	NC1=NC(C2=CSC(NC3=CC(OCC4=CC=C(CN5CCN(C)CC5)C=C4)=CC=C3C)=N2)=NC=C1	15.2 ± 0.0	A/I 1937 ± 529	3456 ± 638
**OR0237**	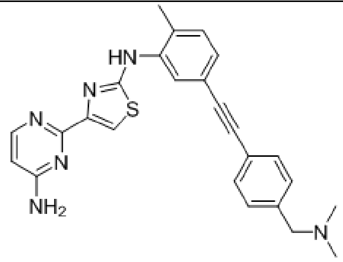	NC1=NC(C2=CSC(NC3=CC(C#CC4=CC=C(CN(C)C)C=C4)=CC=C3C)=N2)=NC=C1	15.3 ± 0.5	A/I 2140 ± 387	3345 ± 1453
**OR0239**	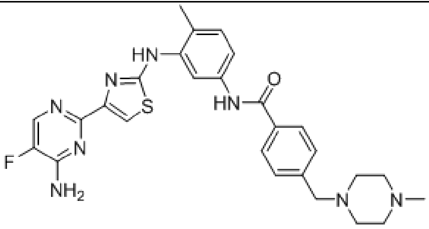	NC1=NC(C2=CSC(NC3=CC(NC(C4=CC=C(CN5CCN(C)CC5)C=C4)=O)=CC=C3C)=N2)=NC=C1F	14.3 ± 0.4	A/I 3354 ± 1067	14845 ± 1578

**OR0241**	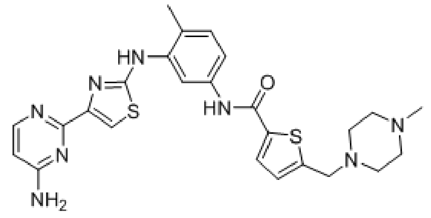	NC1=NC(C2=CSC(NC3=CC(NC(C4=CC=C(CN5CCN(C)CC5)S4)=O)=CC=C3C)=N2)=NC=C1	13.5 ± 0.0	A/I<br>3769 ± 2188	14250 ± 1164
**OR0274**	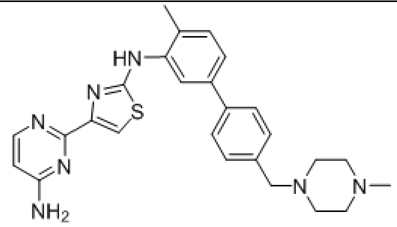	NC1=NC(C2=CSC(NC3=CC(C4=CC=C(CN5CCN(C)CC5)C=C4)=CC=C3C)=N2)=NC=C1	16.7 ± 0.3	A/I<br>1033 ± 10	2096 ± 554
**OR0289**	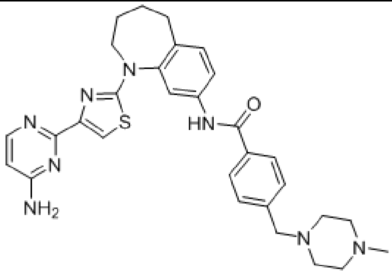	NC1=NC(C2=CSC(N4C3=CC(NC(C5=CC=C(CN6CCN(C)CC6)C=C5)=O)=CC=C3CCCC4)=N2)=NC=C1	15.9 ± 0.5	A/I<br>3405 ± 564	3919 ± 461
**OR0320**	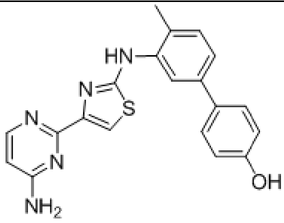	OC(C=C4)=CC=C4C1=CC=C(C)C(NC2=NC(C3=NC=CC(N)=N3)=CS2)=C1	15.0 ± 0.2	A/I<br>1760 ± 385	6772 ± 2812
**OR0321**	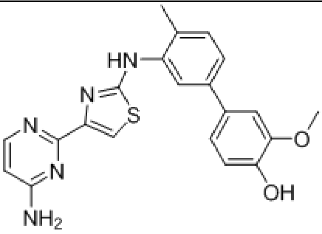	OC(C(OC)=C4)=CC=C4C1=CC=C(C)C(NC2=NC(C3=NC=CC(N)=N3)=CS2)=C1	13.3 ± 0.2	A/I<br>2272 ± 293	8289 ± 2714
**OR0325**	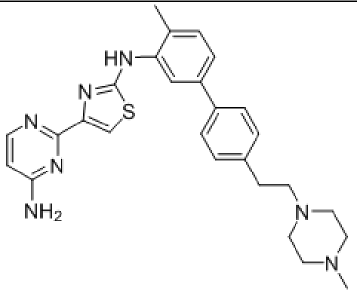	NC1=NC(C2=CSC(NC3=CC(C4=CC=C(CCN5CCN(C)CC5)C=C4)=CC=C3C)=N2)=NC=C1	17.9 ± 0.3	A/I<br>826 ± 78	1676 ± 242
**OR0331**	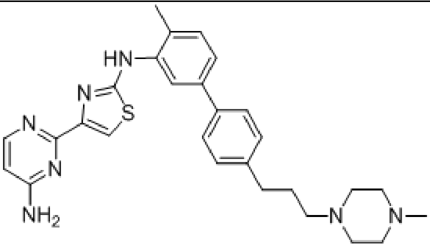	NC1=NC(C2=CSC(NC3=CC(C4=CC=C(CCCN5CCN(C)CC5)C=C4)=CC=C3C)=N2)=NC=C1	19.4 ± 0.4	A/I<br>401 ± 28	918 ± 107

**OR0345**	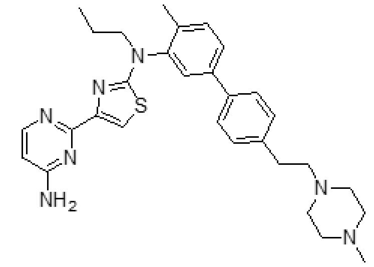	CCCN(C3=CC(C4=CC=C(CCN5CCN(C)CC5)C=C4)=CC=C3C)C1=NC(C2=NC=CC(N)=N2)=CS1	18.4 ± 0.2	I<br>315 ± 50	505 ± 149
**OR0402**	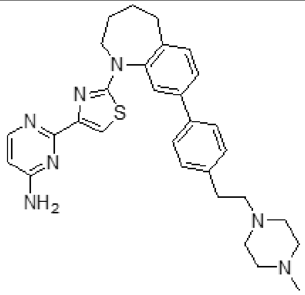	NC1=NC(C2=CSC(N4C3=CC(C5=CC=C(CCN6CCN(C)CC6)C=C5)=CC=C3CCCC4)=N2)=NC=C1	19.2 ± 0.3		1765 ± 160
**OR0596**	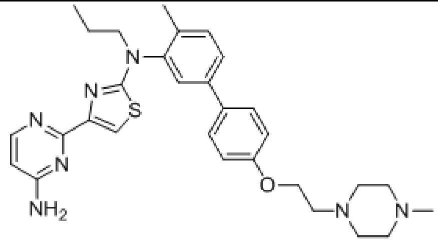	CCCN(C3=CC(C4=CC=C(OCCN5CCN(C)CC5)C=C4)=CC=C3C)C1=NC(C2=NC=CC(N)=N2)=CS1	18.9 ± 0.1	I<br>286 ± 145	307 ± 50
**OR0597**	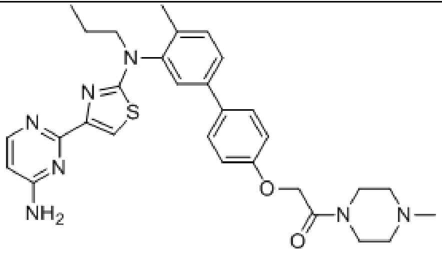	CCCN(C3=CC(C4=CC=C(OCC(N5CCN(C)CC5)=O)C=C4)=CC=C3C)C1=NC(C2=NC=CC(N)=N2)=CS1	17.1 ± 0.1	I<br>349 ± 89	220 ± 127
**OR0598**	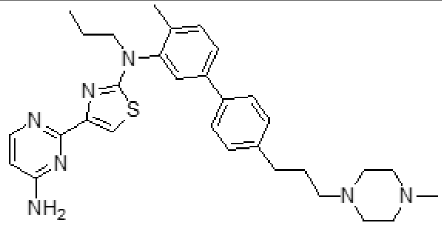	CCCN(C3=CC(C4=CC=C(CCCN5CCN(C)CC5)C=C4)=CC=C3C)C1=NC(C2=NC=CC(N)=N2)=CS1	19.1 ± 0.2	I<br>301 ± 78	776 ± 206
**OR0599**	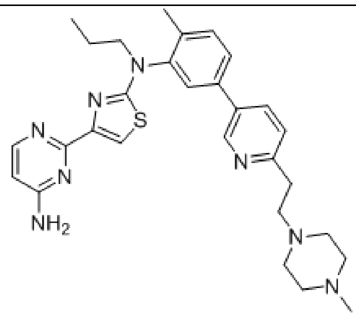	CCCN(C3=CC(C4=CN=C(CCN5CCN(C)CC5)C=C4)=CC=C3C)C1=NC(C2=NC=CC(N)=N2)=CS1	17.1 ± 0.2	I<br>211 ± 52	136 ± 53
**OR0600**	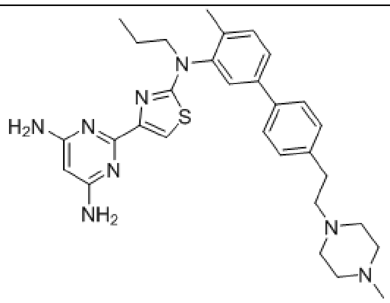	CCCN(C3=CC(C4=CC=C(CCN5CCN(C)CC5)C=C4)=CC=C3C)C1=NC(C2=NC(N)=CC(N)=N2)=CS1	20.0 ± 0.6	I<br>121 ± 21	341 ± 133

**OR0601**	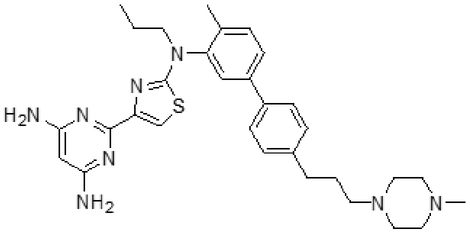	CCCN(C3=CC(C4=CC=C(CCCN5CCN(C)CC5)C=C4)=CC=C3C)C1=NC(C2=NC(N)=CC(N)=N2)=CS1	20.8 ± 0.7	I<br>137 ± 3	420 ± 83
**OR0602**	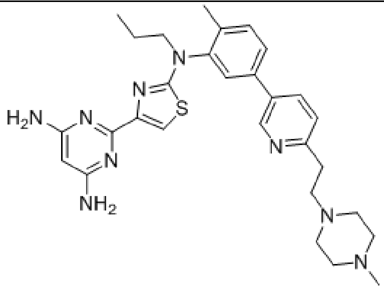	CCCN(C3=CC(C4=CN=C(CCN5CCN(C)CC5)C=C4)=CC=C3C)C1=NC(C2=NC(N)=CC(N)=N2)=CS1	19.5 ± 0.5	I<br>77 ± 14 [A]	130 ± 57
**OR0603**	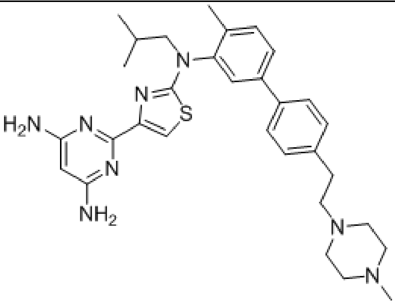	CC(C)CN(C3=CC(C4=CC=C(CCN5CCN(C)CC5)C=C4)=CC=C3C)C1=NC(C2=NC(N)=CC(N)=N2)=CS1	21.3 ± 0.8	I<br>149 ± 44 [A]	255 ± 70
**OR0604**	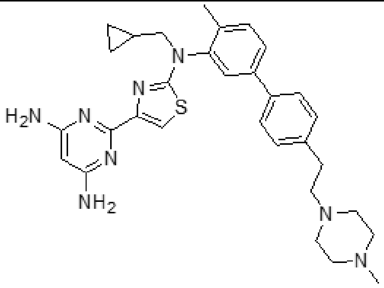	NC1=NC(C2=CSC(N(C4=CC(C5=CC=C(CCN6CCN(C)CC6)C=C5)=CC=C4C)CC3CC3)=N2)=NC(N)=C1	19.7 ± 1.0		609 ± 8
**OR0605**	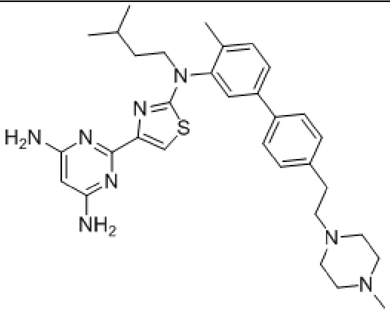	NC1=NC(C2=CSC(N(C3=CC(C4=CC=C(CCN5CCN(C)CC5)C=C4)=CC=C3C)CCC(C)C)=N2)=NC(N)=C1	18.7 ± 0.6		2222 ± 304
**OR0606**	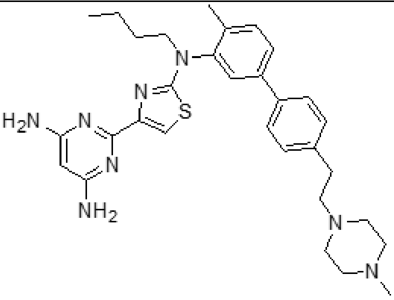	NC1=NC(C2=CSC(N(C3=CC(C4=CC=C(CCN5CCN(C)CC5)C=C4)=CC=C3C)CCCC)=N2)=NC(N)=C1	19.9 ± 0.5		552 ± 102

**OR0608**	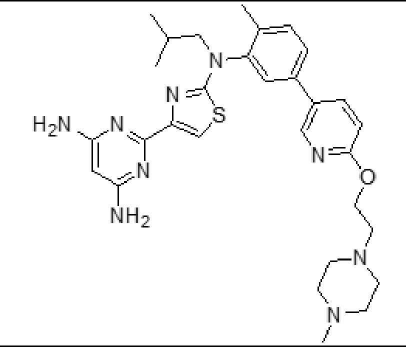	CC(C)CN(C3=CC(C4=CN=C(OCCN5CCN(C)CC5)C=C4)=CC=C3C)C1=NC(C2=NC(N)=CC(N)=N2)=CS1	21.0 ± 0.5	I<br>106 ± 23 [B]	40 ± 13
**OR0609**	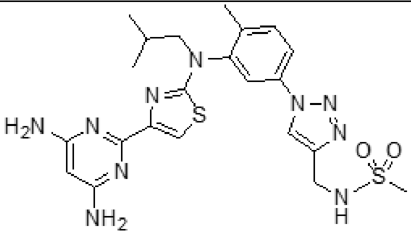	CC(C)CN(C3=CC(N4C=C(CNS(C)(=O)=O)[N][N]4)=CC=C3C)C1=NC(C2=NC(N)=CC(N)=N2)=CS1	13.5 ± 0.7		3177 ± 611
**OR0610**	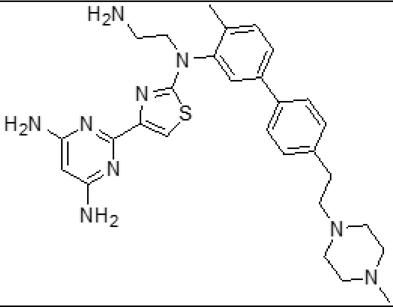	NCCN(C3=CC(C4=CC=C(CCN5CCN(C)CC5)C=C4)=CC=C3C)C1=NC(C2=NC(N)=CC(N)=N2)=CS1	14.7 ± 0.2		16411 ± 4375
**OR0611**	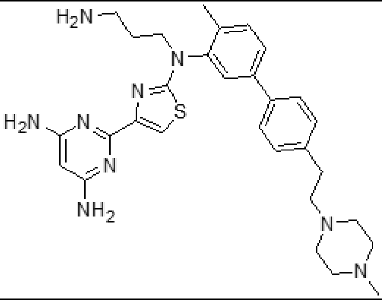	CN(CC5)CCN5CCC(C=C4)=CC=C4C1=CC=C(C)C(N(CCCN)C2=NC(C3=NC(N)=CC(N)=N3)=CS2)=C1	14.7 ± 0.5	A/I<br>498 ± 119 [A]	11804 ± 100
**OR0612**	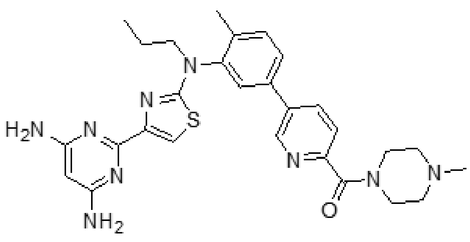	NC1=CC(N)=NC(C2=CSC(N(CCC)C3=CC(C4=CN=C(C(N5CCN(C)CC5)=O)C=C4)=CC=C3C)=N2)=N1	16.6 ± 0.8	I<br>195 ± 16 [A]	1005 ± 300
**OR0613**	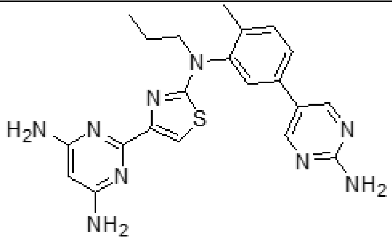	CCCN(C3=CC(C4=CN=C(N)N=C4)=CC=C3C)C1=NC(C2=NC(N)=CC(N)=N2)=CS1	14.5 ± 0.7	I	703 ± 54

**OR0614**	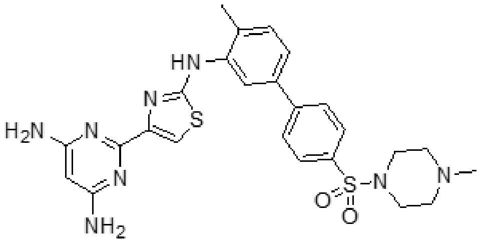	NC1=NC(C2=CSC(NC3=CC(C4=CC=C(S(N5CCN(C)CC5)(=O)=O)C=C4)=CC=C3C)=N2)=NC(N)=C1	18.1 ± 0.8	A/I<br>233 ± 83 [A]	10028 ± 55
**OR0615**	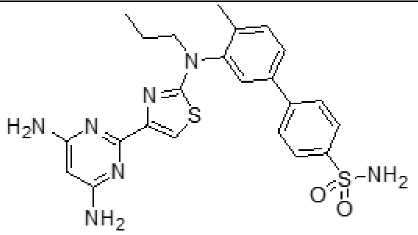	CCCN(C3=CC(C4=CC=C(S(N)(=O)=O)C=C4)=CC=C3C)C1=NC(C2=NC(N)=CC(N)=N2)=CS1	17.5 ± 0.7	I<br>88 ± 68 [A]	411 ± 151
**OR0616**	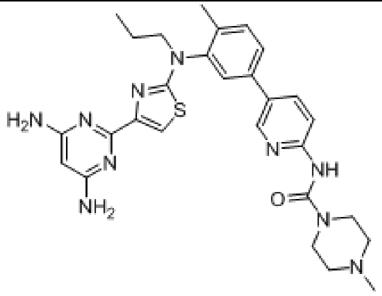	NC1=CC(N)=NC(C2=CSC(N(CCC)C3=CC(C4=CN=C(NC(N5CCN(C)CC5)=O)C=C4)=CC=C3C)=N2)=N1	19.2 ± 0.8		135 ± 51
**OR0617**	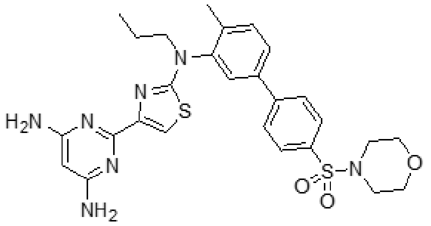	CCCN(C3=CC(C4=CC=C(S(N5CCOCC5)(=O)=O)C=C4)=CC=C3C)C1=NC(C2=NC(N)=CC(N)=N2)=CS1	18.3 ± 1.1	I<br>181 ± 15 [A]	78 ± 45
**OR0618**	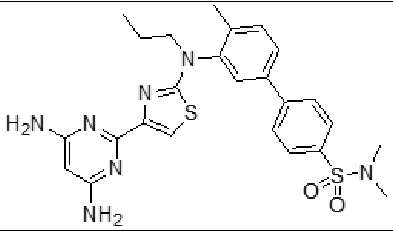	CCCN(C3=CC(C4=CC=C(S(N(C)C)(=O)=O)C=C4)=CC=C3C)C1=NC(C2=NC(N)=CC(N)=N2)=CS1	18.8 ± 0.6	I<br>139 ± 12 [A]	80 ± 37
**OR0619**	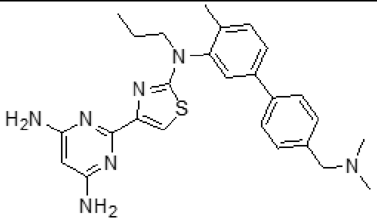	CCCN(C3=CC(C4=CC=C(CN(C)C)C=C4)=CC=C3C)C1=NC(C2=NC(N)=CC(N)=N2)=CS1	16.9 ± 0.5		614 ± 93
**OR0620**	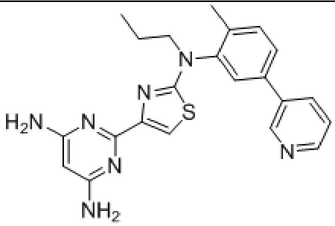	CCCN(C3=CC(C4=CN=CC=C4)=CC=C3C)C1=NC(C2=NC(N)=CC(N)=N2)=CS1	15.7 ± 0.5		407 ± 25

**OR0621**	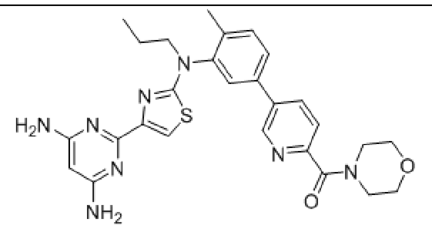	NC1=CC(N)=NC(C2=CSC(N(CCC)C3=CC(C4=CN=C(C(N5CCOCC5)=O)C=C4)=CC=C3C)=N2)=N1	16.6 ± 0.9		169 ± 48
**OR0622**	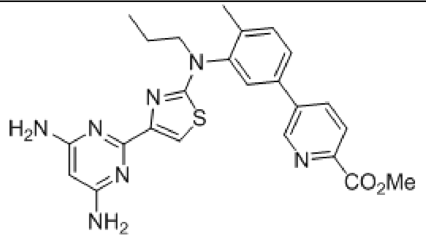	NC1=CC(N)=NC(C2=CSC(N(CCC)C3=CC(C4=CN=C(C(OC)=O)C=C4)=CC=C3C)=N2)=N1	16.5 ± 0.1		416 ± 194
**OR0624**	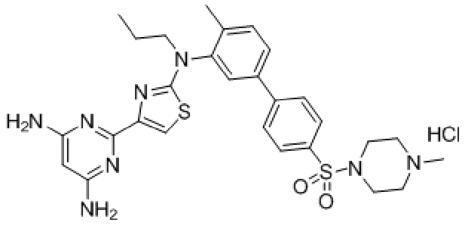	NC1=NC(C2=CSC(N(CCC)C3=CC(C4=CC=C(S(N5CCN(C)CC5)(=O)=O)C=C4)=CC=C3C)=N2)=NC(N)=C1.Cl	19.5 ± 0.8	I<br>198 ± 131 [A]	46 ± 25
**OR0625**	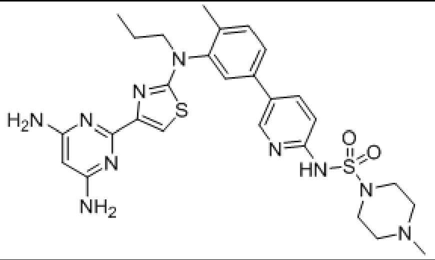	NC1=CC(N)=NC(C2=CSC(N(CCC)C3=CC(C4=CN=C(NS(N5CCN(C)CC5)(=O)=O)C=C4)=CC=C3C)=N2)=N1	16.8 ± 0.7		353 ± 121
**OR0626**	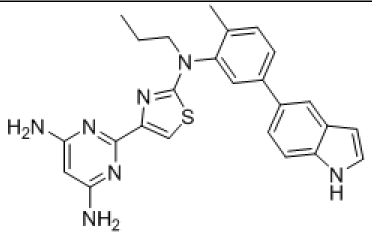	CCCN(C3=CC(C4=CC=C(NC=C5)C5=C4)=CC=C3C)C1=NC(C2=NC(N)=CC(N)=N2)=CS1	16.9 ± 0.5		5750 ± 1276
**OR0629**	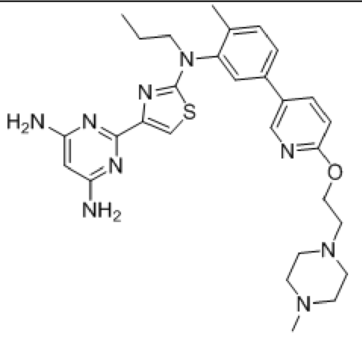	CCCN(C3=CC(C4=CN=C(OCCN5CCN(C)CC5)C=C4)=CC=C3C)C1=NC(C2=NC(N)=CC(N)=N2)=CS1	20.6 ± 0.1		139 ± 77
**OR0630**	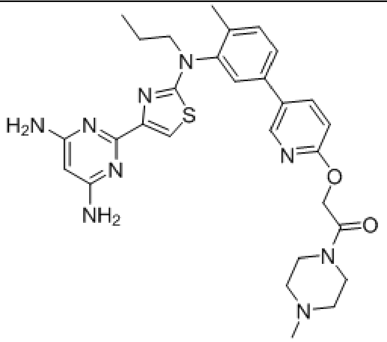	CCCN(C3=CC(C4=CN=C(OCC(N5CCN(C)CC5)=O)C=C4)=CC=C3C)C1=NC(C2=NC(N)=CC(N)=N2)=CS1	17.4 ± 0.2		245 ± 171

**OR0631**	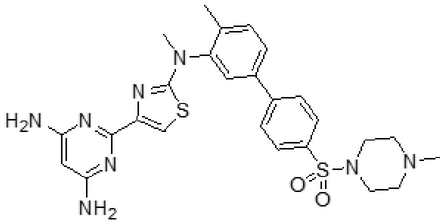	CN(C3=CC(C4=CC=C(S(N5CCN(C)CC5)(=O)=O)C=C4)=CC=C3C)C1=NC(C2=NC(N)=CC(N)=N2)=CS1	16.8 ± 0.1		2740 ± 1088
**OR0632**	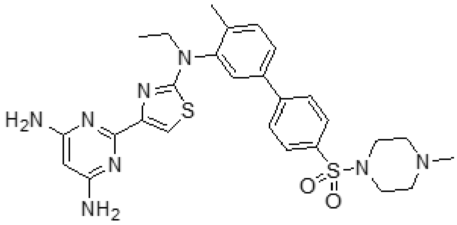	CCN(C3=CC(C4=CC=C(S(N5CCN(C)CC5)(=O)=O)C=C4)=CC=C3C)C1=NC(C2=NC(N)=CC(N)=N2)=CS1	17.8 ± 0.1		413 ± 131
**OR0633**	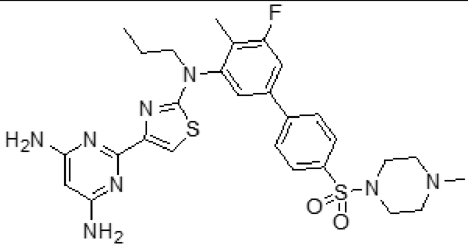	CCCN(C3=CC(C4=CC=C(S(N5CCN(C)CC5)(=O)=O)C=C4)=CC(F)=C3C)C1=NC(C2=NC(N)=CC(N)=N2)=CS1	17.3 ± 0.1		140 ± 57
**OR0634**	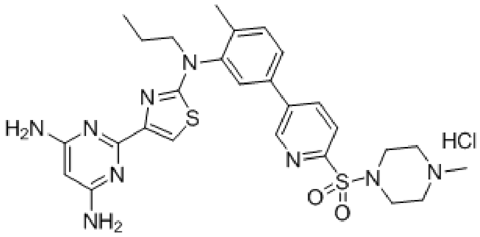	NC1=NC(C2=CSC(N(CCC)C3=CC(C4=CN=C(S(N5CCN(C)CC5)(=O)=O)C=C4)=CC=C3)=N2)=NC(N)=C1.Cl	17.5 ± 0.4	I<br>100 ± 82 [A]	54 ± 29
**OR0635**	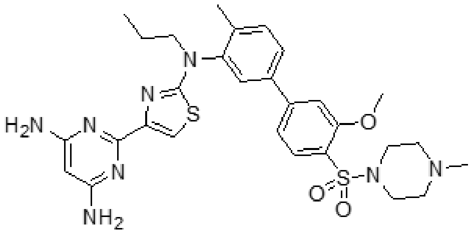	CCCN(C3=CC(C4=CC=C(S(N5CCN(C)CC5)(=O)=O)C(OC)=C4)=CC=C3C)C1=NC(C2=NC(N)=CC(N)=N2)=CS1	20.40 ± 0.9	I<br>66 ± 11 [A]	15.4 ± 10
**OR0636**	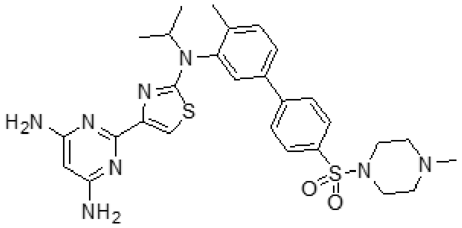	CC(C)N(C3=CC(C4=CC=C(S(N5CCN(C)CC5)(=O)=O)C=C4)=CC=C3C)C1=NC(C2=NC(N)=CC(N)=N2)=CS1	17.7 ± 0.9		301 ± 116
**OR0637**	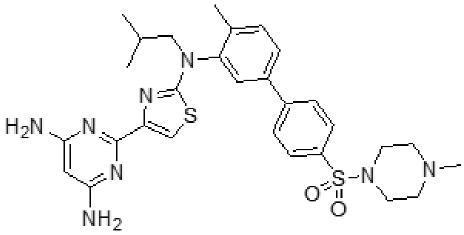	CC(C)CN(C3=CC(C4=CC=C(S(N5CCN(C)CC5)(=O)=O)C=C4)=CC=C3C)C1=NC(C2=NC(N)=CC(N)=N2)=CS1	20.5 ± 0.9		84 ± 49

**OR0638**	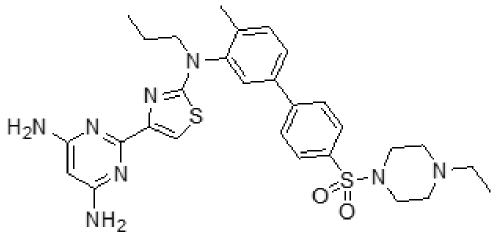	CCCN(C3=CC(C4=CC=C(S(N5CCN(CC)CC5)(=O)=O)C=C4)=CC=C3C)C1=NC(C2=NC(N)=CC(N)=N2)=CS1	20.2 ± 0.9		35 ± 17
**OR0639**	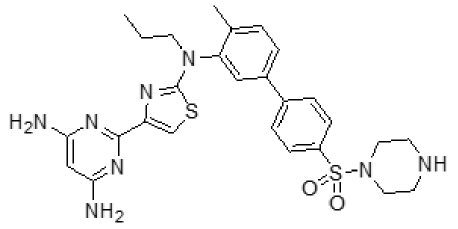	CCCN(C3=CC(C4=CC=C(S(N5CCNCC5)(=O)=O)C=C4)=CC=C3C)C1=NC(C2=NC(N)=CC(N)=N2)=CS1	20.7 ± 0.9		119 ± 36
**OR0640**	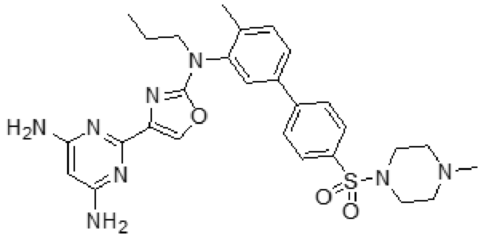	CCCN(C3=CC(C4=CC=C(S(N5CCN(C)CC5)(=O)=O)C=C4)=CC=C3C)C1=NC(C2=NC(N)=CC(N)=N2)=CO1	18.2 ± 0.9		3670 ± 2151
**OR0641**	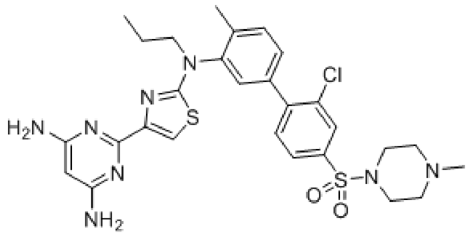	CCCN(C3=CC(C4=CC=C(S(N5CCN(C)CC5)(=O)=O)C=C4Cl)=CC=C3C)C1=NC(C2=NC(N)=CC(N)=N2)=CS1	21.1 ± 1.1		10.5 ± 5.0
**OR0642**	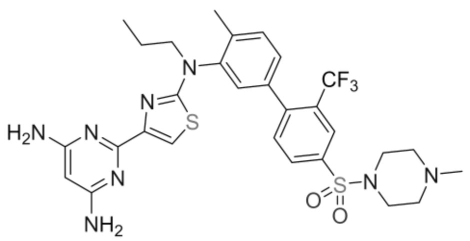	CCCN(C3=CC(C4=CC=C(S(N5CCN(C)CC5)(=O)=O)C=C4C(F)(F)F)=CC=C3C)C1=NC(C2=NC(N)=CC(N)=N2)=CS1	22.2 ± 0.9	I<br>106 ± 30 [A]	2.2 ± 2.0
**OR0643**	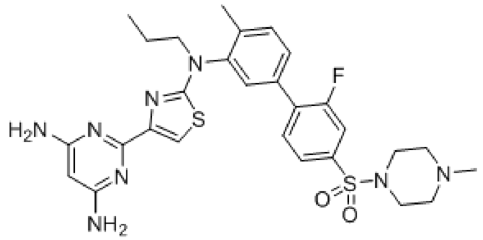	CCCN(C3=CC(C4=CC=C(S(N5CCN(C)CC5)(=O)=O)C=C4F)=CC=C3C)C1=NC(C2=NC(N)=CC(N)=N2)=CS1	20.2 ± 0.9		46 ± 18
**OR0644**	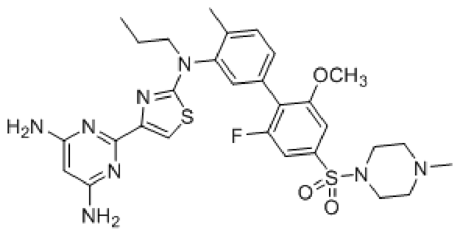	CCCN(C3=CC(C4=C(F)C=C(S(N5CCN(C)CC5)(=O)=O)C=C4OC)=CC=C3C)C1=NC(C2=NC(N)=CC(N)=N2)=CS1	20.5 ± 1.2		12.1 ± 7.7

**OR0645**	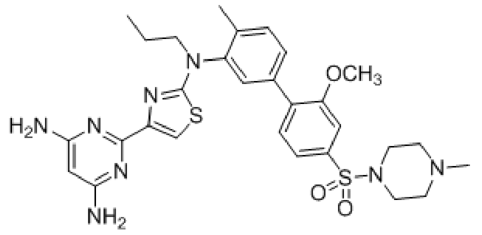	CCCN(C3=CC(C4=CC=C(S(N5CCN(C)CC5)(=O)=O)C=C4OC)=CC=C3C)C1=NC(C2=NC(N)=CC(N)=N2)=CS1	19.7 ± 0.9		67 ± 22
**OR0646**	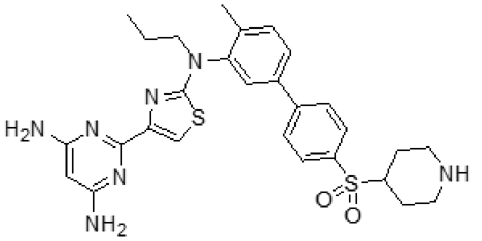	CCCN(C3=CC(C4=CC=C(S(C5CCNCC5)(=O)=O)C=C4)=CC=C3C)C1=NC(C2=NC(N)=CC(N)=N2)=CS1	22.4 ± 1.1		171 ± 48
**OR0647**	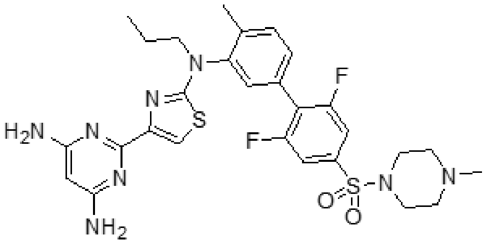	CCCN(C3=CC(C4=C(F)C=C(S(N5CCN(C)CC5)(=O)=O)C=C4F)=CC=C3C)C1=NC(C2=NC(N)=CC(N)=N2)=CS1	21.1 ± 1.6		10.9 ± 4.1
**OR0648**	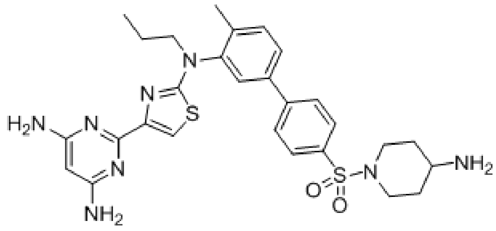	CCCN(C3=CC(C4=CC=C(S(N5CCC(N)CC5)(=O)=O)C=C4)=CC=C3C)C1=NC(C2=NC(N)=CC(N)=N2)=CS1	22.3 ± 1.1		134 ± 38
**OR0649**	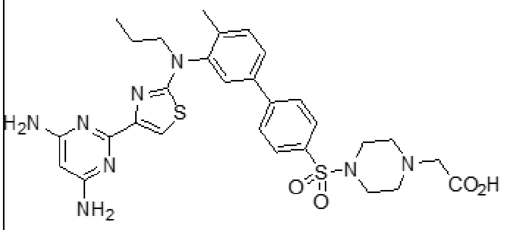	CCCN(C3=CC(C4=CC=C(S(N5CCN(CC(O[Na])=O)CC5)(=O)=O)C=C4)=CC=C3C)C1=NC(C2=NC(N)=CC(N)=N2)=CS1	17.6 ± 1.3		13417 ± 546
**OR0650**	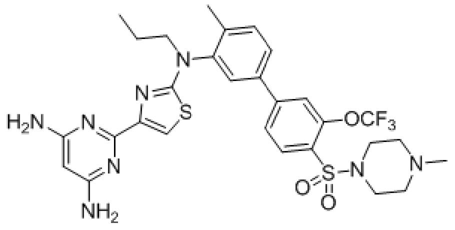	CCCN(C3=CC(C4=CC=C(S(N5CCN(C)CC5)(=O)=O)C(OC(F)(F)F)=C4)=CC=C3C)C1=NC(C2=NC(N)=CC(N)=N2)=CS1	18.6 ± 1.5		120 ± 25
**OR0651**	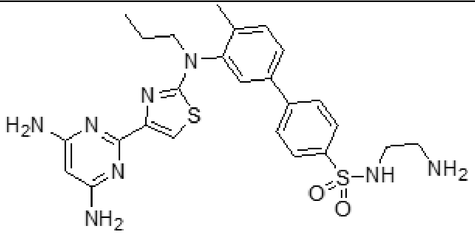	CCCN(C3=CC(C4=CC=C(S(NCCN)(=O)=O)C=C4)=CC=C3C)C1=NC(C2=NC(N)=CC(N)=N2)=CS1	20.8 ± 1.2		1525 ± 178

**OR0652**	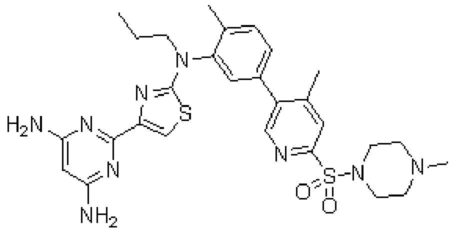	CCCN(C3=CC(C4=CN=C(S(N5CCN(C)CC5)(=O)=O)C=C4C)=CC=C3C)C1=NC(C2=NC(N)=CC(N)=N2)=CS1	19.2 ± 0.1		7.3 ± 2.5

Supplementary Table 2. Results of the acute toxicity studies and plasma pharmacokinetic parameters.

COMPOUND	ACUTE TOXICITY STUDIES	PLASMA PHARMACOKINETIC PARAMETERS			
	Maximum tolerated dose (mg/Kg)	Dose (mg/Kg)	C_{max} (nmol/mL)	T_{max} (h)	T ½ (min)
**OR0602**	10	10	0.96 ± 0.04	1	120 ± 14
**OR0624**	20	20	3.1 ± 0.6	1	133 ± 11
**OR0634**	> 80	40	5.5 ± 1.3	1	129 ± 17
**OR0635**	> 40	40	2.0 ± 0.2	1.5	373 ± 203
**OR0642**	> 80	20	0.8 ± 0.2	2	7 ± 3 (h)
		40	4.1 ± 1.5	5	> 8 (h)

Supplementary Table 3. KINOMEscan™ Profiling of OR0642. Competition binding assay to quantitatively measure interactions between OR0642 (1 µM) and 97 human kinases and disease relevant mutant variants.

Target Gene Symbol	%Control (OR0642 1 µM)	Target Gene Symbol	%Control (OR0642 1 µM)
ABL1(E255K)-phosphorylated	96	LKB1	99
ABL1(T315I)-phosphorylated	89	MAP3K4	97
ABL1-nonphosphorylated	94	MAPKAPK2	100
ABL1-phosphorylated	99	MARK3	100
ACVR1B	86	MEK1	97
ADCK3	95	MEK2	96
AKT1	91	MET	100
AKT2	100	MKNK1	100
ALK	80	MKNK2	79
AURKA	96	MLK1	91
AURKB	85	p38-alpha	100
AXL	89	p38-beta	91
BMPR2	94	PAK1	100
BRAF	99	PAK2	98
BRAF(V600E)	98	PAK4	91
BTK	92	PCTK1	98
CDK11	100	PDGFRA	97
CDK2	100	PDGFRB	100
CDK3	97	PDPK1	98
CDK7	94	PIK3C2B	100
CDK9	98	PIK3CA	100
CHEK1	91	PIK3CG	100
CSF1R	100	PIM1	100
CSNK1D	100	PIM2	100
CSNK1G2	91	PIM3	100
DCAMKL1	81	PKAC-alpha	85
DYRK1B	100	PLK1	90
EGFR	82	PLK3	100
EGFR(L858R)	72	PLK4	81
EPHA2	90	PRKCE	100
ERBB2	100	RAF1	88
ERBB4	100	RET	100
ERK1	86	RIOK2	98
FAK	100	ROCK2	99
FGFR2	89	RSK2(Kin.Dom.1-N-terminal)	100
FGFR3	79	SNARK	88
FLT3	100	SRC	92
GSK3B	100	SRPK3	93
IGF1R	93	TGFBR1	98
IKK-alpha	65	TIE2	94
IKK-beta	90	TRKA	100
INSR	99	TSSK1B	100
JAK2(JH1domain-catalytic)	100	TYK2(JH1domain-catalytic)	100
JAK3(JH1domain-catalytic)	97	ULK2	95
JNK1	93	VEGFR2	100
JNK2	84	YANK3	97
JNK3	81	ZAP70	97
KIT	91	YANK3	97
KIT(D816V)	94	ZAP70	97
KIT(V559D,T670I)	100		

Supplementary Table 4. Data collection and refinement statistics of crystal structures of dCK in complex with dCKi1, dCKi2, OR0642, OR0274, and OR0325. Statistics for the highest-resolution shell are shown in parentheses. A fraction of reflections (5%) were set aside for calculating R_{free} during the refinement process.

	**dCK-C4S-S74E in complex with dCKi1**	**dCK-C4S-S74E in complex with dCKi2**	**dCK-C4S-S74E in complex with OR0642**	**dCK-C4S-S74E in complex with OR0274**	**dCK-C4S-S74E in complex with OR0325**
**PDB ID**	7ZI1	7ZI2	7ZI3	7ZI5	7ZI6
**Data collection**					
X-ray source	ESRF	ESRF	SOLEIL	SOLEIL	SOLEIL
Beamline	ID30B	ID30B	Proxima 1	Proxima 2	Proxima 2
Space group	P 41 2 2	P 41 2 2	P 41 2 2	P 41 2 2	P 41 2 2
Cell dimensions:					
a, *b*, *c* (Å)	68.62, 68.62, 120.81	68.84, 68.84, 123.66	68.94, 68.94, 120.76	68.75, 68.75, 123.09	68.63, 68.63, 122.72
A, β, γ (°)	90,90,90	90,90,90	90,90,90	90,90,90	90,90,90
Resolution (Å)	1.85 (1.85 - 1.88)	2.18 (2.18 - 2.22)	1.90 (1.90 - 2.02)	2.00 (2.00 - 2.12)	2.10 (2.10 - 2.23)
Number of reflections:					
Observed	275750 (13817)	174047 (9110)	605892 (99631)	515360 (81180)	451660 (72926)
Unique	25420 (1221)	16221 (810)	23515 (3700)	20613 (3180)	17508 (2727)
Completeness (%)	100 (98.9)	100 (100)	99.9 (98.8)	99.4 (96.7)	98.0 (96.8)
R_{merge} (I)	0.179 (1.958)	0.120 (1.869)	0.085 (2.593)	0.119 (1.657)	0.107 (1.918)
I / σ(I)	6.1 (0.6)	10.1 (1.1)	19.73 (1.12)	14.83 (1.31)	19.65 (1.90)
CC1/2	0.996 (0.558)	0.998 (0.515)	0.999 (0.626)	0.998 (0.777)	0.999 (0.750)
**Structure refinement**					
Resolution range (Å)	48.52 - 1.85	48.72 - 2.18	48.75 - 1.90	48.62 - 2.00	48.53 - 2.10
Number of reflections	25358	16168	23515	20608	17471
R_{factor} / R_{free}	0.201 / 0.234	0.201 / 0.262	0.190 / 0.230	0.193 / 0.228	0.196 / 0.236
Number of atoms:					
Protein	1916	1923	1935	1930	1926
Ligand/UDP	37 x 2 / 25	38 / 25	44 / 25	34 / 25	35 / 25
Water	93	37	65	46	42
B-factors:					
Protein	37.22	56.03	55.14	58.67	59.27
Ligand/UDP	44.73	59.92	54.08	53.94	61.09
Water	36.91	45.18	52.71	51.27	50.03
R.m.s. deviations:					
Bond lengths (Å)	0.0091	0.0076	0.0091	0.0086	0.0083
Bond angles (°)	1.573	1.581	1.601	1.575	1.593
Ramachandran plot (%)					
Favored regions	97.30	95.54	96.43	95.96	96.41
Allowed regions	2.25	4.02	3.12	3.59	3.14
Outliers regions	0.45	0.45	0.45	0.45	0.45

Supplementary Table 5. Data collection and refinement statistics of crystal structures of dCK in complex with OR0345, OR0602, OR0624, OR0634, and OR0635. Statistics for the highest-resolution shell are shown in parentheses. A fraction of reflections (5%) are set aside for calculating R_{free} during the refinement process.

	**dCK-C4S-S74E in complex with OR0345**	**dCK-C4S-S74E in complex with OR0602**	**dCK-C4S-S74E in complex with OR0624**	**dCK-C4S-S74E in complex with OR0634**	**dCK-C4S-S74E in complex with OR0635**
**PDB ID**	7ZI7	7ZI8	7ZI9	7ZIA	7ZIB
**Data collection**					
X-ray source	SOLEIL	SOLEIL	SOLEIL	SOLEIL	ESRF
Beamline	Proxima 1	Proxima 2	Proxima 2	Proxima 2	ID30A1
Space group	P 41 2 2	P 41 2 2	P 41 2 2	P 41 2 2	P 41 2 2
Cell dimensions:					
a, *b*, *c* (Å)	68.63, 68.63, 120.60	68.62, 68.62, 121.25	69.05, 69.05, 121.64	69.00, 69.00, 121.17	68.51, 68.51, 120.33
Α, β, γ (°)	90,90,90	90,90,90	90,90,90	90,90,90	90,90,90
Resolution (Å)	1.80 (1.80 - 1.91)	1.99 (1.99 – 2.11)	1.80 (1.80 – 1.91)	1.70 (1.70 – 1.80)	1.95 (1.95 – 2.05)
Number of reflections:					
Observed	730806 (117192)	521226 (78501)	705343 (94510)	825173 (109067)	207825 (32362)
Unique	27338 (4315)	20446 (3201)	28088 (4364)	32937 (5158)	22140 (3493)
Completeness (%)	100 (100)	99.9 (99.3)	99.6 (97.6)	99.8 (98.8)	99.8 (99.7)
R_{merge} (*I*)	0.067 (1.372)	0.118 (3.387)	0.166 (2.368)	0.057 (1.789)	0.077 (1.277)
$I / \sigma(I)$	26.10 (2.24)	18.99 (0.87)	17.62 (1.12)	28.58 (1.47)	16.12 (1.50)
CC1/2	0.999 (0.834)	0.999 (0.410)	0.999 (0.604)	0.100 (0.691)	0.999 (0.565)
**Structure refinement**					
Resolution range (Å)	48.53 - 1.80	48.52 - 1.99	48.83 - 1.80	48.79 - 1.70	45.21 - 1.95
Number of reflections	27338	20446	28088	32936	21580
R_{factor} / R_{free}	0.187 / 0.227	0.201 / 0.263	0.184 / 0.220	0.187 / 0.222	0.178 / 0.232
No. atoms:					
Protein	1931	1921	1933	1937	1914
Ligand/UDP	38 / 25	39 / 25	40 / 25	40 / 25	42 / 25
Water	88	32	118	99	70
B-factors:					
Protein	46.09	58.36	38.58	42.84	44.41
Ligand/UDP	54.93	61.02	39.97	49.32	51.08
Water	46.25	51.8	40.93	44.99	42.98
R.m.s. deviations:					
Bond lengths (Å)	0.0106	0.0084	0.0103	0.0118	0.0107
Bond angles (°)	1.658	1.533	1.657	1.778	1.648
Ramachandran plot (%)					
Favored regions	96.88	96.41	96.88	96.43	96.85
Allowed regions	2.68	3.14	2.68	3.12	2.70
Outliers regions	0.45	0.45	0.45	0.45	0.45

Supplementary Table 6. Sequences of human lentiviral DCK sgRNA. Sequences used to establish the stable CCRF-CEM-CRISPR-sgRNA-dCK- cell line (Dharmacon™ Edit-R™ Lentiviral sgRNAs).

Catalog #	Source Clone ID	Gene Symbol	Genomic Location	DNA Target Sequence
GSGH11838	VSGHSM_26724779	DCK	hg38|+chr4:70993910-70993932	CATCGAAGGGAA CATCGGTA
GSGH11838	VSGHSM_26724783	DCK	hg38|-chr4:71022447-71022469	GACTGAGACAGG CATATGTT
GSGH11838	VSGHSM_26724784	DCK	hg38|+chr4:71025901-71025923	ATGAAAGCTGGCT CCTGCAT

Supplementary Table 7. Stability of oncogenetic lesions and the immunophenotype in patient primary vs patient-derived xenograft samples. Bold lesions are conserved between primary sample and matched PDX.

**UPNT**	**Age**	**Sex**	**Leuko-cytosis (G/l)**	**CNS invol-vement**	**Oncogenetics**		**Immunophenotype**	
					**Patient primary sample**	**Patient-derived xenograft**	**Patient primary sample**	**Patient-derived xenograft**
**380**	38	Female	84.79	Negative	**SIL-TAL1-positive, CDKN2A/B deletion**, PTEN mutation (InsT233)	**SIL-TAL1-positive, CDKN2A/B deletion**	TCR Alpha Beta	TCR Alpha Beta
**489**	36	Male	295.5	Positive	**CDKN2A/B deletion, PTEN deletion, NOTCH1 mutation (V1676L), NRAS mutation (G12D),** BCL11B mutation (R447H)	**CDKN2A/B deletion, PTEN deletion, NOTCH1 mutation (V1676L), NRAS mutations (G12D, G13D)**	IMB/preAB	IMB/preAB
**525**	23	Male	604.4	Positive	**CDKN2A/B deletion, NRAS mutation (G13D)**	**CDKN2A/B deletion, NRAS mutation (G13D)**	IM0/G/D	IM0/G/D
**615**	19	Male	26	Negative	**CDKN2A/B deletion, BCL11B mutation (p.AQ424fs), FBXW7 mutation (R465H), NOTCH1 mutation (L1678P), PIK3R1 mutation (p.D578_Q579insDQ)**	**CDKN2A/B deletion, BCL11B mutation (p.AQ424fs), FBXW7 mutation (R465H), NOTCH1 mutation (L1678P), PIK3R1 mutation (p.D578_Q579insDQ)**	TCR Alpha Beta	TCR Alpha Beta
**730**	36	Female	19.9	Negative	**JAK1 mutation (S1043I), JAK3 mutation (G589S, K867E), FBXW7 mutation (R278*),** DNMT3A mutation (R882H)	**JAK1 mutation (S1043I), JAK3 mutation (G589S, K867E), FBXW7 mutation (R278*)**	TCR Gamma Delta	TCR Gamma Delta
**775**	15	Male	620	Negative	**SIL-TAL1, CDKN2A/B deletion, LEF1 deletion, PIK3R1 mutation (K567E),** PTEN deletion, KMT2C mutation (S1742I), RELN mutation (C243R)	**SIL-TAL1, CDKN2A/B deletion, LEF1 deletion, PIK3R1 mutation (K567E)**	IMB/preAB	IMB/preAB

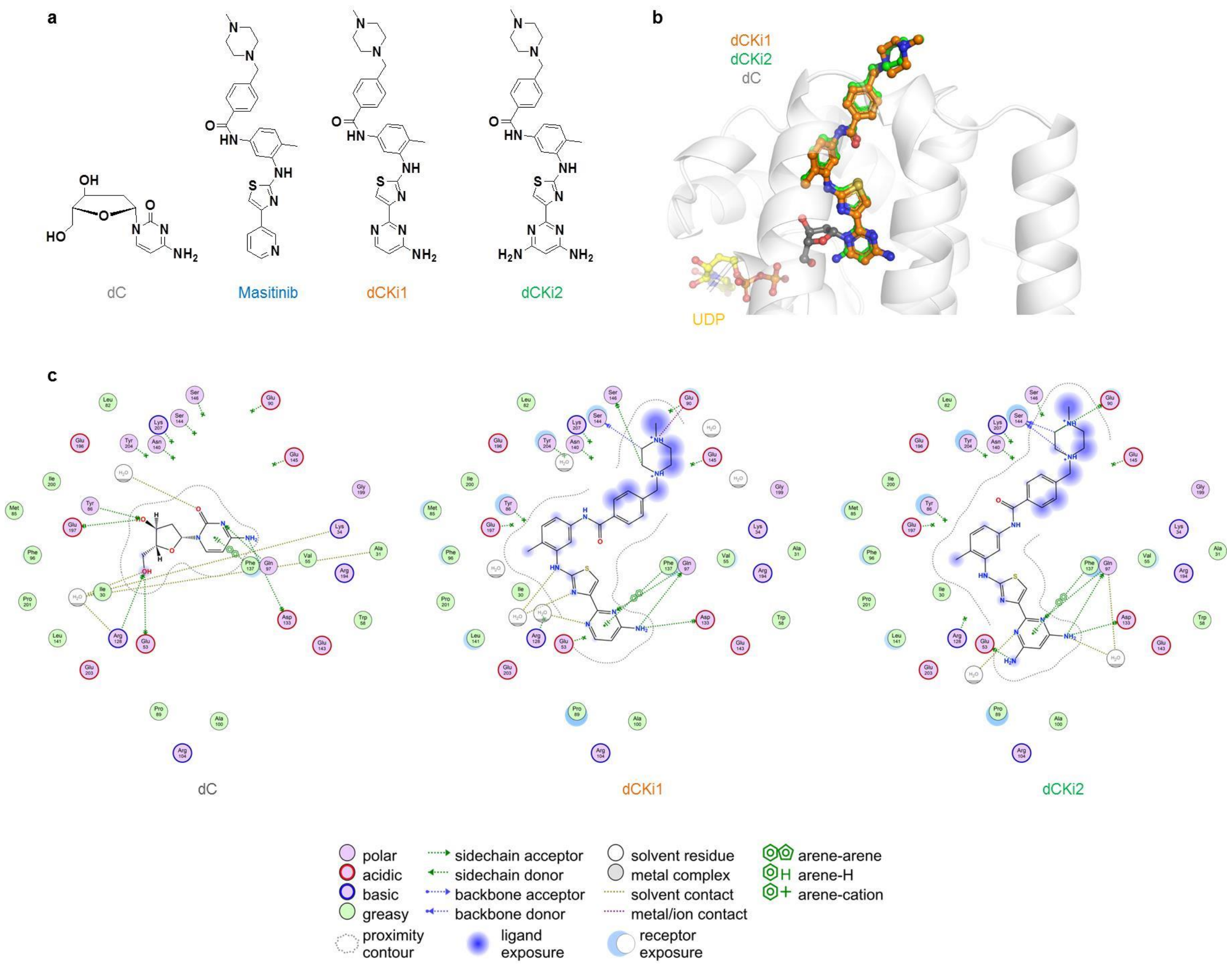

Supplementary Figure 1. Structure of dCK in complex with derivatives dCKi1 (PDB 7ZI1) and dCKi2 (PDB 7ZI2). a Chemical structures of deoxycytidine (dC), Masitinib, dCKi1 and dCKi2. **b** Superimposition of crystal structures of dCK (white ribbons) in complex with UDP (yellow) and dCKi1 (orange) (PDB 7ZI1), dCKi2 (green) (PDB 7ZI2) and dC (grey) (PDB 2NO1) showing the overlap of ring A (dCKi1 and dCKi2) with the pyrimidine ring of dC. **c** Protein-ligand interaction diagrams calculated with the Molecular Operating Environment software (MOE) for dC, dCKi1 and dCKi2 structures.

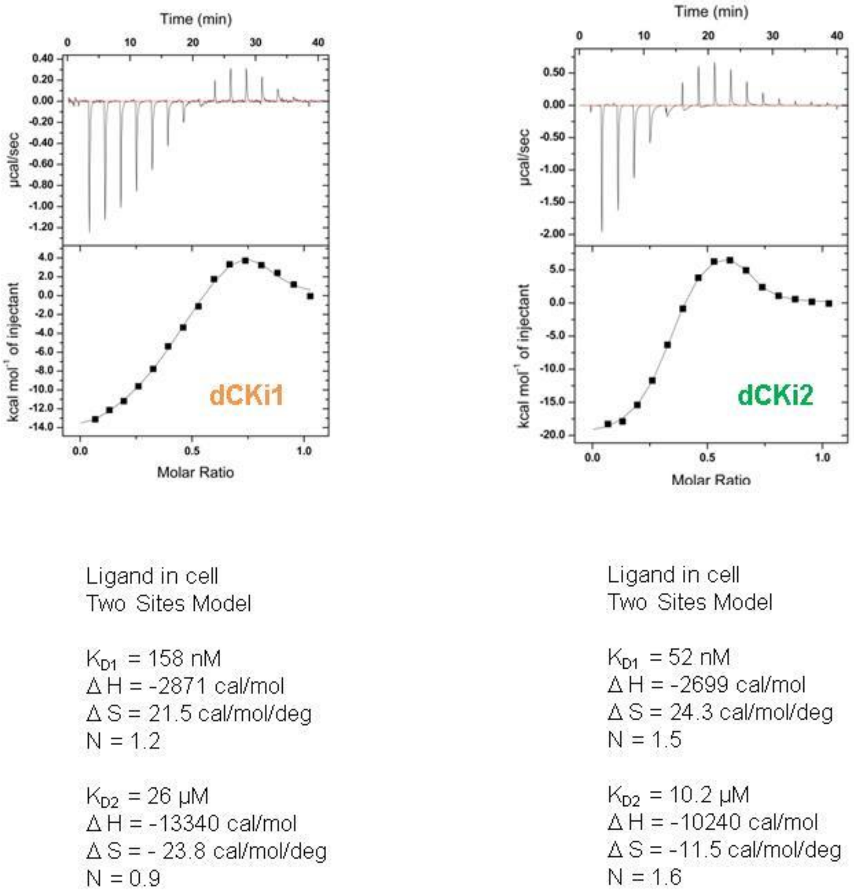

Supplementary Figure 2. Representative calorimetric titrations determined by Isothermal Titration Calorimetry (ITC) corresponding to the interaction of dCK with dCKi1 and dCKi2 at 25°C. The upper panels show the ITC thermograms raw data (thermal power as a function of time) and the lower panel presents the integrated heat for each injection as a function of the molar ratio of ligand to protein. Binding isotherms were fitted to the raw data using two-site binding model because biphasic curves and X-ray structures supported the presence of a second binding site with lower affinity.

a

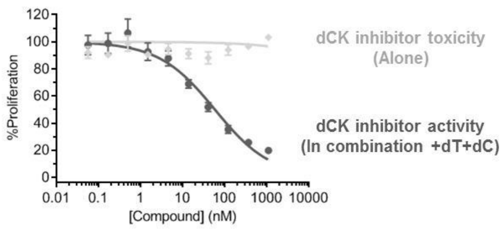

b

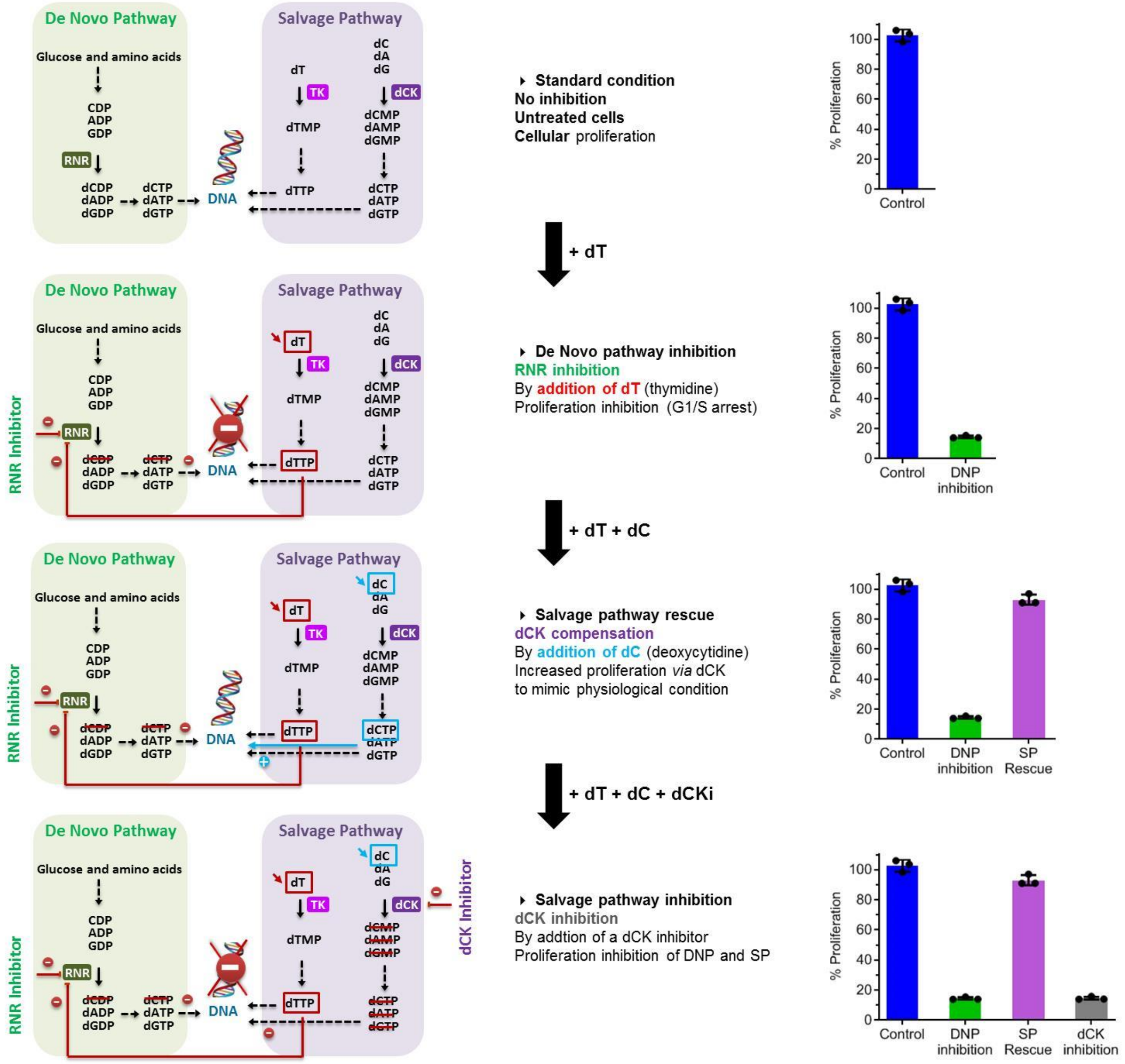

Supplementary Figure 3. dCK cellular assay performed to assess the ability of compounds to inhibit dCK activity in a cellular model. a Representative experiment showing the assay. Cellular assays were performed to assess the inhibition of cell proliferation under two conditions: i) in the presence of the compound alone to determine the non-specific effect on cells, independent of dCK (non-specific toxicity) and ii) in the presence of an inhibitor of the De Novo pathway to determine the ability of compounds to inhibit dCK and decrease tumor proliferation (activity on dCK). **b** Representation of the differential metabolic cellular assay to study DNP and SP inhibitors and validate whether a combinatory inhibition of these pathways by the combination of a dCK inhibitor with a RNR inhibitor could lead to the inhibition of cellular proliferation. The inhibitor of the

De Novo pathway chosen for the experiments was thymidine (dT), a physiological inhibitor of ribonucleotide reductase which arrests cell proliferation in phase S. The dTTP produced via thymidine kinase from dT acts as a RNR inhibitor by an allosteric regulation of the R1 subunit. The addition of the nucleoside deoxycytidine (dC) in the medium allows the rescue of cell proliferation by passing only through the salvage pathway, mediated by dCK. dC needs to be added exogenously to mimic physiological concentrations because it is not present in the cell culture medium, on the contrary to mouse model, in which it is naturally present in the serum. Therefore, cell proliferation under these conditions (+dT +dC) is dCK dependent and it is possible to determine the IC_{50} of dCK inhibitors on the inhibition of cell proliferation. Graphs show a representative experiment as an example (*n* = 3).

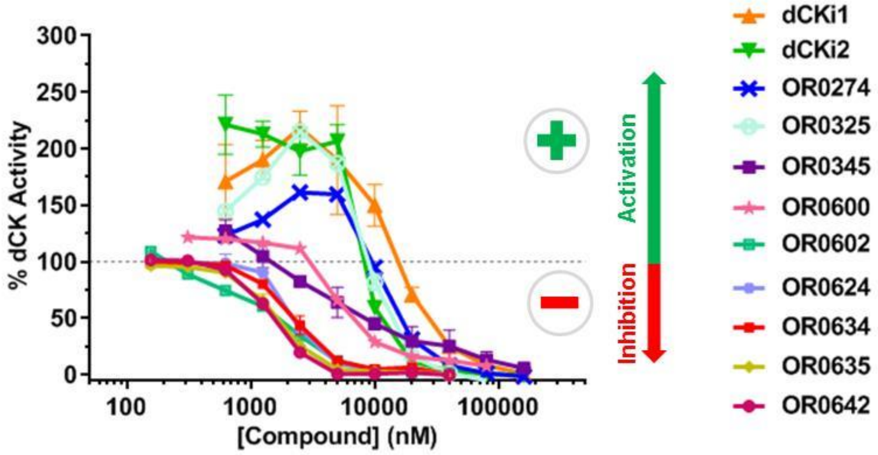

Supplementary Figure 4. Effect of selected compounds on dCK enzymatic activity in the presence of UTP. Representative experiment showing the effect of selected compounds on substrate phosphorylation by dCK in the presence of UTP. Data are presented as the mean of three technical replicates ± SD. Substrate phosphorylation by dCK can be activated or inhibited depending on compound structure. While dCKi1 is an activator at low concentration and inhibitor at high concentration, OR0642 is an inhibitor even at concentrations in the nanomolar range.

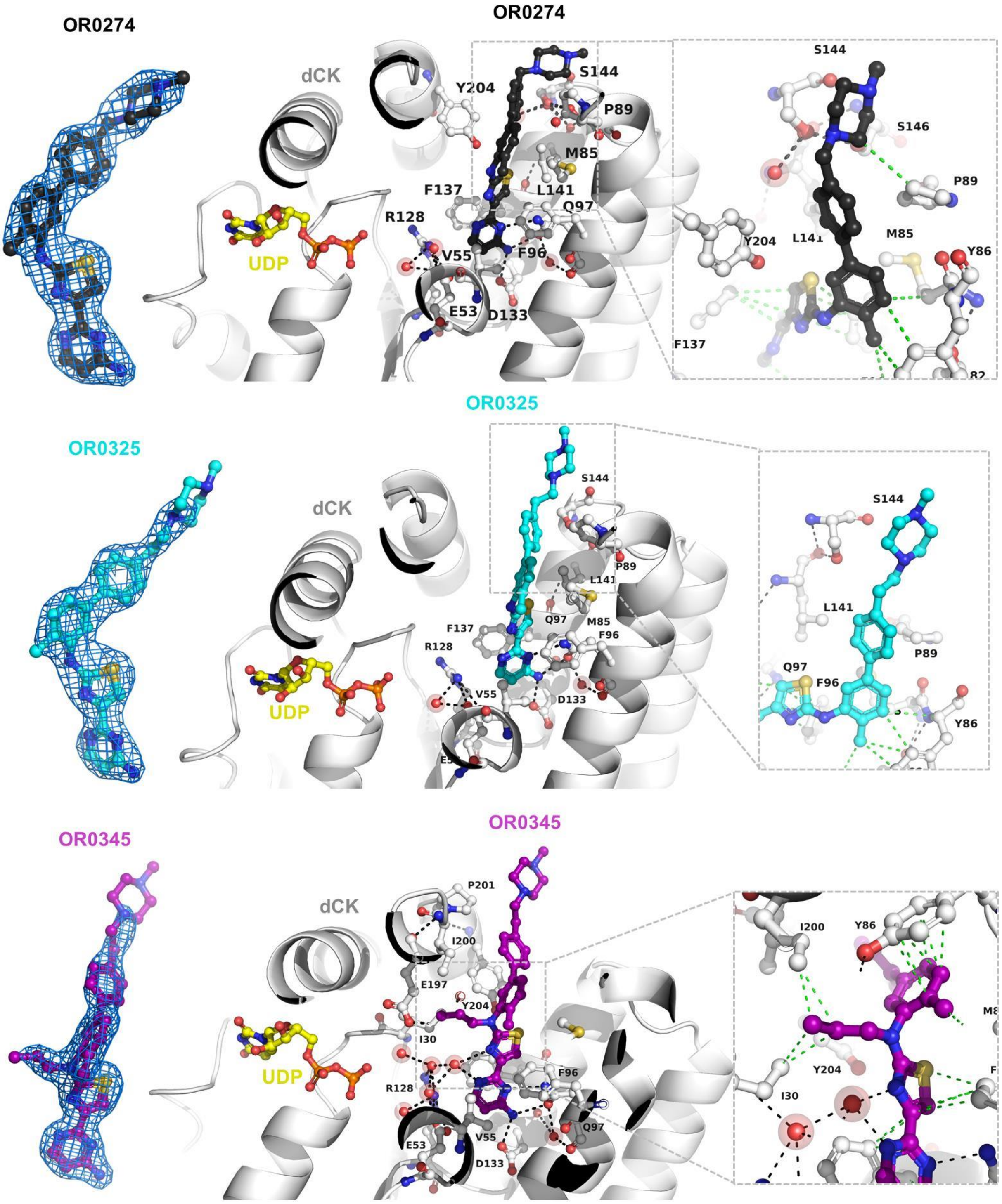

Supplementary Figure 5. Structure of dCK in complex with derivatives OR0274, OR0325, and OR0345. Structure of dCK (grey ribbon) in complex with derivatives OR0274 (PDB 7ZI5), OR0325 (PDB 7ZI6), and OR0345 (PDB 7ZI7), and UDP (yellow), showing the hydrogen bond network to dCK (black dashed lines). The meshes around the compound (left) shows the |2Fo|−|Fc| electron density map contoured at 2.0σ around the ligand (blue). Hydrogen bonds between compounds and dCK are shown as dashed lines in the zoom inserts (right) and vdW interactions are shown as green dashed lines.

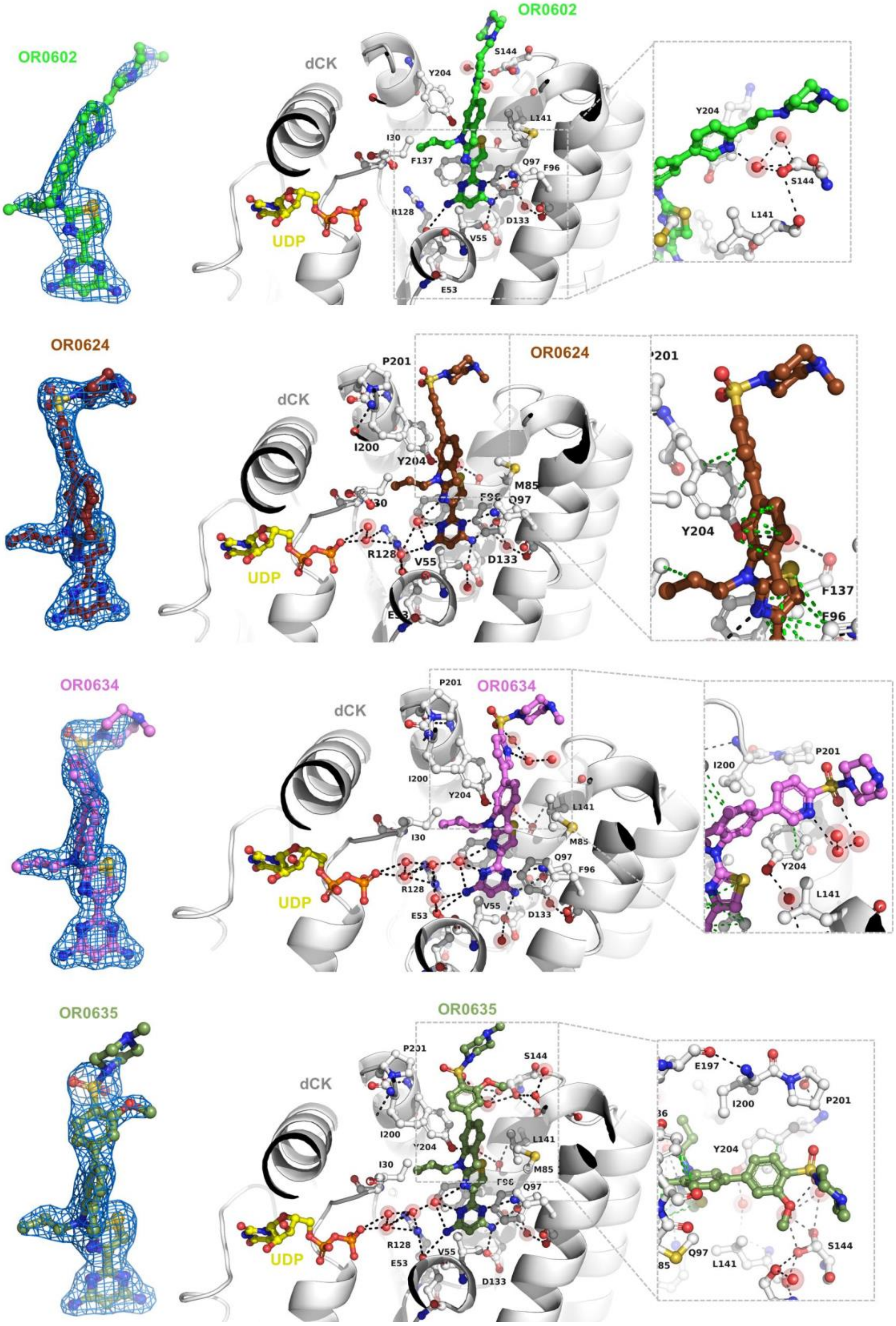

Supplementary Figure 6. Structure of dCK in complex with derivatives OR0602, OR0624, OR0634, and OR0635. Structure of dCK (grey ribbon) in complex with derivatives OR0602 (PDB 7ZI8), OR0624 (PDB 7ZI9), OR0634 (PDB 7ZIA), and OR0635 (PDB 7ZIB) and UDP (yellow) showing the hydrogen bond network to dCK (black dashed lines). The meshes around the compound (left) shows the |2Fo|−|Fc| electron density map contoured at 2.0σ around the ligand (blue). Hydrogen bonds between compounds and dCK are shown as dashed lines in the zoom inserts (right) and vdW interactions are shown as green dashed lines.

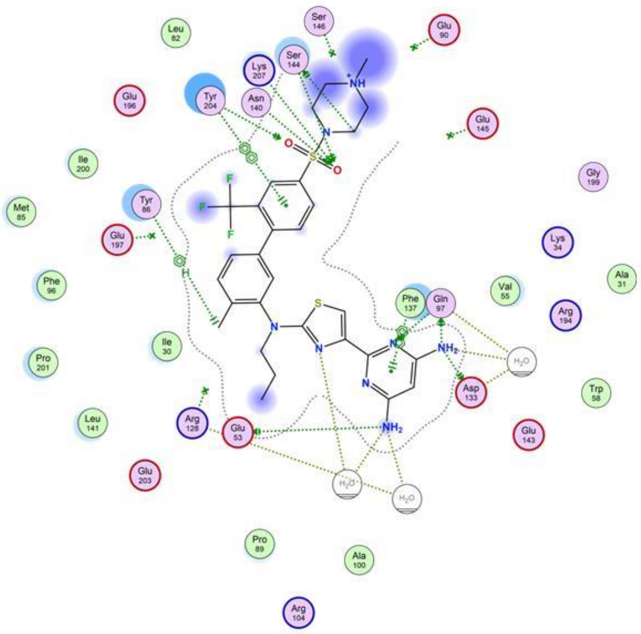

OR0642

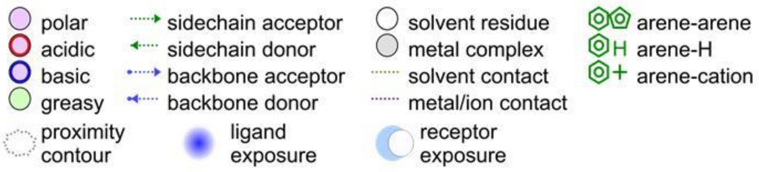

Supplementary Figure 7. Structure of dCK in complex with OR0642. Protein-ligand interaction diagrams calculated with the Molecular Operating Environment software (MOE) for dCK in complex with OR0642 (PDB 7ZI3).

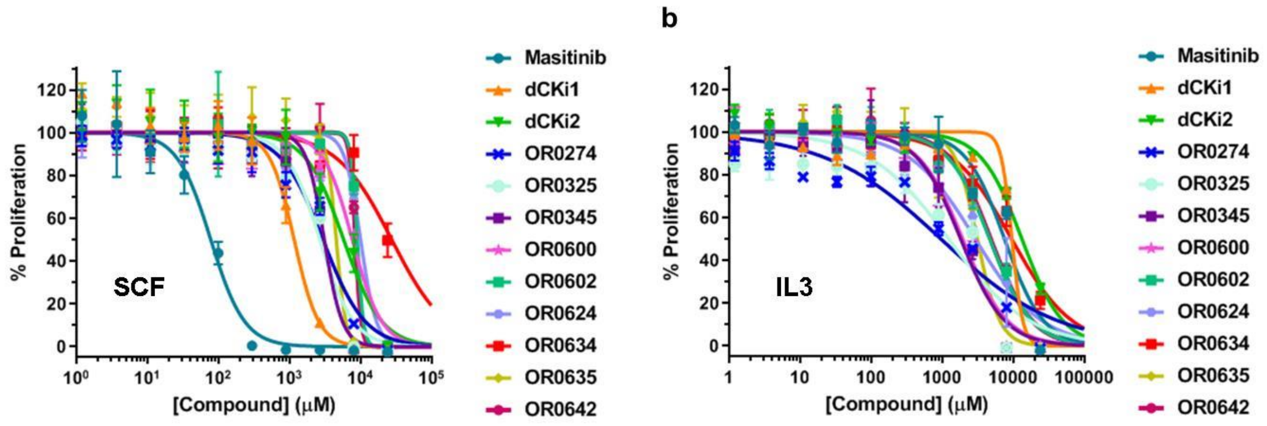

c

	IC50 (nM)		Ratio IL3/SCF
	SCF	IL3	
**AB1010**	78 ± 10	8250 ± 927	105.6
**dCKi1**	1232 ± 189	9329 ± 220	7.6
**dCKi2**	6498 ± 1121	17746 ± 4522	2.7
**OR0274**	2470 ± 397	1629 ± 599	0.7
**OR0325**	2362 ± 390	1312 ± 475	0.6
**OR0345**	2574 ± 488	1546 ± 318	0.6
**OR0600**	6560 ± 1041	2047 ± 365	0.3
**OR0602**	9136 ± 241	4761 ± 171	0.5
**OR0624**	8582 ± 2498	3364 ± 560	0.4
**OR0634**	22670 ± 6306	8489 ± 886	0.4
**OR0635**	4449 ± 810	3040 ± 113	0.7
**OR0642**	8382 ± 459	4459 ± 600	0.5

Supplementary Figure 8 Effect of selected compounds on the c-KIT cellular model. a Effect of selected compounds on cell proliferation of the interleukin-3 (IL-3) dependent Ba/F3 cell line (expressing WT human c-KIT) in the absence of IL-3 and in the presence of the Cell Stem Factor (SCF) (proliferation depending on c-KIT) (72 h) (n = 3). **b** Effect of selected compounds on cell proliferation of the IL-3 dependent Ba/F3 cell line (expressing WT human c-KIT) in the presence of IL-3 (72 h) (n = 3). **c** IC_{50} values on cell proliferation of the IL3 dependent Ba/F3 cell line (expressing WT human c-KIT) in presence of IL-3 or SCF (72 h) and ratio between IL3 vs SCF showing the specificity for c-KIT.

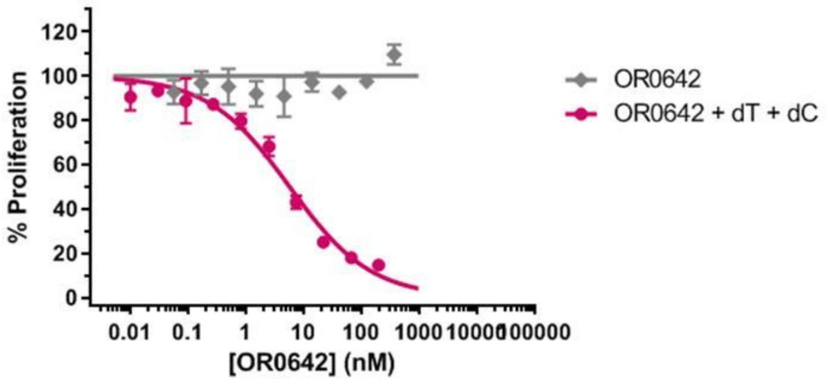

Supplementary Figure 9. OR0642 effect on CCRF-CEM cell line. Representative experiment displaying the effect of OR0642 on cell proliferation of the CCRF-CEM cell line in the presence and absence of dT (200 µM) and dC (1 µM) (*n* = 3), showing the lack of non-specific toxicity with OR0642 in the absence of dT+dC.

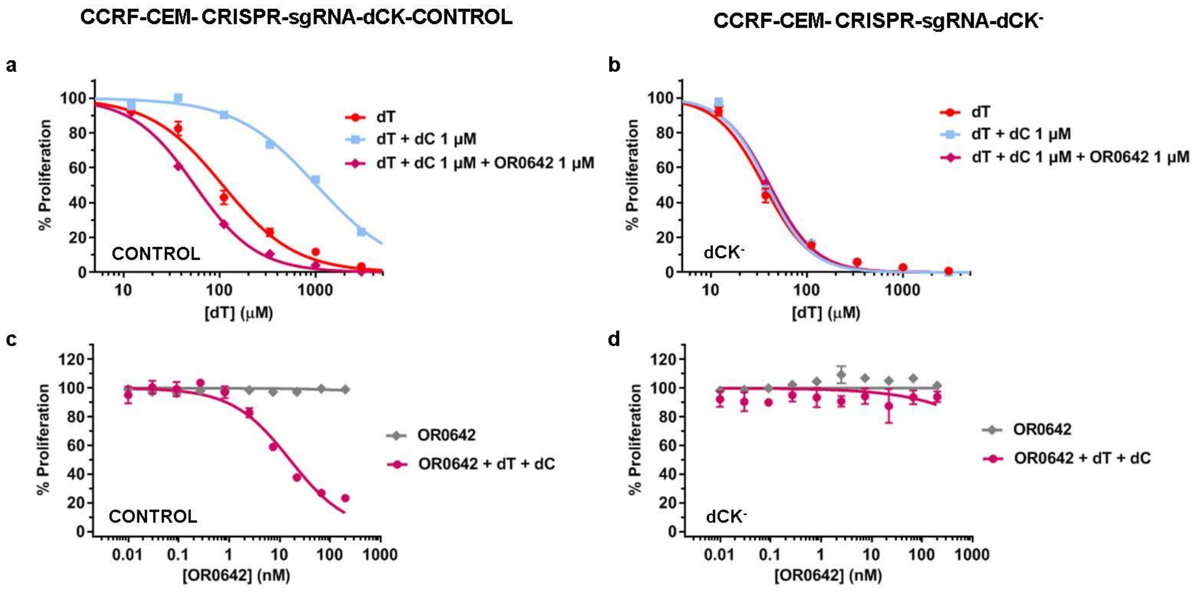

Supplementary Figure 10. OR0642 loss its activity on the CCRF-CEM CRISPR Cas9 cell line deficient for dCK. Representative experiments showing dT titrations in the CCRF-CEM cell lines (CRISPR-sgRNA-dCK-CONTROL (**a**) and CRISPR-sgRNA-dCK$^-$ (**b**)) in the absence and the presence of dC and OR0642 (1 µM). Representative experiment showing the effect of OR0642 on cell proliferation of the CCRF-CEM cell lines (CRISPR-sgRNA-dCK-CONTROL (**c**) and CRISPR-sgRNA-dCK$^-$ (**d**)) in presence and absence of dT (200 µM) and dC (1 µM) (*n* = 3).

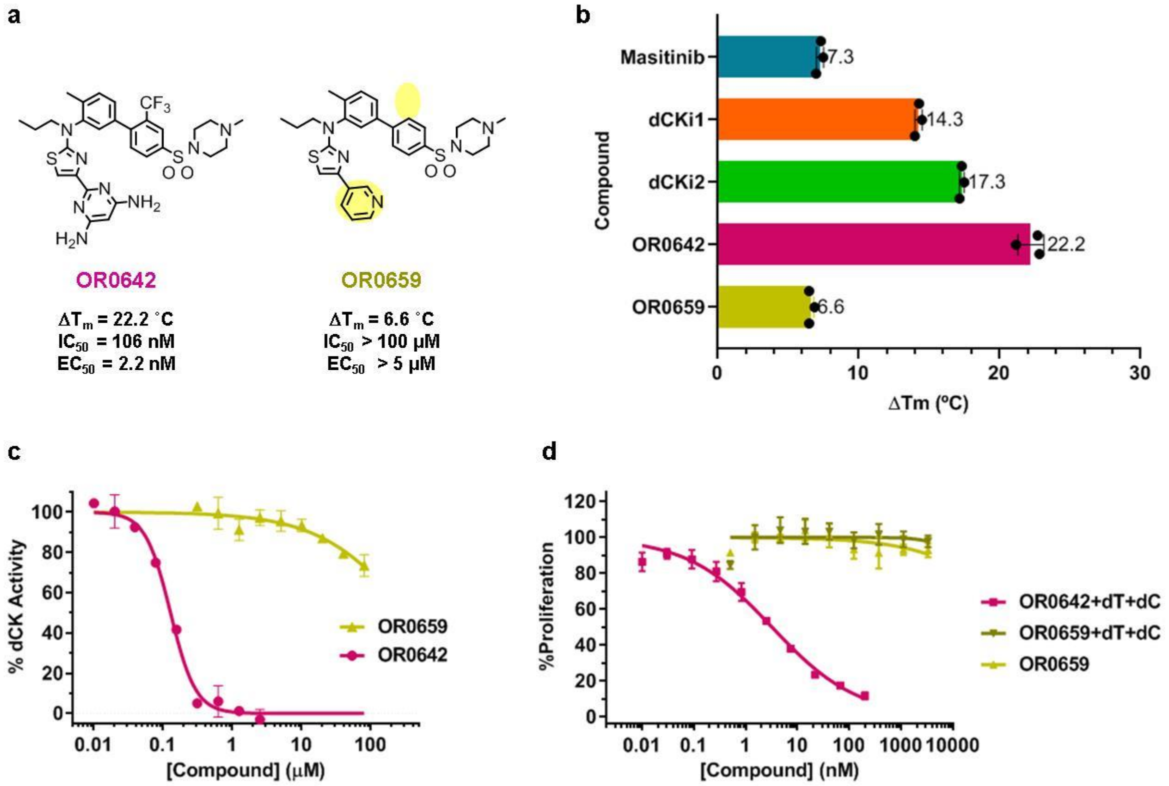

Supplementary Figure 11. Evaluation of the negative control compound OR0659 closely related to OR0642. a Chemical structures of OR0642 and OR0659. **b** dCK thermal stabilization by compounds measured by TSA. Data are presented as the mean ± SD (*n* = 3 independent experiments). **c** Representative experiment showing the effect of OR0659 on substrate phosphorylation by dCK in presence of ATP. Data are presented as the mean of three technical replicates ± SD. **d** Representative experiment showing the effect of OR0659 on cell proliferation of the CCRF-CEM cell line in absence and presence of dT (200 µM) and dC (1 µM) (*n* = 3).

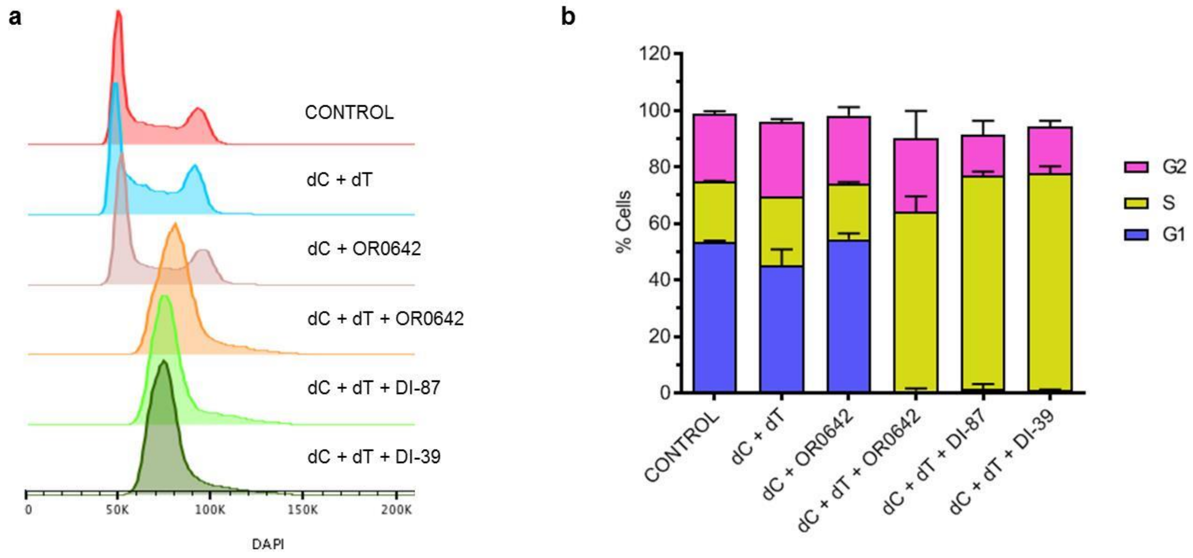

Supplementary Figure 12. The combination treatment dT+OR0642 induces cell cycle arrest. a Representative experiment showing cell cycle analysis of the CCRF-CEM cell line after treatment for 24h with the indicated compounds (dT (200 µM) and dC, OR0642, DI-87 and DI-39 (1 µM)). The DNA content of each population was analyzed by staining with DAPI (*n* = 3). **b** Stacked bar graphs showing the percentage of cells in different phases of the cell cycle (G1, S, and G2). Data are presented as the mean ± SD from *n* = 3 independent experiments.

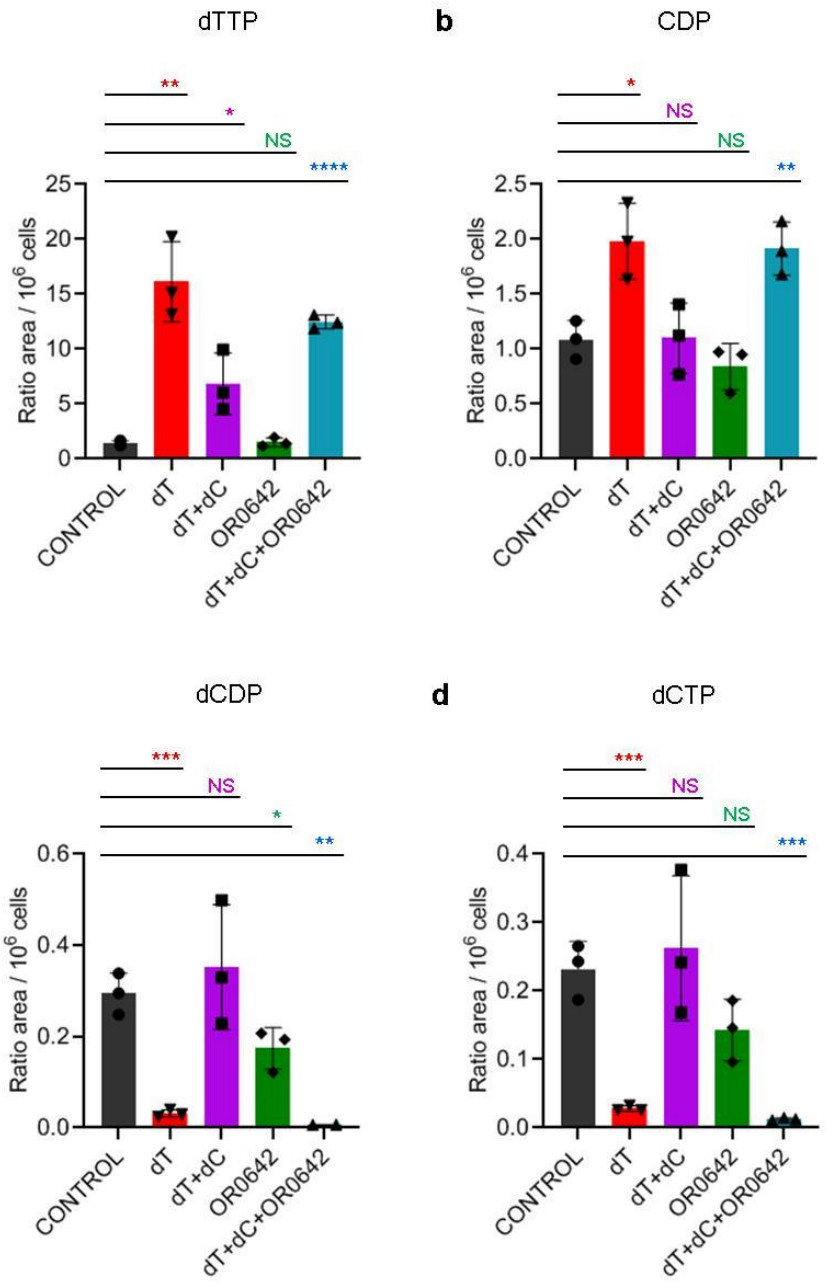

Supplementary Figure 13. The combination treatment dT+OR0642 induces nucleotide imbalance and cell cycle arrest. Nucleotide pools (**a** thymidine triphosphate (dTTP), **b** cytidine diphosphate (CDP), **c** deoxycytidine diphosphate (dCDP), **d** deoxycytidine triphosphate (dCTP)) determined by HRMS in the CCRF-CEM cells after treatment for 24h with the indicated compounds (dT (200 µM), dC (1 µM), and OR0642 (1 µM)). Ratio area = metabolite ^{12}C area / metabolite ^{13}C area. Data are presented as the mean ± SD from n = 3 independent experiments. Statistical significance is *$P < 0.05$, **$P < 0.01$, ***$P \leq 0.001$ or ****$P < 0.0001$ (two-tailed unpaired t-test).

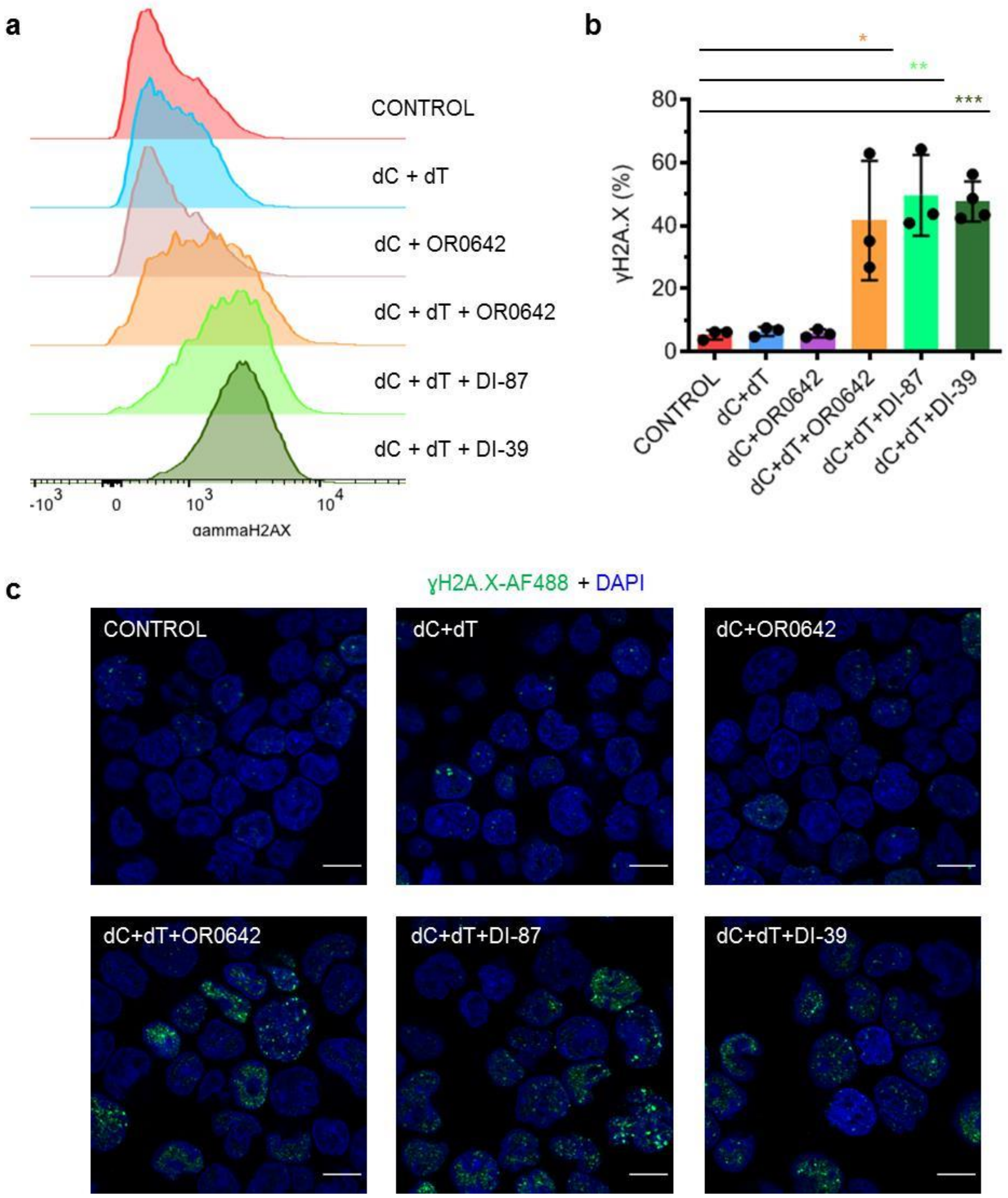

Supplementary Figure 14. dCK inhibitors combined with dT induce DNA double strand breaks. a Representative flow cytometry histograms showing γH2A.X levels in CCRF-CEM cells treated for 24h with the indicated compounds (dT (200 µM) and dC, OR0642, DI-87 and DI-39 (1 µM)). **b** Bar graph indicates the statistical quantification of γH2AX positive cells. All data are presented as the mean ± SD from n = 3 independent experiments. Statistical significance is *P = 0.02, **P = 0.004 or ***P = 0.0001 (two-tailed unpaired t test). **c** Representative images of CCRF-CEM cells treated as indicated. Green, γ-H2AX; blue, DNA stained with DAPI. Scale bars = 10 µm.

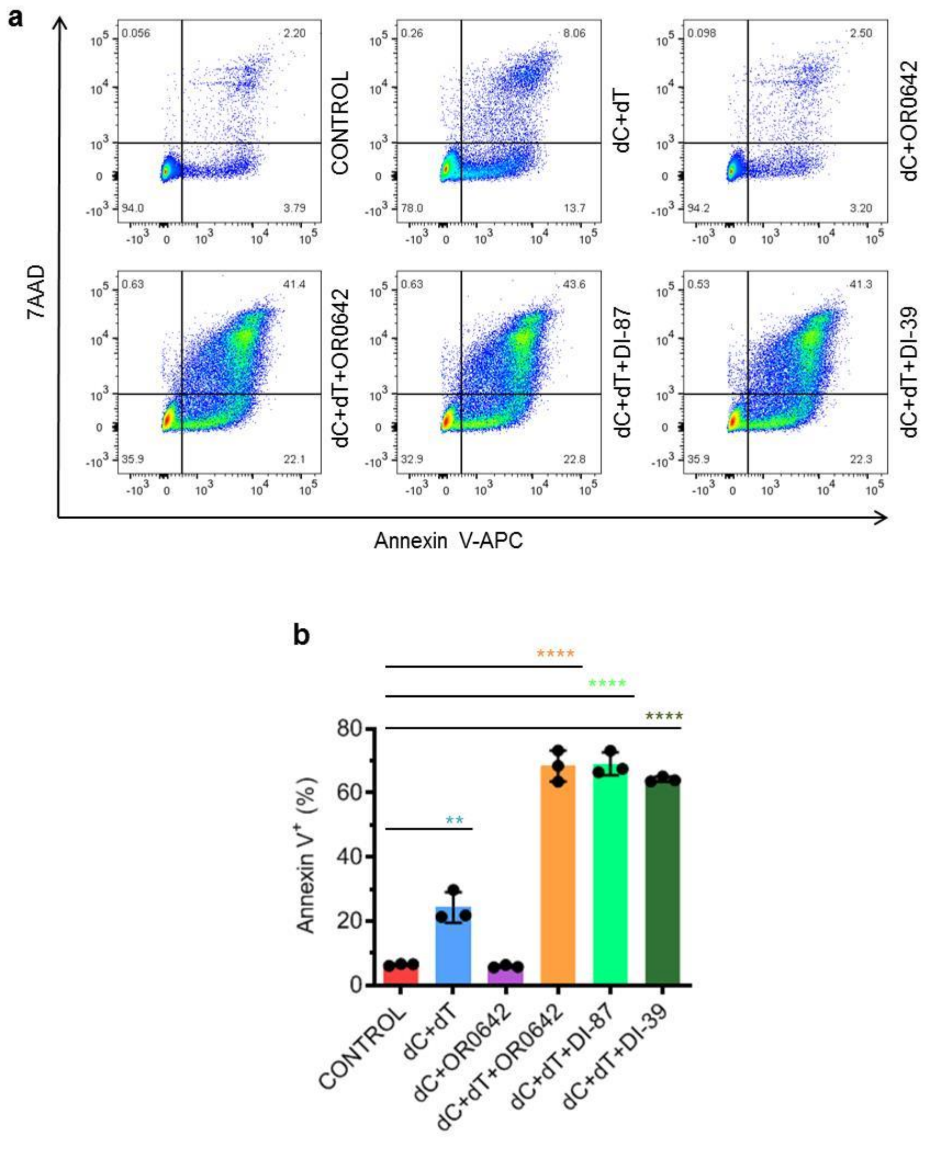

Supplementary Figure 15. OR0642 synergizes with dT to induce apoptosis. a Representative experiment showing the rate of apoptosis in CCRF-CEM cell line treated as indicated for 72 h (dT (200 µM) and dC, OR0642, DI-87 and DI-39 (1 µM)) measured by Annexin V staining determined by flow cytometry. Each square represents: live cells (bottom left), early apoptotic cells (bottom right), late apoptotic cells (top right), and necrotic cells (top left). **b** Bar graph indicates the statistical quantification of Annexin V positive cells. All data are presented as the mean ± SD (*n* = 3 biologically independent experiments). Statistical significance is $^{**}P = 0.002$ or $^{****}P < 0.0001$ (two-tailed unpaired t-test).

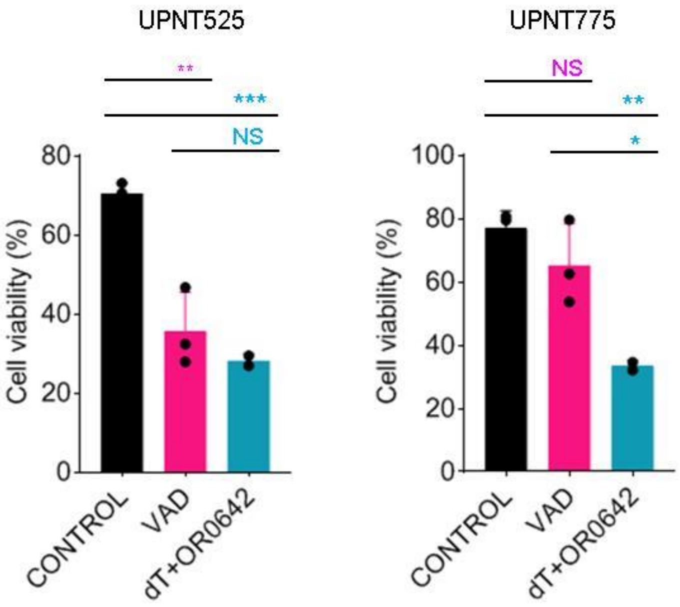

Supplementary Figure 16. Ex vivo evaluation of the combined therapy vs conventional chemotherapy (VAD). Effect of VAD: (vincristine (1 mg/ml), L-asparaginase (1 ui/ml) and dexamethasone (5 µM)) and the combined therapy dT+OR0642 (dT (333 µM) and OR0642 (250 nM)) on PDX cell lines (UPNT525 and UPNT775) treated with compounds. Cell viability was evaluated by flow cytometry 72h post-treatment. Data are presented as the mean ± SD (*n* = 3). NS represents no statistical significance, **P* = 0.04, ***P* < 0.01, and ****P* < 0.001 (two-tailed unpaired t-test).

Supplementary Note 1. Synthesis of compounds.

Synthetic procedures

Commercially available reagents and solvents were used without further additional purification. Thin layer chromatography (TLC) was performed on precoated aluminum sheets of silica (60 F254 nm, Merck) and visualized using short-wave UV light. Reaction monitoring and purity of compounds were recorded by using analytical Agilent Infinity high performance liquid chromatography with DAD at 254 nM Column Zorbax SB-C18 1.8 µM (2.1 x 50 mm), mobile phase (A: 0.1% FA H_2O, B: 0.1% FA MeCN), method (A) flow rate 0.3 mL/min, time/%B 0/10, 4/90, 7/90, 9/10, 10/10; method (B) flow rate 0.5 mL/min, time/%B 0/10, 4/90, 7/90, 9/10, 13/10); column Thermo Scientific Hypersil Gold 12 µm (4.6 x 250 mm), mobile phase (A: 0.1% TFA H_2O, B: 0.1% TFA MeCN), method (C) flow rate 2 mL/min, time/%B 0/10, 6/100, 11/100; column Agilent Poroshell 120 EC-C18 2.7µm (4.6 x 50 mm), mobile phase (A: 0.1% TFA H_2O, B: 0.1% TFA MeCN), method (D) flow rate 1 mL/min, time/%B 0/10, 5/100, 8/100. Column chromatography was performed on a Reveleris purification system using Reveleris Flash silica cartridges or C18 40 µM cartridges. Petroleum refers to the fraction with distillation range 40-65 °C. ^{1}H, ^{13}C NMR and ^{19}F NMR spectra were recorded by using a Bruker AC 400, AC300 or AC250 spectrometer. Chemical shifts (δ) are reported in ppm and coupling values (*J*) in hertz. Abbreviations for peaks are br: broad, s: singlet, d: doublet, t: triplet, q: quadruplet, quint: quintuplet, sex: sextuplet, and m: multiplet. The spectra recorded are consistent with the proposed structures. Low-resolution mass spectra were obtained with Agilent SQ G6120B mass spectrometer in positive and negative electrospray mode. All tested compounds yielded data consistent with a purity of ≥ 98%.

General methods for preparing new compounds are illustrated by Supplementary Figures 17-23.

Structural characterization of new compounds is detailed after the corresponding Supplementary Figure.

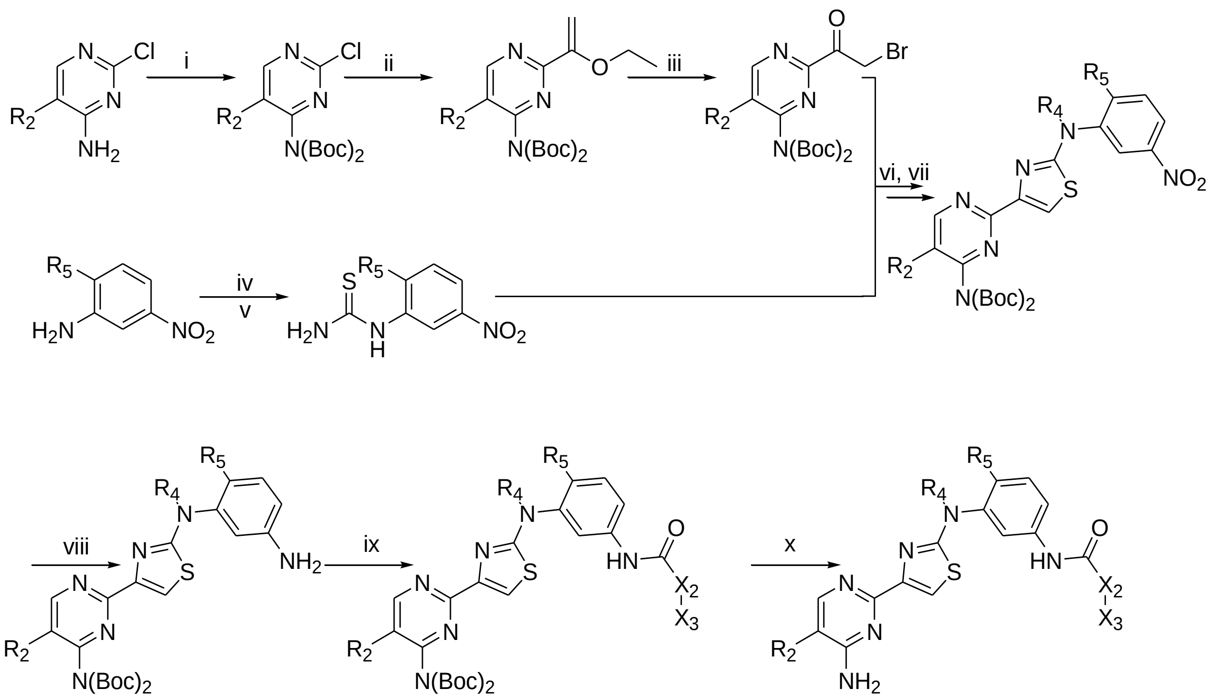

Supplementary Figure 17. Synthesis of *N*-(3-((4-(4-aminopyrimidin-2-yl)thiazol-2-yl)amino)-4-aryl) benzamide series. Reagents and conditions: i) Boc_2O, DMAP; ii) tributyl(1-ethoxyvinyl)tin, $Pd(PPh_3)_2Cl_2$, CsF, CuBr; iii) NBS; iv) KSCN, AcCl; v) K_2CO_3, EtOH; vi) K_2CO_3, EtOH; vii) optionally NaH, R_4Br; viii) Zn, AcOH or $SnCl_2.2H_2O$, EtOH; ix) HATU, DIPEA; x) TFA.

N-(3-((4-(4-aminopyrimidin-2-yl)thiazol-2-yl)amino)-4-aryl) benzamide derivatives were prepared by convergent synthesis, from commercially available 4-amino-2-chloropyrimidine and appropriate nitroaniline (**Supplementary Figure 17**). The 4-amino-2-chloropyrimidine was protected using Boc_2O to afford corresponding *bis*-carbamate. A Stille cross-coupling reaction with tributyl(1-ethoxyvinyl)tin gives the enol ether which was then turned into the corresponding α-bromoketone using *N*-bromosuccinimide. Starting from appropriate nitroaniline, condensation with acetyl isothiocyanate followed by saponification gave the corresponding thiourea. The α-bromoketone was engaged in the Hantzsch thiazole synthesis with the thiourea leading to the corresponding thiazole. Optionally R_4 as alkyl group can be introduced before reduction of the nitro group followed by peptide coupling with appropriate carboxylic acid affording expected amides. Finally, deprotection with TFA led to corresponding *N*-(3-((4-(4-aminopyrimidin-2-yl)thiazol-2-yl)amino)-4-aryl) benzamides.

General Procedure for the Synthesis of *N*-(3-((4-(4-aminopyrimidin-2-yl)thiazol-2-yl)amino)-4-aryl) benzamide Derivatives. A solution of *N*-(3-((4-(4-(di-*tert*-butoxycarbonylamino)pyrimidin-2-yl)thiazol-2-yl)amino)-4-aryl)benzamide derivative (0.1 mmol) in a mixture of dichloromethane–trifluoroacetic acid (3:1, 4 mL) was stirred at room temperature for 2 hrs. The solvent was distillated off under reduced pressure and the residue was purified by flash chromatography to afford the corresponding *N*-(3-((4-(4-aminopyrimidin-2-yl)thiazol-2-yl)amino)-4-aryl) benzamide.

***N*-(3-((4-(4-Aminopyrimidin-2-yl)thiazol-2-yl)amino)-4-methylphenyl)-4-((4-methylpiperazin-1-yl)methyl)benzamide dCKi-1** (78%) as a white powder. ^{1}H NMR (400 MHz, MeOD) δ 8.40 (d, *J* = 1.7 Hz, 1H), 8.13 (d, *J* = 6.0 Hz, 1H), 7.96 (d, *J* = 8.2 Hz, 2H), 7.66 (dd, *J* = 8.2, 1.7 Hz, 1H), 7.60 (s, 1H), 7.49 (d, *J* = 8.2 Hz, 2H), 7.21 (d, *J* = 8.2 Hz, 1H), 6.44 (d, *J* = 6.0 Hz, 1H), 3.61 (brs, 2H), 2.53 (brs, 8H), 2.32 (s, 3H), 2.30 (s, 3H); ^{13}C NMR (100 MHz, MeOD) δ 168.4, 167.5, 165.5, 161.2, 155.8, 151.1, 142.9, 140.7, 138.8, 135.4, 131.9, 130.6, 128.7, 125.5, 117.0, 113.9, 112.7, 104.6, 63.3, 55.7, 53.5, 45.9, 17.6; LCMS $C_{27}H_{30}FN_8OS$ method (D) R_t = 3.579 min, ESI+ m/z = 515.2 (M+H).

***N*-(1-(4-(4-Aminopyrimidin-2-yl)thiazol-2-yl)-2,3,4,5-tetrahydro-*1H*-benzo[b]azepin-8-yl)-4-((4-methylpiperazin-1-yl)methyl)benzamide OR0289** (66%) as a white solid. Rf = 0.40 (DCM-MeOH-NH_4OH, 85:13.5:1.5); ^{1}H NMR (400 MHz, MeOD) δ 8.08 (d, *J* = 6.0 Hz, 1H), 7.89 (d, *J* = 8.2 Hz, 2H), 7.83 (d, *J* = 2.0 Hz, 1H), 7.62 (dd, *J* = 8.2, 2.1 Hz, 1H), 7.44 (d, *J* = 8.2 Hz, 2H), 7.37 (s, 1H), 7.30 (d, *J* = 8.3 Hz, 1H), 6.41 (d, *J* = 6.0 Hz, 1H), 3.95 (m, 2H), 3.58 (s, 2H), 2.78-2.67 (m, 2H), 2.57 (s, 8H), 2.34 (s, 3H), 1.93 (m, 2H), 1.69 (m, 2H); ^{13}C NMR (100 MHz, MeOD) δ 170.78, 168.40, 165.47, 161.24, 155.30, 151.56, 146.10, 142.94, 139.56, 138.08, 135.09, 132.21, 130.50, 128.78, 121.77, 121.45, 112.18, 104.50, 63.08, 55.57, 53.20, 52.20, 45.63, 34.87, 29.52, 27.58; LC method (C) R_t = 2.822 min.

***N*-(3-((4-(4-Aminopyrimidin-2-yl)thiazol-2-yl)(propyl)amino)-4-methylphenyl)-4-((4-methylpiperazin-1-yl)methyl)benzamide OR0105** (86%) as a white solid. Rf = 0.18 (DCM-MeOH-NH_4OH, 90:9:1). ^{1}H NMR (400 MHz, MeOD) δ 8.14 (d, *J* = 6.0 Hz, 1H), 7.93 (d, *J* = 8.3 Hz, 2H), 7.78 (d, *J* = 2.0 Hz, 1H), 7.72 (dd, *J* = 8.3, 2.2 Hz, 1H), 7.51 (d, *J* = 8.2 Hz, 2H), 7.41 (d, *J* = 8.4 Hz, 1H), 7.38 (s, 1H), 6.45 (d, *J* = 6.0 Hz, 1H), 4.01 (brs, 2H), 3.64 (s, 2H), 2.57 (brs, 8H), 2.34 (s, 3H), 2.26 (s, 3H), 1.75 (sext, *J* = 7.4 Hz, 2H), 1.02 (t, *J* = 7.4 Hz, 3H); ^{13}C NMR (151 MHz, MeOD) δ 172.25, 168.59, 165.54, 161.49, 155.64, 151.60, 144.29, 143.05, 139.70, 135.15,

134.05, 133.35, 130.61, 128.71, 122.83, 122.41, 111.90, 104.51, 63.21, 54.85, 53.38, 45.82, 40.38, 22.26, 17.35, 11.64; LC method (C) R_t = 3.967 min.

***N*-(3-((4-(4-Aminopyrimidin-2-yl)thiazol-2-yl)(propyl)amino)-4-methylphenyl)-2-((4-methylpiperazin-1-yl)methyl)pyrimidine-5-carboxamide OR0125** (81%) as a light orange powder. Rf = 0.23 (DCM-MeOH-NH_4OH, 80:20:0.2); ^1H NMR (400 MHz, MeOD) δ 9.33 (s, 2H), 8.54 (d, *J* = 1.9 Hz, 1H), 8.14 (d, *J* = 6.0 Hz, 1H), 7.70 (dd, *J* = 8.2, 1.9 Hz, 1H), 7.63 (s, 1H), 7.21 (d, *J* = 8.2 Hz, 1H), 6.45 (d, *J* = 6.0 Hz, 1H), 3.91 (s, 2H), 2.82-2.46 (m, 8H), 2.33 (s, 6H); ^{13}C NMR (151 MHz, MeOD) δ 170.12, 165.54, 163.93, 161.24, 157.78, 155.72, 131.92, 128.42, 116.50, 113.37, 113.01, 104.68, 65.02, 55.47, 53.69, 49.00, 45.86, 17.59; LC method (D) R_t = 3.967 min.

***N*-(3-((4-(4-Aminopyrimidin-2-yl)thiazol-2-yl)amino)-4-methylphenyl)-4-(2-(4-methylpiperazin-1-yl)ethyl)benzamide OR0146** (49%) as orange crystals. Rf = 0.55 (DCM-MeOH-NH_4OH, 80:18:2). ^1H NMR (400 MHz, MeOD) δ 8.42 (d, *J* = 2.0 Hz, 1H), 8.10 (d, *J* = 6.0 Hz, 1H), 7.88 (d, *J* = 8.3 Hz, 2H), 7.61 (dd, *J* = 8.5, 2.0 Hz, 1H), 7.59 (s, 1H), 7.32 (d, *J* = 8.3 Hz, 2H), 7.16 (d, *J* = 8.5 Hz, 1H), 6.41 (d, *J* = 6.0 Hz, 1H), 2.88 – 2.81 (m, 2H), 2.65 – 2.59 (m, 2H), 2.59 (s, 8H), 2.32 (s, 3H), 2.29 (s, 3H); ^{13}C NMR (100 MHz, MeOD) δ 168.39, 167.34, 165.46, 160.96, 155.39, 150.83, 145.51, 140.60, 138.79, 134.16, 131.85, 129.92, 128.86, 125.26, 116.90, 113.78, 112.99, 104.63, 60.58, 55.45, 53.28, 45.69, 33.83, 17.63; LC method (D) R_t = 3.592 min.

***N*-(3-((4-(4-Amino-5-fluoropyrimidin-2-yl)thiazol-2-yl)amino)-4-methylphenyl)-4-((4-methylpiperazin-1-yl)methyl)benzamide OR0239** (57%) as a white powder. ^1H NMR (400 MHz, MeOD) δ 8.33 (d, *J* = 1.8 Hz, 1H), 8.10 (d, *J* = 3.6 Hz, 1H), 7.95 (d, *J* = 8.2 Hz, 2H), 7.64 (dd, *J* = 8.3, 1.8 Hz, 1H), 7.54 (s, 1H), 7.49 (d, *J* = 8.2 Hz, 2H), 7.21 (d, *J* = 8.3 Hz, 1H), 3.62 (brs, 2H), 2.52 (brs, 8H), 2.32 (s, 3H), 2.29 (s, 3H) ; ^{13}C NMR (151 MHz, MeOD) δ 168.45, 167.87, 156.41 (d, *J* = 252 Hz), 150.58, 146.77, 145.06, 142.92, 140.67, 139.89 (d, *J* = 19.6 Hz), 138.81, 135.37, 131.93, 130.61, 128.70, 126.02, 117.36, 114.34, 112.27, 63.28, 55.73, 53.58, 45.95, 17.56; LC method (D) R_t = 3.548 min

***N*-(3-((4-(4-Aminopyrimidin-2-yl)thiazol-2-yl)amino)-4-methylphenyl)-4-((dimethylamino)methyl) benzamide OR0155.** (77%) as a white solid. Rf = 0.20 (DCM-MeOH-NH_4OH, 90:9:1); ^1H NMR (400 MHz, MeOD) δ 8.39 (s, 1H), 8.11 (s, 1H), 7.96 (d, *J* = 8.1 Hz, 2H), 7.62 (m, 1H), 7.60 (s, 1H), 7.47 (d, *J* = 8.2 Hz, 2H), 7.20 (d, *J* = 8.0 Hz, 1H), 6.43 (d, *J* = 6.1 Hz,

1H), 3.60 (s, 2H), 2.31 (s, 3H), 2.30 (s, 6H); ^{13}C NMR (100 MHz, MeOD) δ 168.32, 167.53, 165.52, 161.13, 155.60, 152.54, 142.42, 140.58, 138.80, 135.63, 131.92, 130.83, 128.81, 125.52, 117.08, 113.97, 112.80, 104.67, 64.30, 45.19, 17.59; LC method (D) R_t = 3.703 min.

***N*-(3-((4-(4-Aminopyrimidin-2-yl)thiazol-2-yl)amino)-4-methylphenyl)-4-((6-methyl-2,6-diazaspiro[3.3]heptan-2-yl)methyl)benzamide OR0156** (10% over 2 steps) as a white solid. Rf = 0.18 (DCM-MeOH-NH_4OH, 80:20:2). ^{1}H NMR (400 MHz, MeOD) δ 8.42 (d, *J* = 2.0 Hz, 1H), 8.13 (d, *J* = 6.0 Hz, 1H), 7.96 (d, *J* = 8.1 Hz, 2H), 7.63 (s, 1H), 7.52 (dd, *J* = 8.2, 2.0 Hz, 1H), 7.43 (d, *J* = 8.1 Hz, 2H), 7.21 (d, *J* = 8.3 Hz, 1H), 6.46 (d, *J* = 6.1 Hz, 1H), 3.98 (s, 4H), 3.72 (s, 2H), 3.49 (s, 4H), 2.69 (s, 3H), 2.33 (s, 3H); ^{13}C NMR (151 MHz, MeOD) δ 168.27, 167.61, 165.60, 155.18, 138.74, 131.89, 129.99, 128.94, 125.79, 117.06, 114.12, 113.04, 104.65, 66.16, 64.05, 63.11, 49.57, 35.26, 17.57; LCMS $C_{28}H_{30}N_8OS$ method (B) R_t = 4.933 min, ESI+ m/z = 527.3 (M+H).

***N*-(3-((4-(4-Aminopyrimidin-2-yl)thiazol-2-yl)amino)-4-methylphenyl)-5-((4-methylpiperazin-1-yl)methyl)thiophene-2-carboxamide OR0241** (83%) as a white powder. ^{1}H NMR (400 MHz, DMSO-d_6) δ 10.17 (s, 1H), 9.37 (brs, 1H), 8.08 (d, *J* = 5.8 Hz, 1H), 7.98 (d, *J* = 1.8 Hz, 1H), 7.83 (d, *J* = 3.8 Hz, 1H), 7.47 (s, 1H), 7.45 (dd, *J* = 8.4, 1.8 Hz, 1H), 7.20 (d, J = 8.4 Hz, 1H), 7.02 (d, *J* = 3.8 Hz, 1H), 6.79 (brs, 2H), 6.31 (d, *J* = 5.8 Hz, 1H), 3.68 (s, 2H), 2.42 (brs, 4H), 2.33 (brs, 4H), 2.23 (s, 3H), 2.15 (s, 3H); ^{13}C NMR (151 MHz, MeOD) δ 167.36, 165.53, 162.63, 161.24, 155.75, 151.10, 148.92, 140.71, 140.49, 138.57, 131.86, 129.87, 128.34, 125.17, 116.71, 113.48, 112.86, 104.68, 57.78, 55.77, 53.28, 45.88, 17.56; LC method (D) R_t = 3.725 min.

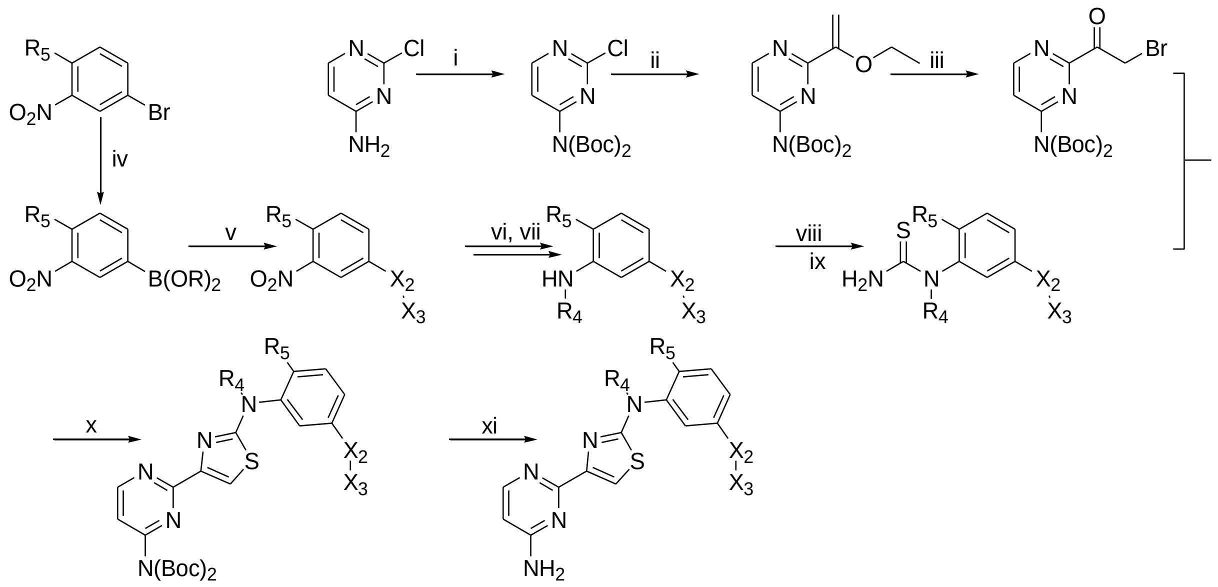

Supplementary Figure 18. Synthesis of *N*-([1,1'-biaryl]-3-yl)-4-(4-aminopyrimidin-2-yl)thiazol-2-amine series. Reagents and conditions: i) Boc_2O, DMAP; ii) tributyl(1-ethoxyvinyl)tin, $Pd(PPh_3)_2Cl_2$, CsF, CuBr; iii) NBS; iv) $(PinB)_2$, $PdCl_2$(dppf), KOAc; v) $ArB(OH)_2$, $PdCl_2$(dppf), Na_2CO_3; vi) H_2, Pd/C, THF-EtOH; vii) optionally RCHO, $NaBH(OAc)_3$, AcOH; viii) KSCN, AcCl or 1N HCl_{aq}., or PhCONCS; ix) K_2CO_3, MeOH; x) K_2CO_3, EtOH; xi) TFA.

N-([1,1'-biaryl]-3-yl)-4-(4-aminopyrimidin-2-yl)thiazol-2-amine derivatives were prepared by convergent synthesis, in eight steps, from commercially available 4-amino-2-chloropyrimidine and appropriate bromo-3-nitrobenzene (**Supplementary Figure 18**). The 4-amino-2-chloropyrimidine was protected using Boc_2O to afford corresponding *bis*-carbamate. A Stille cross-coupling reaction with tributyl(1-ethoxyvinyl)tin gave the enol ether which was then turned into the corresponding α-bromoketone using *N*-bromosuccinimide. Starting from appropriate bromo-3-nitrobenzene, a Suzuki cross-coupling reaction with corresponding aryl boronate allowed introducing the key biaryl scaffold. Hydrogenation of the nitro group followed by condensation with potassium thiocyanate, after optional alkylation, gave the corresponding thiourea. As previously described, the α-bromoketone was engaged in the Hantzsch thiazole synthesis with the thiourea leading to the corresponding thiazole. Finally, deprotection with TFA led to expected *N*-([1,1'-biaryl]-3-yl)-4-(4-aminopyrimidin-2-yl)thiazol-2-amines.

General Procedure for the Synthesis of *N*-([1,1'-biaryl]-3-yl)-4-(4-aminopyrimidin-2-yl)thiazol-2-amine. As previously described for *N*-(3-((4-(4-aminopyrimidin-2-yl)thiazol-2-yl)amino)-4-aryl) benzamide derivatives.

4-(4-Aminopyrimidin-2-yl)-*N*-(4-methyl-4'-((4-methylpiperazin-1-yl)methyl)-[1,1'-biphenyl]-3-yl)thiazol-2-amine OR0274 (57%) as a white powder. ^{1}H NMR (400 MHz, CDCl3) δ 8.31 (d, *J* = 5.7 Hz, 1H), 7.76 (s, 1H), 7.59 (s, 1H), 7.55 (d, *J* = 8.1 Hz, 2H), 7.39 (d, *J* = 8.1 Hz, 2H), 7.32-7.27 (m, 2H), 6.34 (d, *J* = 5.7 Hz, 1H), 4.98 (brs, 2H), 3.55 (s, 2H), 2.50 (brs, 8H), 2.34 (s, 3H), 2.30 (s, 3H); ^{13}C NMR (100 MHz, $CDCl_3$) δ 166.65, 163.11, 160.54, 156.49, 150.57, 140.32, 139.31, 139.17, 137.56, 131.73, 129.81, 128.70, 126.88, 123.25, 118.96, 110.94, 103.41, 62.75, 55.20, 53.11, 46.07, 17.69. LC method (D) R_t = 3.558 min.

4-(4-Aminopyrimidin-2-yl)-*N*-(4-methyl-4'-(2-(4-methylpiperazin-1-yl)ethyl)-[1,1'-biphenyl]-3-yl)thiazol-2-amine OR0325 (45%) as a white powder. ^{1}H NMR (400 MHz, $CDCl_3$) δ 8.30 (d, *J* = 5.7 Hz, 1H), 7.74 (s, 1H), 7.58 (s, 1H), 7.52 (d, *J* = 8.2 Hz, 2H), 7.35 (brs, 1H), 7.29-7.27 (m, 4H), 6.33 (d, *J* = 5.7 Hz, 1H), 4.99 (s, 2H), 2.85 (dd, *J* = 9.9, 6.4 Hz, 1H), 2.65 (dd, *J* = 9.9, 6.4 Hz, 1H), 2.52 (brs, 8H).2.33 (s, 3H), 2.32 (s, 3H). ^{13}C NMR (100 MHz, $CDCl_3$) δ 166.66, 163.10, 160.53, 156.48, 150.57, 140.34, 139.73, 139.16, 138.35, 131.71, 129.32, 128.62, 127.05, 123.19, 118.92, 110.92, 103.40, 60.47, 55.19, 53.20, 46.11, 33.31, 17.67; LC method (D) R_t = 3.602 min.

3'-((4-(4-Aminopyrimidin-2-yl)thiazol-2-yl)amino)-4'-methyl-[1,1'-biphenyl]-4-ol OR0320 (58%) as a white powder. ^{1}H NMR (400 MHz, MeOD) δ 8.12 (d, *J* = 6.0 Hz, 1H), 7.76 (d, *J* = 1.6 Hz, 1H), 7.49 (s, 1H), 7.46 (d, *J* = 8.6 Hz, 2H), 7.32 (dd, *J* = 8.1, 1.6 Hz, 1H), 7.29 (d, *J* = 8.1 Hz, 1H). 6.84 (d, *J* = 8.6 Hz, 2H), 6.43 (d, *J* = 6.0 Hz, 1H), 2.34 (s, 3H). ^{13}C NMR (100 MHz, MeOD) δ 169.65, 165.60, 160.77, 158.24, 154.87, 150.72, 141.44, 140.88, 133.12, 132.59, 130.83, 128.88, 124.35, 122.07, 116.64, 112.10, 104.58, 17.61; LC method (D) R_t = 4.369 min.

3'-((4-(4-Aminopyrimidin-2-yl)thiazol-2-yl)amino)-4'-methyl-[1,1'-biphenyl]-4-ol OR0321 (53%) as a white powder. ^{1}H NMR (400 MHz, $CDCl_3$) δ 8.28 (s, 1H), 7.70 (s, 1H), 7.57 (s, 1H), 7.28-7.26 (m, 2H), 7.12-6.96 (m, 3H), 6.33 (d, *J* = 5.5 Hz, 1H), 5.03 (brs, 2H), 3.94 (s, 3H), 2.32 (s, 3H); ^{13}C NMR (151 MHz, MeOD) δ 169.45, 165.59, 160.77, 155.33, 151.06, 149.27, 147.38, 141.54, 140.87, 138.26, 132.52, 131.75, 130.66, 124.32, 120.56, 116.59, 112.02, 111.47, 104.61, 56.51, 17.61; LC method (D) R_t = 4.389 min.

4-(4-Aminopyrimidin-2-yl)-*N*-(4-methyl-4'-(3-(4-methylpiperazin-1-yl)propyl)-[1,1'-biphenyl]-3-yl)thiazol-2-amine OR0331 (46%) as a light yellow powder. ^{1}H NMR (400 MHz, $CDCl_3$) δ 8.26 (d,

J = 5.7 Hz, 1H), 7.74 (brs, 1H), 7.56 (s, 1H), 7.51-7.49 (m, 3H), 7.31-7.24 (m, 4H), 6.31 (d, *J* = 5.7 Hz, 1H), 5.05 (brs, 2H), 2.66 (t, *J*= 7.8 Hz, 2H), 2.47 (brs, 8H), 2.39 (t, *J* = 7.8 Hz, 2H), 2.31 (s, 3H), 2.28 (s, 3H), 1.81 (quint, *J* = 7.8 Hz, 2H); ^{13}C NMR (100 MHz, $CDCl_3$) δ 166.67, 163.10, 160.55, 156.51, 150.56, 141.65, 140.45, 139.12, 138.04, 131.71, 129.02, 128.51, 126.97, 123.20, 118.89, 110.97, 103.40, 58.08, 55.26, 53.29, 46.16, 33.47, 28.71, 17.68; LCMS $C_{28}H_{33}N_7S$ method (B) R_t = 3.881 min, ESI+ m/z = 500.1 (M+H).

4-(4-Aminopyrimidin-2-yl)-*N*-(4-methyl-4'-(3-(4-methylpiperazin-1-yl)ethyl)-[1,1'-biphenyl]-3-yl)-*N*-propylthiazol-2-amine OR0345 (42%), as a colorless solid; ^{1}H NMR (400 MHz, $CDCl_3$) δ 8.26 (d, *J* = 5.7 Hz, 1H), 7.53-7.46 (m, 4H), 7.39-7.38 (m, 2H), 7.28-7.25 (m, 2H), 6.25 (d, *J* = 5.7 Hz, 1H), 5.3 (brs, 2H), 4.01 (brs, 2H), 2.86-2.82 (m, 2H), 2.87-2.51 (m, 2H), 2.52 (brs, 8H), 2.31 (s, 3H), 2.26 (s, 3H), 1.66 (sext, *J* = 7.3 Hz, 2H), 0.92 (t, *J* = 7.3 Hz, 3H); ^{13}C NMR (100 MHz, $CDCl_3$) δ 171.27, 163.14, 160.88, 156.41, 151.19, 143.27, 140.74, 139.81, 137.84, 136.17, 132.49, 129.33, 127.97, 126.96, 111.21, 103.18, 60.39, 55.13, 53.61, 53.11, 46.05, 33.25, 21.27, 17.45, 11.40; LCMS $C_{30}H_{37}N_7S$ method (B) R_t = 4.152 min, ESI+ m/z = 528.3 (M+H).

4-(4-Aminopyrimidin-2-yl)-*N*-(4-methyl-4'-(3-(4-methylpiperazin-1-yl)propyl)-[1,1'-biphenyl]-3-yl)-*N*-propylthiazol-2-amine OR0598 (70%), as a pale yellow solid. Rf = 0.40 (DCM-MeOH-NH_4OH, 90:9:1); ^{1}H NMR (400 MHz, $CDCl_3$) δ 8.29 (d, *J* = 5.8 Hz, 1H), 7.53 (dd, *J* = 7.9, 1.7 Hz, 1H), 7.49 (d, *J* = 8.1 Hz, 2H), 7.46 (d, *J* = 1.7 Hz, 1H), 7.39 (d, *J* = 7.6 Hz, 1H), 7.37 (s, 1H), 7.24 (d, *J* = 8.1 Hz, 2H), 6.29 (d, *J* = 5.8 Hz, 1H), 5.10 (s, 2H), 4.02 (brs, 2H), 2.72-2.65 (m, 2H), 3.11-2.55 (m, 8H), 2.54-2.45 (m, 2H), 2.43 (s, 3H), 2.27 (s, 3H), 1.89 (quint, *J* = 7.6 Hz, 2H), 1.67 (sext, *J* = 7.5 Hz, 2H), 0.93 (t, *J* = 7.5 Hz, 3H). ^{13}C NMR (100 MHz, $CDCl_3$) δ 171.41, 163.09, 160.94, 156.53, 151.17, 143.29, 141.14, 140.78, 137.78, 136.24, 132.54, 129.04, 128.02, 127.00, 111.31, 103.19, 57.48, 54.33, 53.53, 52.03, 45.29, 33.18, 28.07, 21.32, 17.47, 11.44; LCMS $C_{31}H_{39}N_7S$ method (B) R_t = 4.253 min, ESI+ m/z = 542.3 (M+H).

2-(2-(8-(4-(2-(4-Methylpiperazin-1-yl)ethyl)phenyl)-2,3,4,5-tetrahydro-1H-benzo[b]azepin-1-yl)thiazol-4-yl) pyrimidin-4-amine OR0402 (87%), as a pale yellow solid. Rf = 0.18 (DCM-MeOH-NH_4OH, 90:9:1); ^{1}H NMR (400 MHz, MeOD) δ 8.12 (d, *J* = 6.0 Hz, 1H), 7.60 (d, *J* = 1.8 Hz, 1H), 7.56-7.47 (m, 3H), 7.38 (d, *J* = 7.9 Hz, 1H), 7.35 (s, 1H), 7.27 (d, *J* = 8.2 Hz, 2H), 6.43 (d, *J* = 6.0 Hz, 1H), 4.03 (brs, 2H), 2.85-2.79 (m, 2H), 2.78-2.72 (m, 2H), 2.66-2.58 (m, 2H), 2.55 (brs, 8H), 2.30 (s, 3H), 1.95-1.93 (m, 2H), 1.75-7.73 (m, 2H). ^{13}C NMR (100 MHz, MeOD) δ 170.97, 165.49, 161.54, 155.76, 151.89, 146.59, 142.09, 141.04, 140.70, 139.12, 132.64, 130.35, 127.88, 127.59,

127.25, 111.86, 104.49, 61.16, 55.53, 53.55, 52.04, 45.89, 35.03, 33.63, 29.59, 27.58; LCMS $C_{30}H_{35}N_7S$ method (B) R_t = 4.064 min, ESI+ m/z = 526.3 (M+H).

4-(4-Aminopyrimidin-2-yl)-*N*-(4-methyl-4'-(2-(4-methylpiperazin-1-yl)ethoxy)-[1,1'-biphenyl]-3-yl)-*N*-propylthiazol-2-amine OR0596 (63%), as a white solid. ^{1}H NMR (400 MHz, $CDCl_3$) δ 8.29 (d, *J* = 5.8 Hz, 1H), 7.50 (d, *J* = 8.8 Hz, 2H), 7.49 (dd, *J* = 7.7, 1.8 Hz, 1H), 7.43 (d, *J* = 2.0 Hz, 1H), 7.38 (s, 1H), 7.37 (d, *J* = 7.7 Hz, 1H), 6.97 (d, *J* = 8.8 Hz, 2H), 6.27 (d, *J* = 5.8 Hz, 1H), 5.12 (s, 2H), 4.14 (t, *J* = 5.8 Hz, 2H), 4.02 (brs, 2H), 2.83 (t, *J* = 5.8 Hz, 2H), 2.63 (brs, 4H), 2.48 (brs, 4H), 2.29 (s, 3H), 2.26 (s, 3H), 1.67 (sext, *J* = 7.4 Hz, 2H), 0.93 (t, *J* = 7.4 Hz, 3H). ^{13}C NMR (100 MHz, $CDCl_3$) δ 171.39, 163.09, 160.98, 158.62, 156.58, 151.19, 143.28, 140.59, 135.71, 132.73, 132.49, 128.02, 127.70, 126.75, 115.12, 111.27, 103.16, 66.17, 57.28, 55.16, 53.73, 53.56, 46.16, 21.31, 17.44, 11.44; LCMS $C_{30}H_{37}N_7S$ method (B) R_t = 4.304 min, ESI+ m/z = 544.2 (M+H).

4-(4-Aminopyrimidin-2-yl)-*N*-(4-methyl-4'-(2-(4-methylpiperazin-1-yl)-2-oxoethoxy)-[1,1'-biphenyl]-3-yl)-*N*-propylthiazol-2-amine OR0597 (49%), as a white solid. ^{1}H NMR (400 MHz, $CDCl_3$) δ 8.30 (d, *J* = 5.8 Hz, 1H), 7.51 (d, *J* = 8.8 Hz, 2H), 7.48 (dd, *J* = 8.0, 1.9 Hz, 1H), 7.42 (d, *J* = 1.9 Hz, 1H), 7.38 (s, 1H), 7.38 (d, *J* = 8.0 Hz, 1H), 7.01 (d, *J* = 8.8 Hz, 2H), 6.29 (d, *J* = 5.8 Hz, 1H), 5.04 (s, 2H), 4.72 (s, 2H), 4.03 (brs, 2H), 3.63 (m, 4H), 2.40 (m, 4H), 2.29 (s, 3H), 2.27 (s, 3H), 1.67 (sext, *J* = 7.4 Hz, 2H), 0.94 (t, *J* = 7.4 Hz, 3H). ^{13}C NMR (100 MHz, $CDCl_3$) δ 171.41, 166.40, 163.07, 161.00, 157.69, 156.63, 151.19, 143.30, 140.40, 135.97, 133.65, 132.55, 128.22, 127.81, 126.83, 115.16, 111.31, 103.16, 67.85, 55.27, 54.74, 53.51, 46.15, 45.39, 42.18, 21.33, 17.45, 11.45; LCMS $C_{30}H_{35}N_7O_2S$ method (B) R_t = 4.296 min, ESI+ m/z = 558.2 (M+H).

4-(4-Aminopyrimidin-2-yl)-*N*-(2-methyl-5-(6-(2-(4-methylpiperazin-1-yl)ethyl)pyridin-3-yl)phenyl)-*N*-propylthiazol-2-amine OR0599 (10%) as a yellow solid. Rf = 0.40 (DCM-MeOH-NH_4OH, 90:9:1); ^{1}H NMR (400 MHz, $CDCl_3$) δ 8.74 (s, 1H), 8.30 (d, *J* = 5.3 Hz, 1H), 7.77 (d, *J* = 7.9 Hz, 1H), 7.52 (d, *J* = 7.9 Hz, 1H), 7.45 (s, 1H), 7.43 (d, *J* = 7.9 Hz, 1H), 7.39 (s, 1H), 7.24 (d, *J* = 7.9 Hz, 1H), 6.29 (d, *J* = 5.3 Hz, 1H), 5.07 (s, 2H), 4.04 (brs, 2H), 3.03 (m, 2H), 2.80 (m, 2H), 2.60 (brs, 4H), 2.48 (brs, 4H), 2.29 (s, 6H), 1.66 (sext, *J* = 7.2 Hz, 2H), 0.94 (t, *J* = 7.2 Hz, 3H). ^{13}C NMR (100 MHz, $CDCl_3$) δ 171.20, 163.08, 160.97, 159.62, 156.65, 151.28, 147.57, 143.60, 137.68, 137.31, 134.70, 133.15, 132.92, 128.14, 127.01, 123.28, 111.35, 103.20, 58.43, 55.24, 53.54, 53.16, 46.15, 35.53, 21.36, 17.56, 11.44; LCMS $C_{29}H_{36}N_8S$ method (B) R_t = 3.950 min, ESI+ m/z = 529.4 (M+H).

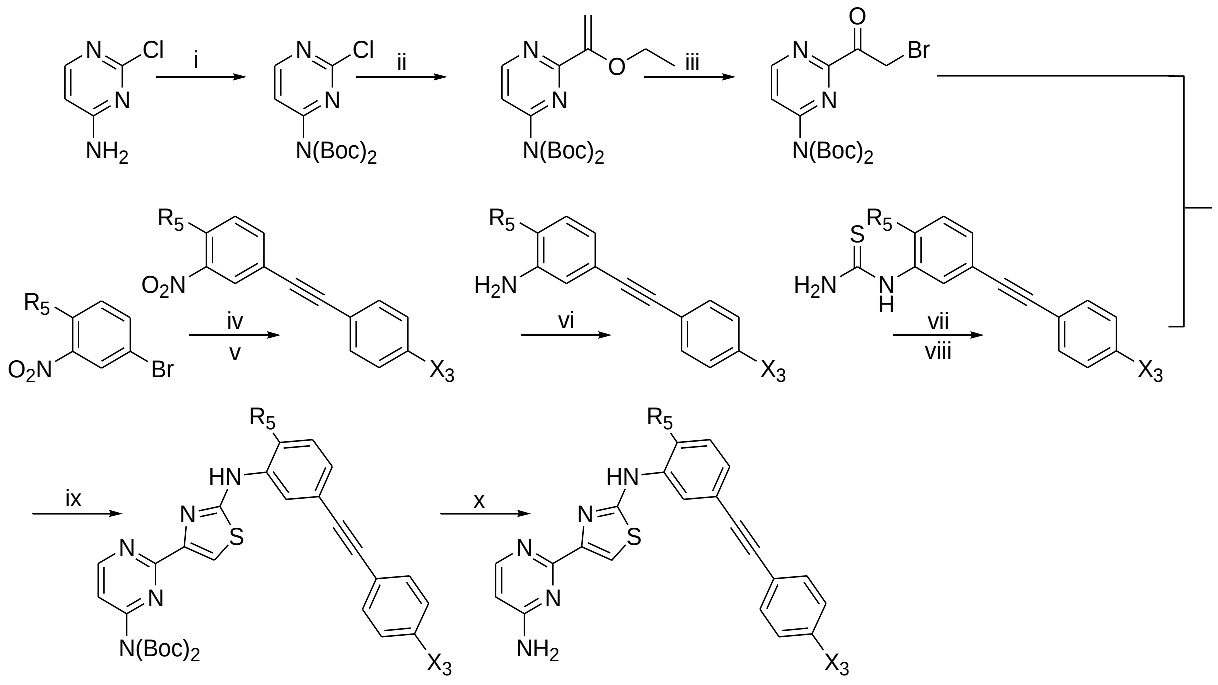

Supplementary Figure 19. Synthesis of 4-(4-aminopyrimidin-2-yl)-*N*-(3-(arylethynyl)aryl)thiazol-2-amine series. Reagents and conditions: i) Boc_2O, DMAP; ii) tributyl(1-ethoxyvinyl)tin, $Pd(PPh_3)_2Cl_2$, CsF, CuBr; iii) NBS; iv) 4-CHOAr–≡, $PdCl_2(PPh_3)_2$, CuI, PPh_3, TEA; v) amine, $NaBH(OAc)_3$, AcOH; vi) $SnCl_2.H_2O$, THF-EtOH; vii) KSCN, AcCl; viii) K_2CO_3, MeOH; ix) K_2CO_3, EtOH; ix) TFA.

4-(4-Aminopyrimidin-2-yl)-*N*-(3-(arylethynyl)aryl)thiazol-2-amine derivatives were prepared by convergent synthesis, from commercially available 4-amino-2-chloropyrimidine and appropriate bromo-3-nitrobenzene (**Supplementary Figure 19**). The 4-amino-2-chloropyrimidine was protected using Boc_2O to afford corresponding *bis*-carbamate. A Stille cross-coupling reaction with tributyl(1-ethoxyvinyl)tin gives the enol ether which was then turned into the corresponding α-bromoketone using *N*-bromosuccinimide. Starting from appropriate bromo-3-nitrobenzene, a Sonogashira cross-coupling reaction with appropriate 4-ethynylbenzaldehyde allowed to introduce the key 1,2-diarylethyne scaffold. Reductive amination afforded the corresponding benzylamine, while reduction of the nitro group followed by condensation with acetyl isothiocyanate and saponification gave the corresponding thiourea. The thiourea was then engaged in a Hantzsch thiazole synthesis with the α-bromoketone leading to the corresponding thiazole. Finally, deprotection with TFA led to expected 4-(4-aminopyrimidin-2-yl)-*N*-(3-(arylethynyl)aryl)thiazol-2-amines.

General Procedure for the Synthesis of 4-(4-Aminopyrimidin-2-yl)-*N*-(3-(arylethynyl)aryl)thiazol-2-amine Derivatives. As previously described for *N*-(3-((4-(4-aminopyrimidin-2-yl)thiazol-2-yl)amino)-4-aryl) benzamide derivatives.

4-(4-Aminopyrimidin-2-yl)-*N*-(5-((4-((dimethylamino)methyl)phenyl)ethynyl)-2-methylphenyl)thiazol-2-amine OR0237 (30%) as a white powder. ^{1}H NMR (400 MHz, $CDCl_3$) δ 8.30 (d, *J* = 5.8 Hz, 1H), 7.67 (d, *J* = 1.2 Hz, 1H), 7.60 (s, 1H), 7.50 (d, *J* = 8.1 Hz, 2H), 7.29 (d, *J* = 8.1 Hz, 2H), 7.24 (dd, *J* = 7.8, 1.2 Hz, 1H), 7.22 (d, *J* = 7.8 Hz, 1H), 6.34 (d, *J* = 5.8 Hz, 1H), 4.98 (s, 2H), 3.43 (s, 2H), 2.32 (s, 3H), 2.24 (s, 6H); ^{13}C NMR (100 MHz, $CDCl_3$) δ 166.21, 163.10, 160.53, 156.60, 150.57, 139.37, 138.76, 131.71, 131.45, 130.23, 129.24, 128.02, 123.03, 122.49, 121.96, 111.40, 103.46, 89.63, 88.85, 64.23, 45.49, 18.11; LC method (D) R_t = 4.030 min.

4-(4-Aminopyrimidin-2-yl)-*N*-(2-methyl-5-((4-((4-methylpiperazin-1-yl)methyl)phenyl)ethynyl)phenyl)thiazol-2-amine OR0153 (45%) as a white powder. ^{1}H NMR (400 MHz, $CDCl_3$) δ 8.30 (d, *J* = 5.7 Hz, 1H), 7.67 (d, *J* = 1.2 Hz, 1H), 7.60 (s, 1H), 7.48 (d, *J* = 8.0 Hz, 2H), 7.31 (d, *J* = 8.0 Hz, 2H), 7.24 (dd, *J* = 7.8, 1.2 Hz, 1H), 7.22 (d, *J* = 8.0 Hz), 6.34 (d, *J* = 5.7 Hz, 1H), 4.98 (s, 2H), 3.51 (s, 2H), 2.47 (brs, 8H), 2.32 (s, 3H), 2.29 (s, 3H); ^{13}C NMR (100 MHz, $CDCl_3$) δ 166.37, 163.13, 160.55, 156.55, 150.59, 138.84, 131.65, 131.45, 130.48, 129.24, 128.10, 123.30, 122.45, 121.91, 114.19, 111.32, 103.50, 89.60, 88.81, 62.82, 55.17, 53.10, 46.05, 18.08; LC method (D) R_t = 3.865 min.

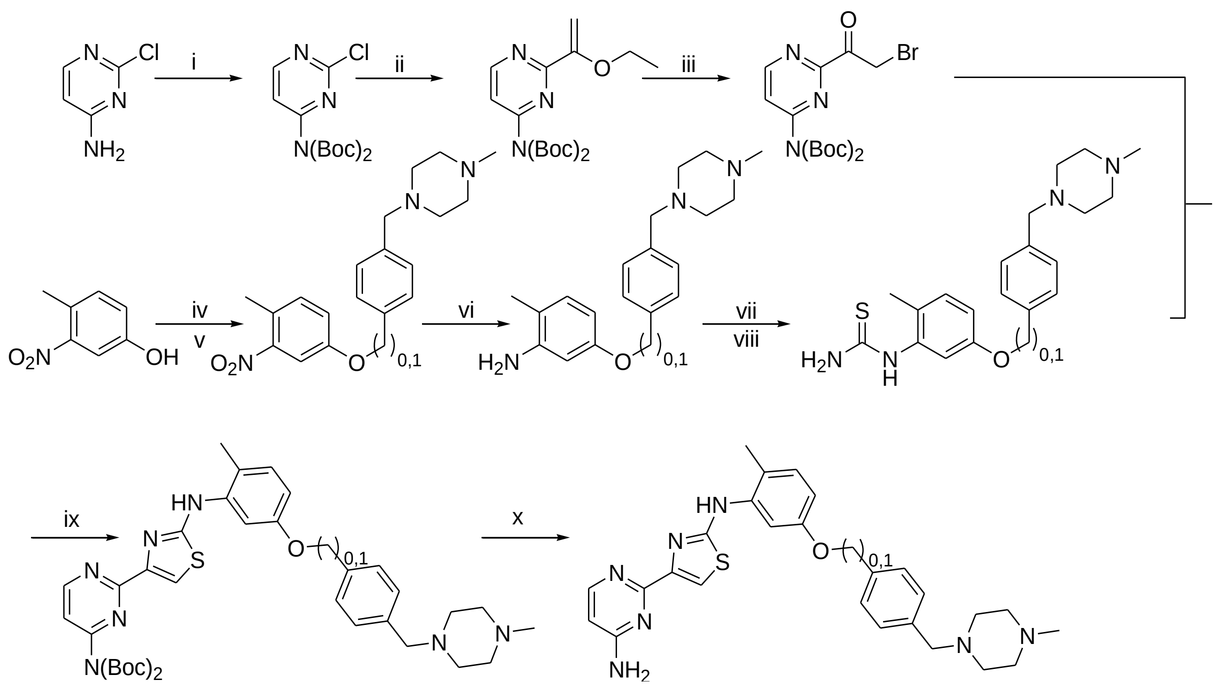

Supplementary Figure 20. Synthesis of 4-(4-aminopyrimidin-2-yl)-*N*-(2-methyl-5-(4-((4-methylpiperazin-1-yl)methyl)phenoxy)phenyl)thiazol-2-amine and 4-(4-aminopyrimidin-2-yl)-*N*-(2-methyl-5-(4-((4-methylpiperazin-1-yl)methyl)benzyloxy)phenyl)thiazol-2-amine. Reagents and conditions: i) Boc_2O, DMAP; ii) tributyl(1-ethoxyvinyl)tin, $Pd(PPh_3)_2Cl_2$, CsF, CuBr; iii) NBS; iv) (4-formylphenyl)boronic acid, $Cu(OAc)_2$, TEA; v) 1-methylpiperazine, $NaBH(OAc)_3$, AcOH; or iv-v) (4-((4-methylpiperazin-1-yl)methyl)phenyl)methanol, DIAD, PPh_3, THF; vi) $SnCl_2.H_2O$, THF-EtOH; vii) KSCN, AcCl; viii) K_2CO_3, MeOH; ix) K_2CO_3, EtOH; ix) TFA.

4-(4-Aminopyrimidin-2-yl)-*N*-(2-methyl-5-(4-((4-methylpiperazin-1-yl)methyl)phenoxy)phenyl)thiazol-2-amine and benzyloxyphenyl analogue were prepared by convergent synthesis, in ten steps, from commercially available 4-amino-2-chloropyrimidine and 4-methyl-3-nitrophenol (**Supplementary Figure 20**). The 4-amino-2-chloropyrimidine was protected using Boc_2O to afford corresponding *bis*-carbamate. A Stille cross-coupling reaction with tributyl(1-ethoxyvinyl)tin gave the enol ether which was then turned into the corresponding α-bromoketone using *N*-bromosuccinimide. Starting from 4-methyl-3-nitrophenol, *O*-arylation in the Chan-Lam coupling conditions, with (4-formylphenyl)boronic acid allowed to construct the diphenyl ether moiety while Mistunobu reaction with appropriate arylmethanol led to corresponding benzyloxyphenyl derivative. Reductive amination afforded the corresponding amine, while reduction of the nitro group followed by condensation with acetyl isothiocyanate and saponification

gave the corresponding thiourea. The thiourea was then engaged in a Hantzsch thiazole synthesis with the α-bromoketone leading to the corresponding thiazole. Finally, deprotection with TFA led to expected 4-(4-aminopyrimidin-2-yl)-*N*-(2-methyl-5-(4-((4-methylpiperazin-1-yl)methyl)phenoxy)phenyl)thiazol-2-amine and 4-(4-aminopyrimidin-2-yl)-*N*-(2-methyl-5-(4-((4-methylpiperazin-1-yl)methyl)benzyloxy)phenyl)thiazol-2-amine, respectively.

4-(4-Aminopyrimidin-2-yl)-*N*-(2-methyl-5-(4-((4-methylpiperazin-1-yl)methyl)phenoxy)phenyl)thiazol-2-amine OR0143

As previously described for *N*-(3-((4-(4-aminopyrimidin-2-yl)thiazol-2-yl)amino)-4-aryl) benzamide derivatives, (62%) as a white powder. ^{1}H NMR (400 MHz, $CDCl_3$) δ 8.29 (d, *J* = 5.7 Hz, 1H), 7.56 (s, 1H), 7.29 (d, *J* = 8.5 Hz, 2H), 7.18 (d, *J* = 2.4 Hz, 1H), 7.16 (d, *J* = 8.3 Hz, 1H), 6.98 (d, *J* = 8.5 Hz, 2H), 6.70 (dd, *J* = 8.3, 2.4 Hz, 1H), 6.32 (d, *J* = 5.7 Hz, 1H), 4.98 (brs, 2H), 3.48 (s, 2H), 2.47 (brs, 8H), 2.28 (s, 3H), 2.26 (s, 3H); ^{13}C NMR (100 MHz, $CDCl_3$) δ 165.76, 163.07, 160.44, 156.67, 156.47, 156.14, 150.46, 139.61, 133.30, 132.05, 130.67, 123.45, 118.79, 114.33, 111.23, 110.01, 103.42, 62.45, 55.21, 53.07, 46.11, 17.32; LC method (C) R_t = 3.476 min.

4-(4-Aminopyrimidin-2-yl)-*N*-(2-methyl-5-(4-((4-methylpiperazin-1-yl)methyl)benzyloxy)phenyl)thiazol-2-amine OR0232

As previously described for *N*-(3-((4-(4-aminopyrimidin-2-yl)thiazol-2-yl)amino)-4-aryl) benzamide derivatives, (70%) as a white powder. ^{1}H NMR (400 MHz, $CDCl_3$) δ 8.29 (d, *J* = 5.7 Hz, 1H), 7.56 (s, 1H), 7.37 (d, *J* = 8.1 Hz, 2H), 7.33 (d, *J* = 8.1 Hz, 2H), 7.16 (d, *J* = 2.5 Hz, 1H), 7.12 (d, *J* = 8.3 Hz, 1H), 6.69 (dd, *J* = 8.3, 2.5 Hz, 1H), 6.32 (d, *J* = 5.7 Hz, 1H), 5.04 (s, 2H), 5.00 (brs, 2H), 3.51 (s, 2H), 2.46 (brs, 8H), 2.28 (s, 3H), 2.22 (s, 3H); ^{13}C NMR (100 MHz, $CDCl_3$) δ 13C NMR (100 MHz, CDCl3) δ 166.07, 163.09, 160.50, 158.10, 156.47, 150.47, 139.35, 138.16, 135.72, 131.82, 129.52, 127.55, 121.33, 111.08, 110.84, 106.67, 103.39, 70.19, 62.81, 55.20, 53.15, 46.10, 17.14.; LC method (C) R_t = 3.679 min.

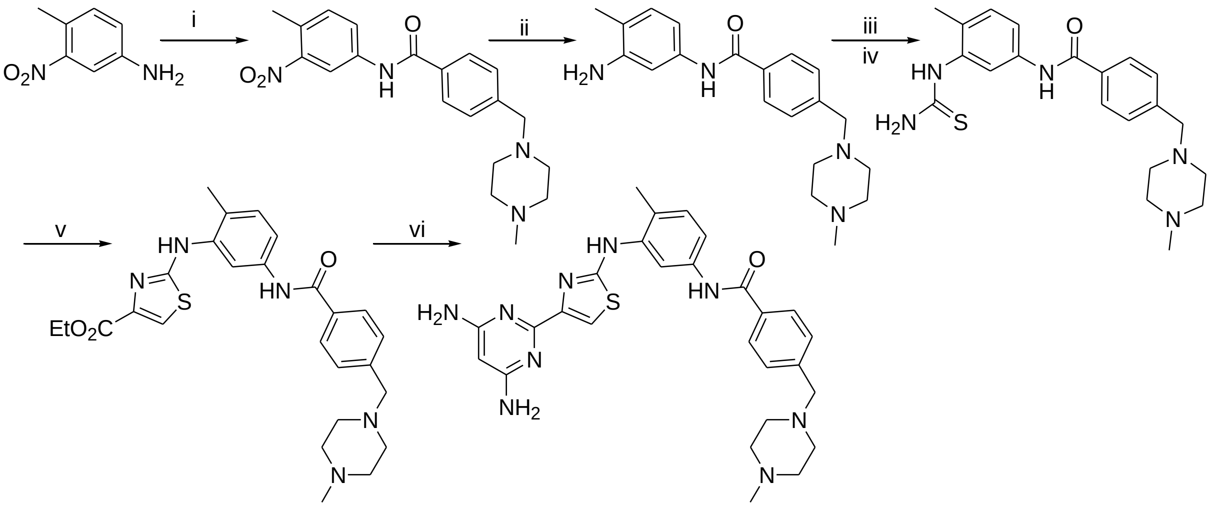

Supplementary Figure 21. Synthesis of *N*-(3-((4-(4,6-diaminopyrimidin-2-yl)thiazol-2-yl)amino)-4-methylphenyl)-4-((4-methylpiperazin-1-yl)methyl)benzamide **dCKi2**. Reagents and conditions: i) methyl 4-((4-methylpiperazin-1-yl)methyl)benzoate, Me_3Al, toluene-THF; (ii) Zn, AcOH, EtOAc; iii) AcCl, KSCN, acetone; iv) K_2CO_3, MeOH; v) ethyl bromopyruvate, EtOH; vi) malonimidamide dihydrochloride, MeONa, MeOH.

The condensation of commercially available 4-methyl-3-nitroaniline and methyl 4-((4-methylpiperazin-1-yl)methyl)benzoate was carried out in the presence of trimethylaluminum, to yield the corresponding amide quantitatively. After reduction of the nitro group, the amine was subjected to acetyli sothiocyanate, then saponification gave the corresponding thiourea. Ethyl bromopyruvate was engaged in a Hantzsch thiazole synthesis with the thiourea leading to the corresponding thiazole. Finally, tandem addition/cyclization with malonimidamide led to expected *N*-(3-((4-(4,6-diaminopyrimidin-2-yl)thiazol-2-yl)amino)-4-methylphenyl)-4-((4-methylpiperazin-1-yl)methyl)benzamide **dCKi2**.

***N*-(3-((4-(4,6-Diaminopyrimidin-2-yl)thiazol-2-yl)amino)-4-methylphenyl)-4-((4-methylpiperazin-1-yl)methyl)benzamide dCKi2**

To a suspension of ethyl 2-((2-methyl-5-(4-((4-methylpiperazin-1-yl)methyl)benzamido)phenyl)amino)thiazole-4-carboxylate (2.4 g, 4.8 mmol), malonimidamide dihydrochloride (2.5 g, 14.4 mmol) in methanol (50 mL) was added sodium methoxide solution 25 wt. % in MeOH (14.9 mL, 65.2 mmol). The reaction mixture was heated at 70 °C for 2 hours and concentrated under vacuum. The crude product was triturated with THF (3 x 50 mL) and combined organic layers were dried over Na_2SO_4. The solvent was distilled off under reduced pressure and

the residue was purified by flash chromatography, gradient DCM-MeOH-NH_4OH (100:0:0 to 90:9:1) to afford *N*-(3-((4-(4,6-diaminopyrimidin-2-yl)thiazol-2-yl)amino)-4-methylphenyl)-4-((4-methylpiperazin-1-yl)methyl)benzamide **dCKi2** (940 mg, 37%) as a light brown powder. Rf = 0.10 (DCM-MeOH-NH_4OH, 92:7:1); ^{1}H NMR (300 MHz, DMSO-d_6) δ 10.22 (brs, 1H), 9.37 (brs, 1H), 8.84 (d, *J* = 1.7 Hz, 1H), 7.90 (d, *J* = 8.2 Hz, 2H), 7.51 (dd, *J* = 8.3, 1.7 Hz, 1H), 7.42 (d, *J* = 8.2 Hz, 2H), 7.31 (s, 1H), 7.20 (d, *J* = 8.3 Hz, 1H), 6.10 (brs, 4H), 5.34 (s, 1H), 3.52 (s, 2H), 2.48-2.30 (m, 8H), 2.22 (s, 3H), 2.19 (s, 3H); ^{13}C NMR (75 MHz, DMSO-d_6) δ 165.56, 165.27, 163.66, 159.29, 151.32, 142.01, 139.60, 137.78, 133.60, 130.66, 128.59, 127.59, 125.97, 116.39, 114.39, 109.48, 81.27, 61.45, 54.46, 52.23, 45.38, 17.41; LCMS $C_{27}H_{31}N_9OS$ method (A) R_t = 6.085 min, ESI+ m/z = 530.2 (M+H).

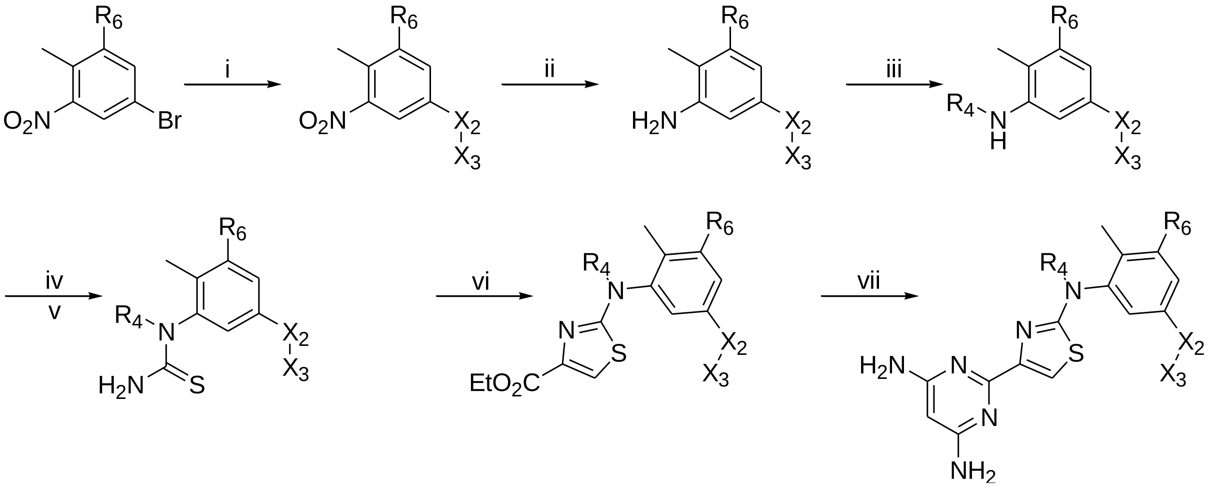

Supplementary Figure 22. Linear synthesis of *N*-([1,1'-biaryl]-3-yl)-4-(4,6-diaminopyrimidin-2-yl)thiazol-2-amine series. Reagents and conditions: i) $ArB(OR)_2$, $PdCl_2$(dppf), Na_2CO_3; ii) H_2, Pd/C, THF-EtOH; iii) RCHO, $NaBH(OAc)_3$, AcOH, THF; iv) PhCONCS, acetone v) K_2CO_3, MeOH; vi) ethyl bromopyruvate, THF; vii) malonimidamide dihydrochloride, MeONa, MeOH.

N-([1,1'-biaryl]-3-yl)-4-(4,6-diaminopyrimidin-2-yl)thiazol-2-amine derivatives were prepared in seven steps (**Supplementary Figure 22**). Starting from appropriate bromo-3-nitrobenzene, a Suzuki cross-coupling reaction with arylboronic acid derivatives allowed introducing the key biphenyl scaffold. Reduction of the nitro group successively followed by alkylation, then condensation with benzoyl isothiocyanate and saponification gave the corresponding thiourea. The ethyl bromopyruvate was engaged in a Hantzsch thiazole synthesis with the thiourea leading to the corresponding thiazole. Finally, tandem addition/cyclization with malonimidamide led to expected structurally diverse *N*-([1,1'-biaryl]-3-yl)-4-(4,6-diaminopyrimidin-2-yl)thiazol-2-amine.

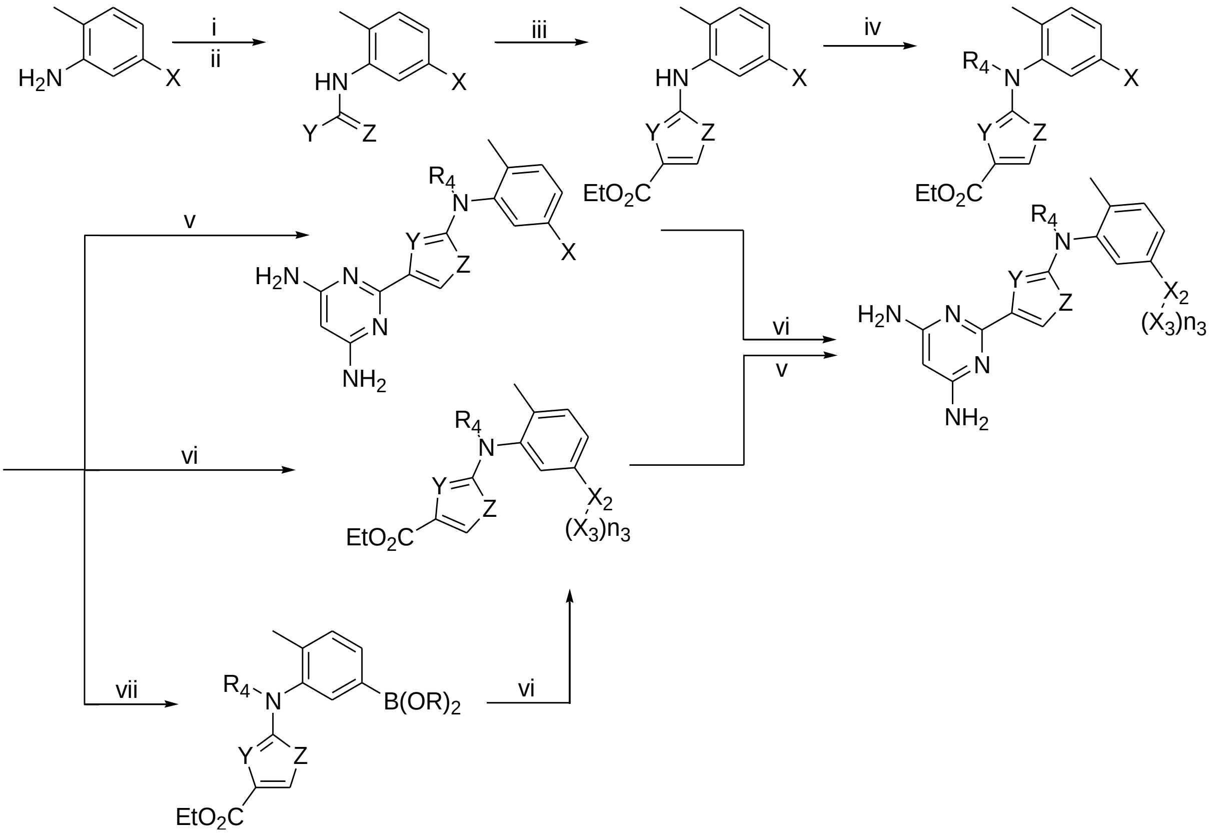

Supplementary Figure 23. Convergent synthesis of *N*-([1,1'-biaryl]-3-yl)-4-(4,6-diaminopyrimidin-2-yl)thiazol-2-amine series and analogues. Reagents and conditions: i) PhCONCS, acetone or KOCN, AcOH; ii) optionally K_2CO_3, MeOH; iii) ethyl bromopyruvate, THF; iv) R_3Br, Cs_2CO_3, DMF; v) malonimidamide dihydrochloride, MeONa, MeOH; vi) appropriate $ArB(OR)_2$ or ArX, $PdCl_2$(dppf), Na_2CO_3; vii) $(PinB)_2$, $PdCl_2$(dppf), KOAc.

Alternatively, *N*-([1,1'-biaryl]-3-yl)-4-(4,6-diaminopyrimidin-2-yl)thiazol-2-amine derivatives and analogues, were prepared by convergent approach from commercially available aminohalogenobenzene (**Supplementary Figure 23**). For instance, starting from 5-bromo- or 5-iodo-2-methylaniline, condensation with benzoyl isothiocyanate and saponification gave the corresponding thiourea. Ethyl bromopyruvate was engaged in a Hantzsch thiazole synthesis with the thiourea leading to the corresponding thiazole. The thiazole derivative was then turned into expected *N*-([1,1'-biaryl]-3-yl)-4-(4,6-diaminopyrimidin-2-yl)thiazol-2-amine by Suzuki cross-coupling reaction *via* either arylboronate or halogenoaryl precursors.

General Procedure for the Synthesis of 2-[2-(biaryl-3-ylamino)-thiazol-4-yl]-pyrimidine-4,6-diamine and analogues. Method (A): as previously described for synthesis of **dCKi-2**, briefly to a suspension of ethyl 2-([1,1'-aryl]-3-yl-amino)thiazole-4-carboxylate derivative or analogue (0.76 mmol), malonimidamide dihydrochloride (225 mg, 1.28 mmol) in methanol (8 mL) was added sodium methoxide solution 25 wt. % in MeOH (1.56 mL, 6.80 mmol). The reaction mixture was heated at 70 °C for 4 hours and concentrated under vacuum. The crude product was triturated with THF (3 x 50 mL) and combined organic layers were dried over Na_2SO_4. The solvent was distillated off under reduced pressure and the residue was purified by flash chromatography to afford the corresponding 2-[2-(biaryl-3-ylamino)-thiazol-4-yl]-pyrimidine-4,6-diamine or analogue. **Method (B)**: under argon, a suspension of appropriate 2-(2-(5-halogenophenyl)(amino)thiazol-4-yl)pyrimidine-4,6-diamine (0.214 mmol), arylboronate or arylboronic acid (0.286 mmol), $PdCl_2$(dppf) (21 mg, 0.028 mmol) and K_2CO_3 (103 mg, 0.745 mmol) in a degassed mixture of 1,4-dioxane-water (5:1, 12 mL) was heated at 80 °C for 1.5 hrs, upon complete consumption of starting material. The solvent was distillated off under reduced pressure and the residue purified by flash chromatography to afford corresponding 2-[2-(biaryl-3-ylamino)-thiazol-4-yl]-pyrimidine-4,6-diamine. **Method (C)**: from appropriate *tert*-butyl (biaryl-3-yl-[4-(4,6-diamino-pyrimidin-2-yl)-thiazol-2-yl]-aminoalkyl)carbamate, as previously described for the synthesis of *N*-(3-((4-(4-aminopyrimidin-2-yl)thiazol-2-yl)amino)-4-aryl) benzamide derivatives.

2-(2-((4-Methyl-4'-(2-(4-methylpiperazin-1-yl)ethyl)-[1,1'-biphenyl]-3-yl)(propyl)amino)thiazol-4-yl)pyrimidine-4,6-diamine OR0600 (method A, 25%) as a light brown solid. Rf = 0.25 (DCM-MeOH-NH_4OH, 90:9:1); ^{1}H NMR (400 MHz, $CDCl_3$) δ 7.52 (dd, *J* = 8.2, 2.0 Hz, 1H), 7.50 (d, *J* = 8.4 Hz, 2H), 7.45 (d, *J* = 2.0 Hz, 1H), 7.38 (d, *J* = 8.2 Hz, 1H), 7.31 (s, 1H), 7.27 (d, *J* = 8.4 Hz, 2H), 5.42 (s, 1H), 4.71 (brs, 4H), 4.01 (brs, 2H), 2.86-2.81 (m, 2H), 2.66-2.61 (m, 2H), 2.50 (brs, 8H), 2.30 (s, 3H), 2.26 (s, 3H), 1.66 (sext, *J* = 7.4 Hz, 2H), 0.92 (t, *J* = 7.4 Hz, 3H); ^{13}C NMR (100 MHz, $CDCl_3$) δ 171.22, 163.93, 160.87, 151.29, 143.34, 140.77, 139.91, 137.91, 136.29, 132.50, 129.38, 128.02, 127.00, 126.96, 110.80, 83.01, 60.52, 55.27, 53.62, 53.30, 46.20, 33.35, 21.33, 17.49, 11.42; LCMS $C_{30}H_{38}N_8S$ method (B) R_t = 4.243 min, ESI+ m/z = 543.3 (M+H).

2-(2-((4-Methyl-4'-(3-(4-methylpiperazin-1-yl)propyl)-[1,1'-biphenyl]-3-yl)(propyl)amino)thiazol-4-yl)pyrimidine-4,6-diamine OR0601 (method A, 40%) as a light yellow solid. Rf = 0.25 (DCM-MeOH-NH_4OH, 90:9:1); ^{1}H NMR (400 MHz, $CDCl_3$) δ 7.52 (dd, *J* = 8.0, 1.8 Hz, 1H), 7.49 (d, *J* = 8.1 Hz, 2H), 7.45 (d, *J* = 1.8 Hz, 1H), 7.38 (d, *J* = 8.0 Hz, 1H), 7.31 (s, 1H), 7.25 (d, *J* = 8.1 Hz, 2H), 5.42 (s, 1H), 4.82 (brs, 4H), 4.01 (brs, 2H), 2.66 (t, *J* = 7.7 Hz, 2H),

2.51 (brs, 8H), 2.44-2.38 (m, 2H), 2.31 (s, 3H), 2.25 (s, 3H), 1.85 (quint, *J* = 7.7 Hz, 2H), 1.66 (sext, *J* = 7.5 Hz, , 2H), 0.92 (t, *J* = 7.5 Hz, 3H); ^{13}C NMR (100 MHz, $CDCl_3$) δ 171.19, 163.80, 160.57, 151.07, 143.31, 141.64, 140.80, 137.61, 136.18, 132.48, 129.06, 127.93, 126.92, 110.87, 82.95, 57.95, 55.10, 53.67, 53.07, 46.02, 33.41, 28.61, 21.32, 17.49, 11.41. 1; LCMS $C_{31}H_{40}N_8S$ method (B) R_t = 4.279 min, ESI+ m/z = 557.3 (M+H).

2-(2-((2-Methyl-5-(6-(2-(4-methylpiperazin-1-yl)ethyl)pyridin-3-yl)phenyl)(propyl)amino)thiazol-4-yl)pyrimidine-4,6-diamine OR0602 (method A, 34%) as a white powder. Rf = 0.25 (DCM-MeOH-NH_4OH, 90:9:1); ^{1}H NMR (400 MHz, $CDCl_3$) δ 8.74 (d, *J* = 2.0 Hz, 1H), 7.77 (dd, *J* = 8.1, 2.0 Hz, 1H), 7.51 (dd, *J* = 7.9, 1.8 Hz, 1H), 7.44 (d, *J* = 1.8 Hz, 1H), 7.43 (d, *J* = 7.9 Hz, 1H), 7.32 (s, 1H), 7.25 (d, *J* = 8.1 Hz, 1H), 5.44 (s, 1H), 4.71 (brs, 4H), 4.01 (brs, 2H), 3.05-3.00 (m, 2H), 2.82-2.77 (m, 2H), 2.60 (brs, 4H), 2.48 (brs, 4H), 2.29 (s, 3H), 2.28 (s, 3H), 1.66 (sext, *J* = 7.4 Hz, 2H), 0.93 (t, *J* = 7.4 Hz, 3H); ^{13}C NMR (100 MHz, $CDCl_3$) δ 171.00, 163.92, 160.82, 159.60, 151.33, 147.56, 143.64, 137.64, 137.33, 134.71, 133.18, 132.87, 128.11, 126.94, 123.29, 110.86, 83.02, 58.44, 55.26, 53.65, 53.19, 46.17, 35.53, 21.36, 17.57, 11.41; LCMS $C_{29}H_{37}N_9S$ method (B) R_t = 3.952 min, ESI+ m/z = 544.3 (M+H).

2-(2-(*iso*-Butyl(4-methyl-4'-(2-(4-methylpiperazin-1-yl)ethyl)-[1,1'-biphenyl]-3-yl)amino)thiazol-4-yl)pyrimidine-4,6-diamine OR0603 (method A, 23%) as a light brown powder. Rf = 0.5 (DCM-MeOH-NH_4OH, 90:9:1); ^{1}H NMR (400 MHz, $CDCl_3$) δ 7.52 (dd, *J* = 7.6, 1.9 Hz, 1H), 7.50 (d, *J* = 1.9 Hz, 1H), 7.49 (d, *J* = 8.2 Hz, 2H), 7.38 (d, *J* = 7.6 Hz, 1H), 7.32 (s, 1H), 7.28 (d, *J* = 8.2 Hz, 2H), 5.41 (s, 1H), 4.70 (brs, 4H), 3.84 (brs, 2H), 2.88-2.80 (m, 2H), 2.67-2.61 (m, 2H), 2.56 (brs, 8H), 2.30 (s, 3H), 2.26 (s, 3H), 2.04 (sept, *J* = 6.8 Hz, 1H), 1.00 (d, *J* = 6.8 Hz, 6H). ^{13}C NMR (100 MHz, $CDCl_3$) δ 171.69, 163.92, 160.89, 151.35, 144.07, 140.59, 139.89, 138.00, 135.94, 132.71, 129.39, 127.87, 127.03, 126.81, 111.00, 83.02, 60.53, 59.79, 55.27, 53.30, 46.19, 33.35, 27.43, 20.78, 17.73; LCMS $C_{31}H_{40}N_8S$ method (B) R_t = 4.368 min, ESI+ m/z = 557.2 (M+H).

2-(2-((Cyclopropylmethyl)(4-methyl-4'-(2-(4-methylpiperazin-1-yl)ethyl)-[1,1'-biphenyl]-3-yl)amino)thiazol-4-yl)pyrimidine-4,6-diamine OR0604 (method A, 8%) as a light brown powder. Rf = 0.5 (DCM-MeOH-NH_4OH, 90:9:1); ^{1}H NMR (400 MHz, $CDCl_3$) δ 7.53 (dd, *J* = 8.7, 1.9 Hz, 1H), 7.52 (d, *J* = 1.9 Hz, 1H), 7.50 (d, *J* = 8.2 Hz, 2H), 7.38 (d, *J* = 8.7 Hz, 1H), 7.31 (s, 1H), 7.28 (d, *J* = 8.2 Hz, 2H), 5.44 (s, 1H), 4.68 (brs, 4H), 3.96 (brs, 2H), 2.89-2.80 (m, 2H), 2.68-2.60 (m, 2H), 2.53 (brs, 8H), 2.32 (s, 3H), 2.29 (s, 3H), 1.17-1.05 (m, 1H), 0.44-0.37 (m, 2H), 0.19-0.11 (m, 2H); ^{13}C

NMR (100 MHz, $CDCl_3$) δ 171.25, 163.90, 160.90, 151.20, 143.55, 140.63, 139.80, 138.01, 136.48, 132.27, 129.37, 128.44, 126.97, 110.95, 83.01, 60.48, 56.51, 55.21, 53.19, 46.12, 33.32, 17.48, 9.88, 3.97; LCMS $C_{31}H_{38}N_8S$ method (B) R_t = 4.235 min, ESI+ m/z = 555.3 (M+H).

2-(2-(*iso*-Pentyl(4-methyl-4'-(2-(4-methylpiperazin-1-yl)ethyl)-[1,1'-biphenyl]-3-yl)amino)thiazol-4-yl)pyrimidine-4,6-diamine OR0605 (method A, 28%) as a light yellow solid. Rf = 0.5 (DCM-MeOH-NH_4OH, 90:9:1); ^1H NMR (400 MHz, $CDCl_3$) δ 7.52 (dd, *J* = 7.9, 1.8 Hz, 1H), 7.50 (d, *J* = 8.2 Hz, 2H), 7.43 (d, *J* = 1.8 Hz, 1H), 7.38 (d, *J* = 7.9 Hz, 1H), 7.33 (s, 1H), 7.27 (d, *J* = 8.2 Hz, 2H), 5.45 (s, 1H), 4.85 (brs, 4H), 4.07 (brs, 2H), 2.90-2.82 (m, 2H), 2.73-2.66 (m, 2H), 2.65 (brs, 8H), 2.39 (s, 3H), 2.25 (s, 3H), 1.71-1.59 (m, 1H), 1.50 (q, *J* = 7.3 Hz, 2H), 0.90 (d, *J* = 6.6 Hz, 6H); ^{13}C NMR (100 MHz, $CDCl_3$) δ 171.26, 163.56, 143.30, 140.69, 139.52, 138.03, 136.34, 132.51, 129.38, 127.90, 127.05, 126.97, 111.15, 82.88, 60.12, 54.82, 52.55, 50.66, 45.67, 36.64, 33.12, 26.43, 22.83, 17.54; LCMS $C_{32}H_{40}N_8S$ method (B) R_t = 4.452 min, ESI+ m/z = 571.4 (M+H).

2-(2-(Butyl(4-methyl-4'-(2-(4-methylpiperazin-1-yl)ethyl)-[1,1'-biphenyl]-3-yl)amino)thiazol-4-yl)pyrimidine-4,6-diamine OR0606 (method A, 19%) as a light brown solid. Rf = 0.5 (DCM-MeOH-NH_4OH, 90:9:1); ^1H NMR (400 MHz, $CDCl_3$) δ 7.53 (dd, *J* = 8.0, 1.7 Hz, 1H), 7.50 (d, *J* = 8.2 Hz, 2H), 7.44 (d, *J* = 1.7 Hz, 1H), 7.38 (d, *J* = 8.0 Hz, 1H), 7.32 (s, 1H), 7.27 (d, *J* = 8.2 Hz, 2H), 5.45 (s, 1H), 4.82 (brs, 4H), 4.05 (brs, 2H), 2.87-2.83 (m, 2H), 2.69-2.65 (m, 2H), 2.57 (brs, 8H), 2.37 (s, 3H), 2.25 (s, 3H), 1.60-1.58 (m, 2H), 1.38-1.35 (m, 2H), 0.89 (t, *J* = 7.3 Hz, 3H); ^{13}C NMR (100 MHz, $CDCl_3$) δ 171.28, 163.65, 150.79, 143.30, 140.73, 139.64, 137.99, 136.32, 132.52, 129.38, 127.98, 127.03, 126.98, 111.06, 82.91, 60.24, 52.78, 51.86, 45.84, 33.20, 30.17, 29.84, 20.34, 17.51, 14.12; LCMS $C_{31}H_{40}N_8S$ method (B) R_t = 4.394 min, ESI+ m/z = 557.3 (M+H).

2-(2-(*iso*-Butyl(2-methyl-5-(6-(2-(4-methylpiperazin-1-yl)ethoxy)pyridin-3-yl)phenyl)amino)thiazol-4-yl)pyrimidine-4,6-diamine OR0608 (method A, 9%) as a light yellow solid. Rf = 0.5 (DCM-MeOH-NH_4OH, 90:9:1); ^1H NMR (400 MHz, $CDCl_3$) δ 8.34 (d, *J* = 2.5 Hz, 1H), 7.77 (dd, *J* = 8.6, 2.5 Hz, 1H), 7.46 (dd, *J* = 7.8, 1.7 Hz, 1H), 7.43 (d, *J* = 1.7 Hz, 1H), 7.40 (d, *J* = 7.8 Hz, 1H), 7.35 (s, 1H), 6.83 (d, *J* = 8.6 Hz, 1H), 5.50 (s, 1H), 5.04 (brs, 4H), 4.49 (t, *J* = 5.7 Hz, 2H), 3.80 (brs, 2H), 2.87 (t, *J* = 5.7 Hz, 2H), 2.80-2.60 (m, 8H), 2.43 (s, 3H), 2.26 (s, 3H), 2.10-1.96 (m, 1H), 1.00 (d, *J* = 6.6 Hz, 6H). ^{13}C NMR (100 MHz, $CDCl_3$) δ 171.42, 163.70, 163.30, 160.26, 151.03, 144.88, 144.16, 137.42, 137.33, 136.17, 132.96, 129.13, 127.42, 126.42, 111.36, 111.04, 82.90, 63.71, 59.87, 57.15, 55.06, 53.43, 46.02, 27.42, 20.67, 17.73; LCMS $C_{30}H_{39}N_9OS$ method (B) R_t = 5.303 min, ESI+ m/z = 574.3 (M+H).

***N*-((1-(3-((4-(4,6-Diaminopyrimidin-2-yl)thiazol-2-yl)(isobutyl)amino)-4-methylphenyl)-1H-1,2,3-triazol-4-yl)methyl) methanesulfonamide OR0609** (method A, 18%) as a light brown powder. Rf = 0.5 (DCM-MeOH-NH_4OH, 90:9:1); ^1H NMR (400 MHz, MeOD) δ 8.51 (s, 1H), 7.88 (d, *J* = 2.2 Hz, 1H), 7.82 (dd, *J* = 8.3, 2.2 Hz, 1H), 7.60 (d, *J* = 8.3 Hz, 1H), 7.30 (s, 1H), 5.53 (s, 1H), 4.45 (s, 2H), 3.91 (brs, 2H), 2.99 (s, 3H), 2.33 (s, 3H), 2.08-1.96 (m, 1H), 1.03 (d, *J* = 6.7 Hz, 6H); ^{13}C NMR (100 MHz, MeOD) δ 172.12, 165.62, 161.25, 152.19, 147.17, 145.86, 139.43, 137.65, 134.79, 122.65, 122.30, 121.25, 111.43, 83.02, 60.94, 40.62, 39.00, 28.74, 20.78, 17.85; LCMS $C_{22}H_{28}N_{10}O_2S_2$ method (B) R_t = 4.585 min, ESI+ m/z = 529.2 (M+H).

2-(2-((2-Aminoethyl)(4-methyl-4'-(2-(4-methylpiperazin-1-yl)ethyl)-[1,1'-biphenyl]-3-yl)amino)thiazol-4-yl) pyrimidine-4,6-diamine OR0610 (method C, 74%), as a pale yellow solid. Rf = 0.40 (DCM-MeOH-NH_4OH, 80:18:2); ^1H NMR (400 MHz, $CDCl_3$) δ 7.49-7.47 (m, 2H), 7.43 (d, *J* = 8.1 Hz, 2H), 7.31 (d, *J* = 8.5 Hz, 1H), 7.25 (s, 1H), 7.20 (d, *J* = 8.1 Hz, 2H), 5.71 (brs, 4H), 4.16 (brs, 1H), 3.80 (brs, 1H), 3.48-3.20 (m, 2H), 2.79-2.75 (m, 2H), 2.59-2.56 (m, 2H), 2.48 (brs, 8H), 2.33 (s, 3H), 2.14 (s, 3H); ^{13}C NMR (100 MHz, $CDCl_3$) δ 170.65, 163.08, 162.63, 162.29, 143.80, 141.26, 139.88, 137.34, 134.63, 132.79, 129.41, 127.59, 126.98, 118.35, 115.44, 82.75, 60.07, 58.49, 54.87, 52.69, 45.72, 40.62, 33.08, 18.56, 17.41; LCMS $C_{29}H_{37}N_9S$ method (B) R_t = 3.649 min, ESI+ m/z = 544.2 (M+H).

2-(2-((3-Aminopropyl)(4-methyl-4'-(2-(4-methylpiperazin-1-yl)ethyl)-[1,1'-biphenyl]-3-yl)amino)thiazol-4-yl) pyrimidine-4,6-diamine OR0611 (method C, 50%) as a pale yellow solid. Rf = 0.55 (DCM-MeOH-NH_4OH, 80:18:2); ^1H NMR (400 MHz, $CDCl_3$) δ 7.47 (dd, *J* = 8.1, 1.2 Hz, 1H), 7.41 (d, *J* = 8.2 Hz, 2H), 7.31 (d, *J* = 8.1 Hz, 1H), 7.30 (d, *J* = 1.2 Hz, 1H), 7.21 (d, *J* = 8.2 Hz, 2H), 7.15 (s, 1H), 5.51 (brs, 4H), 5.29 (s, 1H), 4.15 (brs, 1H), 3.81 (brs, 1H), 3.18-3.16 (m, 2H), 2.84-2.76 (m, 2H), 2.63- 2.53 (m, 2H), 2.47 (brs, 8H), 2.28 (s, 3H), 2.13 (s, 3H), 2.01-1.97 (m, 2H); ^{13}C NMR (100 MHz, $CDCl_3$) δ 171.39, 164.09, 159.70, 150.42, 143.27, 141.36, 140.09, 137.45, 135.13, 132.80, 129.41, 127.47, 127.01, 110.34, 83.57, 60.45, 55.24, 53.56, 53.26, 50.17, 46.18, 33.31, 29.84, 17.44; LCMS $C_{30}H_{39}N_9S$ method (B) R_t = 3.671 min, ESI+ m/z = 558.3 (M+H).

2-[2-({2-Methyl-5-[6-(4-methyl-piperazin-1-ylcarbonyl)-pyridin-3-yl]-phenyl}-propyl-amino)-thiazol-4-yl]-pyrimidine-4,6-diamine OR0612 (method A, 2%) as a light yellow solid. Rf = 0.5 (DCM-MeOH-NH_4OH, 90:9:1); ^1H NMR (400 MHz, $CDCl_3$) δ 8.78 (d, *J* = 2.0 Hz, 1H), 7.96 (dd, *J* = 8.1, 2.0 Hz, 1H), 7.72 (d, *J* = 8.1 Hz, 1H), 7.54 (dd, *J* = 7.9, 1.4 Hz, 1H), 7.47 (d, *J* = 1.4 Hz, 1H),

7.45 (d, *J* = 7.9 Hz, 1H), 7.35 (s, 1H), 5.53 (s, 1H), 5.26 (brs, 4H), 3.98 (brs, 2H), 3.87-3.83 (m, 2H), 3.70-3.66 (m, 2H), 2.55-2.51 (m, 2H), 2.45-2.41 (m, 2H), 2.33 (s, 3H), 2.27 (s, 3H), 1.66 (sext, *J* = 7.3 Hz, 2H), 0.93 (t, *J* = 7.3 Hz, 3H); ^{13}C NMR (100 MHz, MeOD) δ 172.11, 169.21, 164.67, 159.35, 153.49, 150.38, 147.75, 145.05, 139.21, 137.95, 136.87, 134.36, 129.30, 128.53, 124.81, 112.09, 82.43, 55.97, 55.42, 54.97, 47.99, 45.98, 43.04, 22.27, 17.63, 11.63; LCMS $C_{28}H_{33}N_9OS$ method (B) R_t = 4.071 min, ESI+ m/z = 544.3 (M+H).

2-(2-((5-(2-Aminopyrimidin-5-yl)-2-methylphenyl)(propyl)amino)thiazol-4-yl)pyrimidine-4,6-diamine OR0613 (method B, 67%) as a light brown solid. Rf = 0.45 (DCM-MeOH-NH_4OH, 95:4.5:0.5); ^{1}H NMR (400 MHz, MeOD) δ 8.54 (s, 2H), 7.54 (dd, *J* = 8.0, 1.7 Hz, 1H), 7.50 (d, *J* = 1.7 Hz, 1H), 7.47 (d, *J* = 8.0 Hz, 1H), 7.29 (s, 1H), 5.54 (s, 1H), 3.98 (brs, 2H), 2.27 (s, 3H), 1.72 (sext, *J* = 7.5 Hz, 2H), 0.97 (t, *J* = 7.5 Hz, 3H); ^{13}C NMR (100 MHz, MeOD) δ 172.11, 165.03, 163.94, 160.14, 157.35, 151.00, 144.94, 137.51, 136.42, 134.13, 127.63, 127.09, 124.06, 111.60, 82.67, 54.95, 22.24, 17.55, 11.62; LCMS $C_{21}H_{23}N_9S$ method (B) R_t = 4.620 min, ESI+ m/z = 434.2 (M+H).

2-(2-((4-Methyl-4'-((4-methylpiperazin-1-yl)sulfonyl)-[1,1'-biphenyl]-3-yl)amino)thiazol-4-yl)pyrimidine-4,6-diamine OR0614 (method B, 35%) as a light yellow solid. Rf = 0.22 (DCM-MeOH-NH_4OH, 90:9:1); ^{1}H NMR (400 MHz, $CDCl_3$) δ 7.74 (d, *J* = 8.2 Hz, 2H), 7.67 (s, 1H), 7.62 (d, *J* = 8.2 Hz, 2H), 7.40 (s, 1H), 7.23 (d, *J* = 8.5 Hz, 1H), 7.19 (d, *J* = 8.5 Hz, 1H), 5.39 (s, 1H), 5.13 (brs, 4H), 3.03-2.99 (m, 4H), 2.46-2.42 (m, 4H), 2.21 (s, 6H); ^{13}C NMR (100 MHz, $CDCl_3$) δ 166.59, 163.71, 159.74, 150.31, 145.04, 139.70, 138.20, 133.70, 131.96, 130.67, 128.45, 127.45, 123.37, 119.60, 110.58, 83.12, 67.13, 58.30, 54.04, 46.02, 45.71, 18.47, 17.66; LCMS $C_{25}H_{28}N_8O_2S_2$ method (B) R_t = 4.109 min, ESI+ m/z = 537.2 (M+H).

3'-((4-(4,6-Diaminopyrimidin-2-yl)thiazol-2-yl)(propyl)amino)-4'-methyl-[1,1'-biphenyl]-4-sulfonamide OR0615 (method B, 36%) as a light brown solid. Rf = 0.27 (DCM-MeOH-NH_4OH, 90:9:1); ^{1}H NMR (400 MHz, MeOD) δ 7.97 (d, *J* = 8.4 Hz, 2H), 7.78 (d, *J* = 8.4 Hz, 2H), 7.66 (dd, *J* = 8.1, 1.8 Hz, 1H), 7.60 (d, *J* = 1.8 Hz 1H), 7.50 (d, *J* = 8.1 Hz, 1H), 7.29 (s, 1H), 5.55 (s, 1H), 3.98 (brs, 2H), 2.28 (s, 3H), 1.71 (sext, *J* = 7.5 Hz, 2H), 0.97 (t, *J* = 7.5 Hz, 3H); ^{13}C NMR (100 MHz, MeOD) δ 172.10, 165.00, 160.09, 150.99, 144.82, 144.72, 143.93, 140.59, 138.61, 134.05, 129.19, 128.50, 128.26, 127.88, 111.66, 82.69, 54.91, 22.25, 17.60, 11.63; LCMS $C_{23}H_{25}N_7O_2S_2$ method (B) R_t = 4.933 min, ESI+ m/z = 496.2 (M+H).

***N*-(5-(3-((4-(4,6-Diaminopyrimidin-2-yl)thiazol-2-yl)(propyl)amino)-4-methylphenyl)pyridin-2-yl)-4-methylpiperazine-1-carboxamide OR0616** (method A, 19%) as a light yellow solid. Rf = 0.5 (DCM-MeOH-NH_4OH, 90:9:1); ^1H NMR (400 MHz, DMSO-d_6) δ 9.28 (brs, 1H), 8.59 (d, *J* = 2.3 Hz, 1H), 8.03 (dd, *J* = 8.8, 2.3 Hz, 1H), 7.86 (d, *J* = 8.8 Hz, 1H), 7.68 (dd, *J* = 7.9, 1.8 Hz, 1H), 7.67 (d, *J* = 1.8 Hz, 1H), 7.49 (d, *J* = 7.9 Hz, 1H), 7.17 (s, 1H), 6.03 (brs, 4H), 5.34 (s, 1H), 3.89 (brs, 2H), 3.50-3.42 (m, 4H), 2.33-2.26 (m, 4H), 2.21 (s, 3H), 2.18 (s, 3H), 1.62 (sext, *J* = 7.4 Hz, 2H), 0.90 (t, *J* = 7.4 Hz, 3H); ^{13}C NMR (151 MHz, MeOD) δ 172.09, 165.62, 161.29, 156.55, 153.85, 152.01, 146.54, 145.00, 138.73, 137.79, 137.36, 134.04, 131.66, 128.51, 127.81, 115.23, 110.97, 82.98, 55.67, 54.87, 46.07, 44.83, 22.29, 17.54, 11.61; LCMS $C_{28}H_{34}N_{10}OS$ method (B) R_t = 4.237 min, ESI+ m/z = 559.3 (M+H).

2-(2-((4-Methyl-4'-(morpholinosulfonyl)-[1,1'-biphenyl]-3-yl)(propyl)amino)thiazol-4-yl)pyrimidine-4,6-diamine OR0617 (method B, 61%) as a light yellow solid. Rf = 0.50 (DCM-MeOH-NH_4OH, 90:9:1); ^1H NMR (400 MHz, MeOD) δ 7.90 (d, *J* = 8.5 Hz, 2H), 7.85 (d, *J* = 8.5 Hz, 2H), 7.71 (dd, *J* = 8.0, 1.7 Hz, 1H), 7.67 (d, *J* = 1.7 Hz, 1H), 7.54 (d, *J* = 8.0 Hz, 1H), 7.26 (s, 1H), 5.54 (s, 1H), 4.02 (brs, 2H), 3.73-3.69 (m, 4H), 3.02-2.98 (m, 4H), 2.31 (s, 3H), 1.63 (sext, *J* = 7.3 Hz, 2H), 0.99 (t, *J* = 7.3 Hz, 3H); ^{13}C NMR (100 MHz, MeOD) δ 172.00, 165.60, 161.21, 152.04, 145.94, 145.04, 140.32, 139.04, 135.37, 134.13, 129.78, 129.46, 128.61, 128.55, 111.03, 82.95, 67.23, 54.90, 47.46, 22.31, 17.62, 11.61; LCMS $C_{27}H_{31}N_7O_3S_2$ method (B) R_t = 5.538 min, ESI+ m/z = 566.2 (M+H).

3'-((4-(4,6-Diaminopyrimidin-2-yl)thiazol-2-yl)(propyl)amino)-N,N,4'-trimethyl-[1,1'-biphenyl]-4-sulfonamide OR0618 (method B, 67%) as a light brown solid. Rf = 0.55 (DCM-MeOH-NH_4OH, 90:9:1); ^1H NMR (400 MHz, MeOD) δ 7.88 (d, *J* = 8.7 Hz, 2H), 7.85 (d, *J* = 8.7 Hz, 2H), 7.71 (dd, *J* = 7.9, 1.4 Hz, 1H), 7.66 (*J* = 1.4 Hz, 1H), 7.53 (*J* = 7.9 Hz, 1H), 7.25 (s, 1H), 5.54 (s, 1H), 4.02 (brs, 2H), 2.71 (s, 6H), 2.31 (s, 3H), 1.74 (sext, *J* = 7.4 Hz, 2H), 0.99 (t, *J* = 7.4 Hz, 3H). ^{13}C NMR (100 MHz, MeOD) δ 172.00, 165.57, 161.16, 151.99, 145.63, 145.01, 140.38, 138.95, 135.44, 134.11, 132.26, 129.65, 129.41, 128.51, 111.05, 82.94, 54.88, 38.30, 22.30, 17.61, 11.61; LCMS $C_{25}H_{29}N_7O_2S_2$ method (B) R_t = 5.395 min, ESI+ m/z = 524.2 (M+H).

2-(2-((4'-((Dimethylamino)methyl)-4-methyl-[1,1'-biphenyl]-3-yl)(propyl)amino)thiazol-4-yl)pyrimidine-4,6-diamine OR0619 (method B, 61%) as a light yellow solid. Rf = 0.50 (DCM-MeOH-NH_4OH, 90:9:1); ^1H NMR (400 MHz, MeOD) δ 7.63 (dd, *J* = 8.0, 1.8 Hz, 1H), 7.60 (d, *J* = 8.1 Hz, 2H), 7.54 (d, *J* = 1.8 Hz, 1H), 7.47 (d, *J* = 8.0 Hz, 1H), 7.40 (d, *J* = 8.1 Hz, 2H), 7.23 (s,

1H), 5.54 (s, 1H), 4.01 (brs, 2H), 3.51 (s, 2H), 2.28 (s, 3H), 2.26 (s, 6H), 1.73 (sext, *J* = 7.4 Hz, 2H), 0.99 (t, *J* = 7.4 Hz, 3H); ^{13}C NMR (100 MHz, MeOD) δ 172.18, 165.64, 161.34, 152.03, 144.75, 141.99, 140.42, 138.13, 137.33, 133.79, 131.38, 128.89, 128.18, 127.75, 110.91, 82.96, 64.53, 54.76, 45.22, 22.30, 17.50, 11.61; LCMS $C_{26}H_{31}N_7S$ method (B) R_t = 4.385 min, ESI+ m/z = 474.3 (M+H).

2-(2-((2-Methyl-5-(pyridin-3-yl)phenyl)(propyl)amino)thiazol-4-yl)pyrimidine-4,6-diamine OR0620 (method B, 86%) as a light brown solid. Rf = 0.50 (DCM-MeOH-NH_4OH, 90:9:1); ^{1}H NMR (400 MHz, MeOD) δ 8.81 (d, *J* = 1.8 Hz, 1H), 8.52 (dd, *J* = 4.9, 1.8 Hz, 1H), 8.10 (dt, *J* = 8.0, 1.8 Hz, 1H), 7.67 (dd, *J* = 7.8, 1.9 Hz, 1H), 7.63 (d, *J* = 1.9 Hz, 1H), 7.55 (d, *J* = 7.8 Hz, 1H), 7.52 (dd, *J* = 8.0, 4.9 Hz, 1H), 7.25 (s, 1H), 5.54 (s, 1H), 4.01 (brs, 2H), 2.31 (s, 3H), 1.74 (sext, *J* = 7.4 Hz, 2H), 0.99 (t, *J* = 7.4 Hz, 3H); ^{13}C NMR (100 MHz, MeOD) δ 171.98, 165.61, 161.25, 152.05, 149.00, 148.19, 145.12, 138.79, 138.49, 137.44, 136.37, 134.21, 129.17, 128.33, 125.57, 111.01, 82.96, 54.92, 22.30, 17.61, 11.60; LCMS $C_{22}H_{23}N_7S$ method (B) R_t = 4.544 min, ESI+ m/z = 418.2 (M+H).

(5-(3-((4-(4,6-Diaminopyrimidin-2-yl)thiazol-2-yl)(propyl)amino)-4-methylphenyl)pyridin-2-yl)(morpholino) methanone OR0621 (method B, 71%) as a light yellow solid. Rf = 0.50 (DCM-MeOH-NH_4OH, 90:9:1); ^{1}H NMR (400 MHz, MeOD) δ 8.88 (d, *J* = 2.0 Hz, 1H), 8.21 (dd, *J* = 8.2, 2.0 Hz, 1H), 7.73 (dd, *J* = 8.0, 1.8 Hz, 1H), 7.72 (d, *J* = 8.2 Hz, 1H), 7.69 (d, *J* = 1.8 Hz, 1H), 7.56 (d, *J* = 8.0 Hz, 1H), 7.25 (s, 1H), 5.53 (s, 1H), 4.02 (brs, 2H), 3.79 (brs, 4H), 3.70-3.64 (m, 2H), 3.62-3.56 (m, 2H), 2.32 (s, 3H), 1.74 (sext, *J* = 7.4 Hz, 2H), 0.99 (t, *J* = 7.4 Hz, 3H); ^{13}C NMR (100 MHz, MeOD) δ 171.91, 169.24, 165.61, 161.24, 153.30, 152.08, 147.72, 145.20, 139.29, 138.03, 137.87, 136.88, 134.30, 129.36, 128.41, 124.96, 111.02, 82.97, 67.93, 54.94, 44.00, 22.30, 17.65, 11.60; LCMS $C_{27}H_{30}N_8O_2S$ method (B) R_t = 4.835 min, ESI+ m/z = 531.2 (M+H).

Methyl 5-(3-((4-(4,6-diaminopyrimidin-2-yl)thiazol-2-yl)(propyl)amino)-4-methylphenyl)picolinate OR0622 (method B, 42%) as a light brown solid. Rf = 0.50 (DCM-MeOH-NH_4OH, 90:9:1); ^{1}H NMR (400 MHz, MeOD) δ 8.95 (dd, *J* = 2.0, 0.6 Hz, 1H), 8.27 (dd, *J* = 8.2, 2.0 Hz, 1H), 8.22 (dd, *J* = 8.2, 0.6 Hz, 1H), 7.75 (dd, *J* = 8.0, 1.8 Hz, 1H), 7.73 (d, *J* = 1.8 Hz, 1H), 7.58 (d, *J* = 8.0 Hz, 1H), 7.26 (s, 1H), 5.54 (s, 1H), 4.02 (brs, 2H), 4.00 (s, 3H) 2.33 (s, 3H), 1.74 (sext, *J* = 7.4 Hz, 2H), 0.99 (t, *J* = 7.4 Hz, 3H). ; ^{13}C NMR (100 MHz, MeOD) δ 171.88, 166.44, 165.59, 161.19, 152.06, 148.65, 147.36, 145.28, 140.31, 139.80, 137.46, 136.88, 134.38, 129.52,

128.54, 126.61, 111.07, 82.96, 54.99, 53.24, 22.30, 17.69, 11.60; LCMS $C_{24}H_{25}N_7O_2S$ method (B) R_t = 5.062 min, ESI+ m/z = 476.3 (M+H).

***N*-(5-(3-((4-(4,6-Diaminopyrimidin-2-yl)thiazol-2-yl)(propyl)amino)-4-methylphenyl)pyridin-2-yl)-4-methylpiperazine-1-sulfonamide OR0625** (method A, 18%) as a white powder. Rf = 0.5 (DCM-MeOH-NH_4OH, 90:9:1); ^{1}H NMR (400 MHz, DMSO-d_6) ^{1}H NMR (400 MHz, DMSO-d_6) δ 8.51 (d, *J* = 2.0 Hz, 1H), 8.12 (dd, *J* = 8.8, 2.0 Hz, 1H), 7.71 (dd, *J* = 8.4, 1.8 Hz, 1H), 7.70 (d, *J* = 1.8 Hz, 1H), 7.52 (d, *J* = 8.8 Hz, 1H), 7.41 (s, 1H), 7.21 (d, *J* = 8.4 Hz, 1H), 7.03 (s, 4H), 5.48 (s, 1H), 3.97 (brs, 2H), 3.25-3.21 (m, 4H), 2.56-2.52 (m, 4H), 2.28 (s, 3H), 2.22 (s, 3H), 1.62 (sext, *J* = 7.4 Hz, 2H), 0.93 (t, *J* = 7.4 Hz, 3H); ^{13}C NMR (151 MHz, MeOD) δ 172.68, 162.12, 154.35, 153.90, 145.74, 144.50, 139.03, 138.20, 137.86, 134.31, 133.06, 129.85, 128.22, 128.08, 115.18, 114.92, 80.98, 55.11, 54.99, 46.95, 45.60, 22.19, 17.48, 11.73; LCMS $C_{27}H_{34}N_{10}O_2S_2$ method (B) R_t = 4.182 min, ESI+ m/z = 595.3 (M+H).

2-(2-((5-(*1H*-Indol-5-yl)-2-methylphenyl)(propyl)amino)thiazol-4-yl)pyrimidine-4,6-diamine OR0626 (method B, 82%) as a light yellow solid. Rf = 0.50 (DCM-MeOH-NH_4OH, 90:9:1); ^{1}H NMR (400 MHz, MeOD) δ 7.78 (d, *J* = 1.8 Hz, 1H), 7.63 (dd, *J* = 7.9, 1.8 Hz, 1H), 7.53 (d, *J* = 1.6 Hz, 1H), 7.44 (d, *J* = 7.9 Hz, 1H), 7.43 (d, *J* = 8.2 Hz, 1H), 7.36 (dd, *J* = 8.2, 1.6 Hz, 1H), 7.25 (s, 1H), 7.24 (d, *J* = 2.8 Hz, 1H), 6.50 (d, *J* = 2.8 Hz, 1H), 5.54 (s, 1H), 4.00 (brs, 2H), 2.27 (s, 3H), 1.75 (sext, *J* = 7.4 Hz, 2H), 0.99 (t, *J* = 7.4 Hz, 3H); ^{13}C NMR (100 MHz, MeOD) δ 172.44, 165.37, 160.86, 151.48, 144.48, 144.22, 137.37, 135.56, 133.49, 132.36, 130.07, 128.85, 128.40, 126.44, 121.60, 119.39, 112.58, 111.16, 102.85, 82.81, 58.32, 54.72, 22.30, 18.36, 17.40, 11.64; LCMS $C_{25}H_{25}N_7S$ method (B) R_t = 5.452 min, ESI+ m/z = 456.2 (M+H).

2-(2-((2-Methyl-5-(6-(2-(4-methylpiperazin-1-yl)ethoxy)pyridin-3-yl)phenyl)(propyl)amino)thiazol-4-yl)pyrimidine-4,6-diamine OR0629 (method A, 31%) as a white powder. Rf = 0.5 (DCM-MeOH-NH_4OH, 90:9:1); ^{1}H NMR (400 MHz, $CDCl_3$) δ 8.34 (d, *J* = 2.3 Hz, 1H), 7.75 (dd, *J* = 8.6, 2.3 Hz, 1H), 7.45 (dd, *J* = 7.9, 1.8 Hz, 1H), 7.39 (d, *J* = 7.9 Hz, 1H), 7.38 (d, *J* = 1.8 Hz, 1H), 7.31 (s, 1H), 6.81 (d, *J* = 8.6 Hz, 1H), 5.42 (s, 1H), 4.89 (brs, 4H), 4.47 (t, *J* = 5.8 Hz, 2H), 3.98 (brs, 2H), 2.82 (t, *J* = 5.8 Hz, 2H), 2.64 (brs, 4H), 2.51 (brs, 4H), 2.30 (s, 3H), 2.25 (s, 3H), 1.65 (sext, *J* = 7.4 Hz, 2H), 0.91 (t, *J* = 7.4 Hz, 3H); ^{13}C NMR (100 MHz, $CDCl_3$) δ 170.95, 163.76, 163.31, 160.39, 151.04, 144.88, 143.52, 137.64, 137.33, 136.53, 132.76, 129.09, 127.63, 126.59, 111.33, 110.87, 82.92, 63.71, 57.15, 55.05, 53.72, 53.40, 46.01, 21.32, 17.50, 11.39; LCMS $C_{29}H_{37}N_9OS$ method (B) R_t = 4.379 min, ESI+ m/z = 560.3 (M+H).

2-((5-(3-((4-(4,6-Diaminopyrimidin-2-yl)thiazol-2-yl)(propyl)amino)-4-methylphenyl)pyridin-2-yl)oxy)-1-(4-methylpiperazin-1-yl)ethan-1-one OR0630 (method A, 3%) as a white powder. Rf = 0.5 (DCM-MeOH-NH_4OH, 90:9:1); ^1H NMR (400 MHz, $CDCl_3$) δ 8.30 (d, *J* = 2.4 Hz, 1H), 7.80 (dd, *J* = 8.6, 2.4 Hz, 1H), 7.45 (dd, *J* = 7.9, 1.7 Hz, 1H), 7.38 (d, *J* = 7.9 Hz, 1H), 7.37 (d, *J* = 1.7 Hz, 1H), 7.36 (s, 1H), 6.96 (d, *J* = 8.6 Hz, 1H), 5.56 (s, 1H), 5.47 (brs, 4H), 5.05 (s, 2H), 3.97 (brs, 2H), 3.70-3.62 (m, 2H), 3.58-3.50 (m, 2H), 2.51-2.43 (m, 2H), 2.45-2.37 (m, 2H), 2.32 (s, 3H), 2.24 (s, 3H), 1.65 (sext, *J* = 7.4 Hz, 2H), 0.93 (t, *J* = 7.4 Hz, 3H); ^{13}C NMR (151 MHz, MeOD) δ 172.15, 169.25, 165.33, 163.71, 160.70, 151.48, 145.40, 144.90, 138.95, 138.85, 137.60, 134.02, 131.10, 128.59, 127.93, 112.27, 111.30, 82.82, 64.10, 55.74, 55.40, 54.86, 46.04, 45.41, 42.65, 22.28, 17.52, 11.62; LCMS $C_{29}H_{35}N_9O_2S$ method (B) R_t = 4.361 min, ESI+ m/z = 574.3 (M+H).

2-(2-(Methyl(4-methyl-4'-((4-methylpiperazin-1-yl)sulfonyl)-[1,1'-biphenyl]-3-yl)amino)thiazol-4-yl)pyrimidine-4,6-diamine OR0631 (method B, 67%) as a light yellow solid. Rf = 0.47 (DCM-MeOH-NH_4OH, 90:9:1); ^1H NMR (400 MHz, MeOD) δ 7.89 (d, *J* = 8.6 Hz, 2H), 7.84 (d, *J* = 8.6 Hz, 2H), 7.71 (d, *J* = 1.9 Hz, 1H), 7.69 (dd, *J* = 7.7, 1.9 Hz, 1H), 7.53 (d, *J* = 7.7 Hz, 1H), 7.29 (s, 1H), 5.54 (s, 1H), 3.60 (s, 3H), 3.09-3.01 (m, 4H), 2.54-2.46 (m, 4H), 2.32 (s, 3H), 2.25 (s, 3H); ^{13}C NMR (100 MHz, $CDCl_3$) δ 171.88, 165.64, 161.18, 152.13, 146.67, 145.82, 140.49, 138.39, 135.59, 134.02, 129.67, 128.56, 128.49, 128.31, 111.39, 82.98, 55.08, 46.95, 45.75, 40.15, 17.37; LCMS $C_{26}H_{30}N_8O_2S_2$ method (B) R_t = 4.294 min, ESI+ m/z = 551.2 (M+H).

2-(2-(Ethyl(4-methyl-4'-((4-methylpiperazin-1-yl)sulfonyl)-[1,1'-biphenyl]-3-yl)amino)thiazol-4-yl)pyrimidine-4,6-di-amine OR0632 (method B, 58%) as a light brown solid. Rf = 0.36 (DCM-MeOH-NH_4OH, 90:9:1); ^1H NMR (400 MHz, MeOD) δ 7.89 (d, *J* = 8.6 Hz, 2H), 7.84 (d, *J* = 8.6 Hz, 2H), 7.71 (dd, *J* = 8.0, 1.8 Hz, 1H), 7.66 (d, *J* = 1.8 Hz, 1H), 7.54 (d, *J* = 8.0 Hz, 1H), 7.26 (s, 1H), 5.54 (s, 1H), 4.13 (brs, 2H), 3.09-3.01 (m, 4H), 2.54-2.46 (m, 4H), 2.31 (s, 3H), 2.25 (s, 3H), 1.28 (t, *J* = 7.2 Hz, 3H); ^{13}C NMR (100 MHz, MeOD) δ 171.66, 165.65, 161.30, 152.12, 145.84, 144.65, 140.31, 139.18, 135.58, 134.06, 129.70, 129.56, 128.58, 128.55, 110.99, 82.98, 55.08, 47.75, 46.95, 45.75, 17.58, 13.55, LCMS $C_{27}H_{32}N_8O_2S_2$ method (B) R_t = 4.418 min, ESI+ m/z = 565.2 (M+H).

2-(2-((5-Fluoro-4-methyl-4'-((4-methylpiperazin-1-yl)sulfonyl)-[1,1'-biphenyl]-3-yl)(propyl)amino)thiazol-4-yl) pyrimidine-4,6-diamine OR0633 (method A, 11%) as a light brown solid. Rf = 0.5 (DCM-MeOH-NH_4OH, 90:9:1); ^1H NMR (400 MHz, $CDCl_3$) δ 7.81 (d, *J* = 8.4

Hz, 2H), 7.68 (d, *J* = 8.4 Hz, 2H), 7.40 (s, 1H), 7.32 (dd, *J* = 10.2, 1.6 Hz, 1H), 7.31 (d, *J* = 1.6 Hz, 1H), 5.57 (s, 1H), 5.40 (brs, 4H), 4.01 (brs, 2H), 3.10-3.02 (m, 4H), 2.53-2.45 (m, 4H), 2.26 (s, 3H), 2.19 (s, 3H), 1.66 (sext, *J* = 7.4 Hz, 2H), 0.95 (t, *J* = 7.4 Hz, 3H); ^{19}F NMR (376 MHz, $CDCl_3$) δ -110.83; ^{13}C NMR (100 MHz, $CDCl_3$) δ ^{13}C 170.53, 162.78 (d, *J* = 247.5 Hz), 162.51, 145.01 (d, *J* = 6.8 Hz), 143.52, 139.57 (d, *J* = 9.6 Hz), 134.70, 128.70, 127.55, 125.60 (d, *J* = 17.5 Hz), 123.93, 114.16, 113.93, 112.22, 82.54, 54.23, 54.13, 46.10, 45.80, 21.31, 11.42, 10.18; LCMS $C_{28}H_{33}FN_8O_2S_2$ method (B) R_t = 4.384 min, ESI+ m/z = 597.2 (M+H).

2-(2-((3'-Methoxy-4-methyl-4'-((4-methylpiperazin-1-yl)sulfonyl)-[1,1'-biphenyl]-3-yl)(propyl)amino)thiazol-4-yl) pyrimidine-4,6-diamine OR0635 (method A, 36%) as light brown powder. Rf = 0.5 (DCM-MeOH-NH_4OH, 90:9:1); ^{1}H NMR (400 MHz, $CDCl_3$) δ 7.91 (d, *J* = 8.1 Hz, 1H), 7.53 (dd, *J* = 8.1, 1.9 Hz, 1H), 7.45 (d, *J* = 1.9 Hz, 1H), 7.43 (d, *J* = 8.2 Hz, 1H), 7.35 (s, 1H), 7.20 (dd, *J* = 8.2, 1.4 Hz, 1H), 7.12 (d, *J* = 1.4 Hz, 1H), 5.49 (s, 1H), 5.10 (brs, 4H), 3.99 (brs, 2H), 3.96 (s, 3H), 3.31-3.22 (m, 4H), 2.50-2.42 (m, 4H), 2.29 (s, 3H), 2.28 (s, 3H), 1.66 (sext, *J* = 7.4 Hz, 2H), 0.94 (t, *J* = 7.4 Hz, 3H); ^{13}C NMR (100 MHz, $CDCl_3$) δ 170.91, 163.31, 159.37, 157.46, 150.25, 146.51, 143.59, 139.34, 138.09, 132.85, 132.48, 128.36, 127.30, 125.02, 119.05, 111.33, 110.73, 82.80, 56.20, 54.84, 53.94, 46.02, 45.98, 21.34, 17.64, 11.42; LCMS $C_{29}H_{36}FN_8O_3S_2$ method (B) R_t = 4.258 min, ESI+ m/z = 609.3 (M+H).

2-(2-(*iso*-Propyl(4-methyl-4'-((4-methylpiperazin-1-yl)sulfonyl)-[1,1'-biphenyl]-3-yl)amino)thiazol-4-yl)pyrimidine-4,6-diamine OR0636 (method B, 29%) as a light brown solid. Rf = 0.41 (DCM-MeOH-NH_4OH, 90:9:1); ^{1}H NMR (400 MHz, MeOD) δ 7.88 (d, *J* = 8.0 Hz, 2H), 7.84 (d, *J* = 8.0 Hz, 2H), 7.73 (dd, *J* = 8.0, 1.9 Hz, 1H), 7.57 (d, *J* = 1.9 Hz, 1H), 7.55 (d, *J* = 8.0 Hz, 1H), 7.24 (s, 1H), 5.54 (s, 1H), 5.21 (sept, *J* = 6.7 Hz, 1H), 3.09-3.01 (m, 4H), 2.54-2.46 (m, 4H), 2.32 (s, 3H), 2.25 (s, 3H), 1.29-1.28 (m, 6H); ^{13}C NMR (100 MHz, MeOD) δ 171.77, 165.62, 161.37, 152.12, 145.82, 142.12, 140.70, 140.05, 135.57, 134.11, 130.95, 129.73, 128.86, 128.57, 110.77, 82.97, 55.07, 52.86, 46.94, 45.75, 21.46, 18.24; LCMS $C_{28}H_{34}FN_8O_2S_2$ method (B) R_t = 4.311 min, ESI+ m/z = 579.3 (M+H).

2-(2-(*iso*-Butyl (4-methyl-4'-((4-methylpiperazin-1-yl)sulfonyl)-[1,1'-biphenyl]-3-yl)amino)thiazol-4-yl)pyrimidine-4,6-diamine OR0637 (method B, 70%) as a light brown solid. Rf = 0.45 (DCM-MeOH-NH_4OH, 90:9:1); ^{1}H NMR (400 MHz, MeOD) δ 7.88 (d, *J* = 8.8 Hz, 2H), 7.85 (d, *J* = 8.8 Hz, 2H), 7.69 (dd, *J* = 8.0, 1.7 Hz, 1H), 7.67 (d, *J* = 1.7 Hz, 1H), 7.53 (d, *J* = 8.0 Hz, 1H), 7.26 (s, 1H), 5.53 (s, 1H), 3.92 (brs, 2H), 3.09-3.01 (m, 4H), 2.54-2.46 (m, 4H), 2.31 (s,

3H), 2.25 (s, 3H), 2.06-1.96 (m, 1H), 1.03 (d, *J* = 6.6 Hz, 6H); ^{13}C NMR (100 MHz, MeOD) δ 172.66, 165.63, 161.33, 152.13, 145.90, 145.49, 140.15, 138.81, 135.57, 134.36, 129.73, 129.26, 128.58, 128.41, 111.14, 83.00, 60.51, 55.08, 46.95, 45.75, 28.72, 20.82, 17.80; LCMS $C_{29}H_{36}FN_8O_2S_2$ method (B) R_t = 4.369 min, ESI+ m/z = 593.3 (M+H).

2-(2-((4'-((4-Ethylpiperazin-1-yl)sulfonyl)-4-methyl-[1,1'-biphenyl]-3-yl)(propyl)amino)thiazol-4-yl)pyrimidine-4,6-diamine OR0638 (method A, 36%) as a white solid. Rf = 0.5 (DCM-MeOH-NH_4OH, 90:9:1); ^{1}H NMR (400 MHz, $CDCl_3$) δ 7.80 (d, *J* = 8.5 Hz, 2H), 7.69 (d, *J* = 8.5 Hz, 2H), 7.53 (dd, *J* = 7.9, 1.9 Hz, 1H), 7.45 (d, *J* = 1.9 Hz, 1H), 7.44 (d, *J* = 7.9 Hz, 1H), 7.35 (s, 1H), 5.49 (s, 1H), 5.09 (brs, 4H), 4.00 (brs, 2H), 3.10-3.02 (m, 4H), 2.57-2.49 (m, 4H), 2.39 (q, *J* = 7.2 Hz, 2H), 2.28 (s, 3H), 1.67 (sext, *J* = 7.4 Hz, 2H), 1.02 (t, *J* = 7.2 Hz, 3H), 0.94 (t, *J* = 7.4 Hz, 3H); ^{13}C NMR (100 MHz, $CDCl_3$) δ 170.91, 163.32, 159.40, 150.31, 144.63, 143.58, 139.05, 138.01, 133.88, 132.96, 128.63, 128.55, 127.56, 127.30, 111.30, 82.81, 53.83, 52.02, 51.91, 46.23, 21.35, 17.61, 12.03, 11.42; LCMS $C_{29}H_{36}FN_8O_2S_2$ method (B) R_t = 4.316 min, ESI+ m/z = 593.3 (M+H).

2-(2-((4-Methyl-4'-(piperazin-1-ylsulfonyl)-[1,1'-biphenyl]-3-yl)(propyl)amino)thiazol-4-yl)pyrimidine-4,6-diamine OR0639 (method C, 53%) as a white solid. Rf = 0.18 (DCM-MeOH-NH_4OH, 90:9:1); ^{1}H NMR (400 MHz, $CDCl_3$) δ 7.83 (d, *J* = 8.2 Hz, 2H), 7.74 (d, *J* = 8.2 Hz, 2H), 7.57 (dd, *J* = 7.9, 1.9 Hz, 1H), 7.51 (d, *J* = 1.9 Hz, 1H), 7.47 (d, *J* = 7.9 Hz, 1H), 7.38 (s, 1H), 5.50 (s, 1H), 5.00 (brs, 4H), 4.01 (brs, 2H), 3.10-3.02 (m, 4H), 3.01-2.94 (m, 4H), 2.30 (s, 3H), 1.69 (sext, *J* = 7.2 Hz, 2H), 0.96 (t, *J* = 7.2 Hz, 3H); ^{13}C NMR (100 MHz, $CDCl_3$) δ 179.89, 170.95, 163.53, 159.84, 150.64, 144.57, 143.59, 139.00, 138.05, 134.28, 132.93, 128.59, 128.52, 127.57, 127.28, 111.18, 82.92, 53.89, 46.84, 45.32, 35.43, 21.34, 17.63, 11.43; LCMS $C_{27}H_{32}N_8O_2S_2$ method (B) R_t = 4.272 min, ESI+ m/z = 565.3 (M+H).

2-(2-((4-Methyl-4'-((4-methylpiperazin-1-yl)sulfonyl)-[1,1'-biphenyl]-3-yl)(propyl)amino)oxazol-4-yl)pyrimidine-4,6-diamine OR0640 (method A, 34%) as a light brown solid. Rf = 0.50 (DCM-MeOH-NH_4OH, 90:10:1); ^{1}H NMR (400 MHz, MeOD) δ 7.86 (d, *J* = 8.4 Hz, 2H), 7.83 (s, 1H), 7.82 (d, *J* = 8.4 Hz, 2H), 7.61 (d, *J* = 1.6 Hz, 1H), 7.59 (dd, *J* = 8.4, 1.6 Hz, 1H), 7.45 (d, *J* = 8.4 Hz, 1H), 5.48 (s, 1H), 3.89-3.81 (m, 2H), 3.08-3.00 (m, 4H), 2.53-2.45 (m, 4H), 2.25 (s, 3H), 2.24 (s, 3H), 1.74 (sext, *J* = 7.4 Hz, 2H), 0.97 (t, *J* = 7.4 Hz, 3H); ^{13}C NMR (100 MHz, MeOD) δ 165.48, 162.13, 159.85, 146.19, 142.81, 141.16, 139.57, 138.48, 135.45, 134.97, 133.31, 129.64, 128.61, 128.57, 127.55, 82.92, 55.09, 54.80, 46.95, 45.75, 22.39, 17.74, 11.51; LCMS $C_{28}H_{34}N_8O_3S$ method (B) R_t = 4.676 min, ESI+ m/z = 564.3 (M+H).

2-(2-((2'-Chloro-4-methyl-4'-((4-methylpiperazin-1-yl)sulfonyl)-[1,1'-biphenyl]-3-yl)(propyl)amino)thiazol-4-yl) pyrimidine-4,6-diamine OR0641 (method A, 28%) as a light yellow solid. Rf = 0.50 (DCM-MeOH-NH_4OH, 90:10:1); ^1H NMR (400 MHz, MeOD) δ 7.90 (d, *J* = 1.7 Hz, 1H), 7.77 (dd, *J* = 8.1, 1.7 Hz, 1H), 7.67 (d, *J* = 8.1 Hz, 1H), 7.54 (d, *J* = 7.6 Hz, 1H), 7.46 (dd, *J* = 7.6, 1.8 Hz, 1H), 7.44 (d, *J* = 1.8 Hz, 1H), 7.26 (s, 1H), 5.53 (s, 1H), 3.99 (brs, 2H), 3.13-3.05 (m, 4H), 2.56-2.48 (m, 4H), 2.32 (s, 3H), 2.27 (s, 3H), 1.73 (sext, *J* = 7.4 Hz, 2H), 0.98 (t, *J* = 7.4 Hz, 3H). ^{13}C NMR (100 MHz, MeOD) δ 171.97, 165.58, 161.20, 152.02, 145.23, 144.10, 139.09, 138.78, 137.57, 134.54, 133.48, 133.36, 131.77, 130.59, 130.21, 127.71, 111.10, 82.96, 55.07, 54.72, 46.94, 45.76, 22.26, 17.64, 11.61; LCMS $C_{28}H_{33}ClN_8O_2S_2$ method (B) R_t = 4.434 min, ESI+ m/z = 613.2 (M+H).

2-(2-((4-Methyl-4'-((4-methylpiperazin-1-yl)sulfonyl)-2'-(trifluoromethyl)-[1,1'-biphenyl]-3-yl)(propyl)amino) thiazol-4-yl)pyrimidine-4,6-diamine OR0642 (method A, 31% or method B, 56%) as a light yellow powder. Rf = 0.27 (DCM-MeOH-NH_4OH, 95:5:0.5); ^1H NMR (400 MHz, MeOD) δ 8.11 (d, *J* = 1.5 Hz, 1H), 8.07 (dd, *J* = 8.0, 1.5 Hz, 1H), 7.70 (d, *J* = 8.0 Hz, 1H), 7.52 (d, *J* = 7.9 Hz, 1H), 7.35 (dd, *J* = 7.9, 1.4 Hz, 1H), 7.31 (d, *J* = 1.4 Hz, 1H), 7.26 (s, 1H), 5.53 (s, 1H), 4.00 (brs, 2H), 3.14-3.06 (m, 4H), 2.57-2.49 (m, 4H), 2.33 (s, 3H), 2.27 (s, 3H), 1.70 (sext, *J* = 7.4 Hz, 2H), 0.98 (t, *J* = 7.4 Hz, 3H).^{13}C NMR (100 MHz, MeOD) δ 171.96, 165.63, 161.29, 152.08, 146.18, 143.87, 139.13, 137.11, 134.77, 133.23, 132.28, 131.14, 130.38 (q, J = 31.2 Hz), 130.12, 126.59 (q, J = 5.4 Hz), 124.78 (q, J = 273.9 Hz), 111.07, 82.98, 55.05, 54.64, 46.90, 45.75, 22.20, 17.60, 11.58. ^{19}F NMR (376 MHz, MeOD) δ -58.40. LCMS $C_{29}H_{33}F_3N_8O_2S_2$ method (B) R_t = 4.608 min, ESI+ m/z = 647.2 (M+H).

2-(2-((2'-Fluoro-4-methyl-4'-((4-methylpiperazin-1-yl)sulfonyl)-[1,1'-biphenyl]-3-yl)(propyl)amino)thiazol-4-yl) pyrimidine-4,6-diamine OR0643 (method A, 32%) as a light brown powder. Rf = 0.55 (DCM-MeOH-NH_4OH, 90:10:1); ^1H NMR (400 MHz, MeOD) δ 7.81-7.75 (m, 1H), 7.68-7.52 (m, 5H), 7.26 (s, 1H), 5.53 (s, 1H), 4.00 (brs, 2H), 3.13-3.05 (m, 4H), 2.56-2.48 (m, 4H), 2.32 (s, 3H), 2.27 (s, 3H), 1.73 (sext, *J* = 7.4 Hz, 2H), 0.99 (t, *J* = 7.4 Hz, 3H); ^{13}C NMR (100 MHz, MeOD) δ 171.92, 165.63, 161.29, 159.37, 152.12, 144.59, 139.43, 137.77, 135.04, 133.97, 133.78, 132.79, 131.41, 130.35, 125.29, 117.08, 111.03, 82.98, 55.08, 54.81, 46.94, 45.76, 22.26, 17.67, 11.59; LCMS $C_{28}H_{33}FN_8O_2S_2$ method (B) R_t = 4.322 min, ESI+ m/z = 597.3 (M+H).

2-(2-((2'-Fluoro-6'-methoxy-4-methyl-4'-((4-methylpiperazin-1-yl)sulfonyl)-[1,1'-biphenyl]-3-yl)(propyl)amino) thiazol-4-yl)pyrimidine-4,6-diamine OR0644 (method A, 24%) as a light yellow solid. Rf = 0.55 (DCM-MeOH-NH_4OH, 90:10:1); ^{1}H NMR (400 MHz, MeOD) δ 7.49 (d, *J* = 7.9 Hz, 1H), 7.39 (d, *J* = 7.9 Hz, 1H), 7.35 (s, 1H), 7.28-7.20 (m, 3H), 5.53 (s, 1H), 3.99 (brs, 2H), 3.88 (s, 3H), 3.15-3.07 (m, 4H), 2.57-2.49 (m, 4H), 2.31 (s, 3H), 2.28 (s, 3H), 1.72 (sext, *J* = 7.4 Hz, 2H), 0.98 (t, *J* = 7.4 Hz, 3H); ^{13}C NMR (100 MHz, MeOD) δ 172.13, 165.61, 162.39, 161.30, 159.88, 152.01, 143.84, 138.53, 137.89, 133.05, 132.91, 131.87, 130.92, 123.26, 111.03, 108.99, 107.48, 82.96, 57.18, 55.11, 54.57, 46.98, 45.77, 22.18, 17.62, 11.61; LCMS $C_{29}H_{35}FN_8O_3S_2$ method (B) R_t = 4.388 min, ESI+ m/z = 627.3 (M+H).

2-(2-((2'-Methoxy-4-methyl-4'-((4-methylpiperazin-1-yl)sulfonyl)-[1,1'-biphenyl]-3-yl)(propyl)amino)thiazol-4-yl) pyrimidine-4,6-diamine OR0645 (method A, 28%) as a light brown solid. Rf = 0.68 (DCM-MeOH-NH_4OH, 90:10:1); ^{1}H NMR (400 MHz, MeOD) δ 7.57 (d, *J* = 7.9 Hz, 1H), 7.52 (dd, *J* = 7.9, 1.7 Hz, 1H), 7.51 (d, *J* = 1.7 Hz, 1H), 7.47 (d, *J* = 7.9 Hz, 1H), 7.44 (dd, *J* = 7.9, 1.7 Hz, 1H), 7.37 (d, *J* = 1.7 Hz, 1H), 7.25 (s, 1H), 5.54 (s, 1H), 4.02 (brs, 2H), 3.89 (s, 3H), 3.12-3.04 (m, 4H), 2.56-2.50 (m, 4H), 2.29 (s, 3H), 2.26 (s, 3H), 1.73 (sext, *J* = 7.6 Hz, 2H), 0.99 (t, *J* = 7.4 Hz, 3H); ^{13}C NMR (100 MHz, MeOD) δ 172.16, 165.50, 161.08, 158.24, 151.83, 143.86, 137.99, 137.83, 136.89, 135.50, 133.12, 132.12, 131.97, 130.75, 121.54, 111.54, 111.08, 82.90, 56.59, 55.11, 54.63, 46.98, 45.76, 22.21, 17.57, 11.63; LCMS $C_{29}H_{36}FN_8O_3S_2$ method (B) R_t = 4.359 min, ESI+ m/z = 609.3 (M+H).

2-(2-((4-Methyl-4'-(piperidin-4-ylsulfonyl)-[1,1'-biphenyl]-3-yl)(propyl)amino)thiazol-4-yl)pyrimidine-4,6-diamine OR0646 (method A, 28%) as a light yellow solid. Rf = 0.20 (DCM-MeOH-NH_4OH, 90:10:1); ^{1}H NMR (400 MHz, MeOD) δ 7.95 (d, *J* = 8.6 Hz, 2H), 7.91 (d, *J* = 8.6 Hz, 2H), 7.71 (dd, *J* = 8.0, 1.9 Hz, 1H), 7.66 (d, *J* = 1.9 Hz, 1H), 7.55 (d, *J* = 8.0 Hz, 1H), 7.25 (s, 1H), 5.54 (s, 1H), 4.01 (brs, 2H), 3.32-3.24 (m, 1H), 3.12-3.04 (m, 2H), 2.59-2.51 (m, 2H), 2.31 (s, 3H), 1.99-1.91 (m, 2H), 1.72 (sext, *J* = 7.4 Hz, 2H), 1.64-1.53 (m, 2H), 0.99 (t, *J* = 7.4 Hz, 3H); ^{13}C NMR (100 MHz, MeOD) δ 171.95, 165.63, 161.27, 152.11, 146.75, 145.05, 140.20, 139.23, 136.81, 134.17, 130.94, 129.54, 128.66, 128.59, 111.00, 82.99, 62.42, 54.89, 45.52, 26.62, 22.30, 17.63, 11.61; LCMS $C_{28}H_{33}N_7O_2S_2$ method (B) R_t = 4.266 min, ESI+ m/z = 564.2 (M+H).

2-(2-((2',6'-Difluoro-4-methyl-4'-((4-methylpiperazin-1-yl)sulfonyl)-[1,1'-biphenyl]-3-yl)(propyl)amino)thiazol-4-yl)pyrimidine-4,6-diamine OR0647 (method A, 27%) as a light yellow solid. Rf = 0.70 (DCM-MeOH-NH_4OH, 90:10:1); ^{1}H NMR (400 MHz, MeOD) δ 7.57 (d, *J* =

8.0 Hz, 1H), 7.54-7.48 (m, 3H), 7.48 (s, 1H), 7.26 (s, 1H), 5.53 (s, 1H), 3.99 (brs, 2H), 3.16-3.08 (m, 4H), 2.57-2.49 (m, 4H), 2.33 (s, 3H), 2.28 (s, 3H), 1.72 (sext, *J* = 7.4 Hz, 2H), 0.98 (t, *J* = 7.4 Hz, 3H); ^{13}C NMR (100 MHz, MeOD) δ 171.90, 165.62, 162.49, 161.27, 159.90, 152.09, 144.38, 139.94, 138.60, 133.52, 132.70, 131.55, 128.50, 112.64, 111.10, 82.98, 55.07, 54.73, 46.92, 45.77, 22.21, 17.72, 11.58; LCMS $C_{28}H_{32}N_8O_2S_2$ method (B) R_t = 4.396 min, ESI+ m/z = 615.2 (M+H).

2-(2-((4'-((4-Aminopiperidin-1-yl)sulfonyl)-4-methyl-[1,1'-biphenyl]-3-yl)(propyl)amino)thiazol-4-yl)pyrimidine-4,6-diamine OR0648 (method C, 90%) as a light yellow solid. Rf = 0.18 (DCM-MeOH-NH_4OH, 90:9:1). ^{1}H NMR (400 MHz, MeOD) δ 7.84 (d, *J* = 8.6 Hz, 2H), 7.81 (d, *J* = 8.6 Hz, 2H), 7.67 (dd, *J* = 8.0, 1.5 Hz, 1H), 7.62 (d, *J* = 1.5 Hz, 1H), 7.51 (d, *J* = 8.0 Hz, 1H), 7.24 (s, 1H), 5.54 (s, 1H), 3.99 (brs, 2H), 3.74-3.66 (m, 2H), 2.60-2.52 (m, 1H), 2.43-2.35 (m, 2H), 2.28 (s, 3H), 1.89-1.81 (m, 2H), 1.71 (sext, *J* = 7.4 Hz, 2H), 1.46-1.34 (m, 2H), 0.96 (t, *J* = 7.4 Hz, 3H); ^{13}C NMR (100 MHz, MeOD) δ 171.92, 165.62, 161.29, 152.14, 145.55, 144.99, 140.27, 138.95, 136.32, 134.13, 129.50, 129.38, 128.54, 128.48, 111.01, 83.03, 54.86, 48.72, 46.43, 34.80, 22.29, 17.64, 11.63; LCMS $C_{28}H_{34}N_8O_2S_2$ method (B) R_t = 4.317 min, ESI+ m/z = 579.3 (M+H).

2-(4-((3'-((4-(4,6-Diaminopyrimidin-2-yl)thiazol-2-yl)(propyl)amino)-4'-methyl-[1,1'-biphenyl]-4-yl)sulfonyl) piperazin-1-yl)acetic acid OR0649 (method A, 20%) as a white solid. Rf = 0.20 (DCM-MeOH-NH_4OH, 80:18:2); ^{1}H NMR (400 MHz, MeOD) δ 7.89 (d, *J* = 8.7 Hz, 2H), 7.85 (d, *J* = 8.7 Hz, 2H), 7.74 (dd, *J* = 8.0, 1.9 Hz, 1H), 7.66 (d, *J* = 1.9 Hz, 1H), 7.57 (s, 1H), 7.56 (d, *J* = 8.0 Hz, 1H), 5.61 (s, 1H), 4.01 (brs, 2H), 3.17 (s, 2H), 3.16-3.12 (m, 4H), 2.86-2.80 (m, 4H), 2.31 (s, 3H), 1.74 (sext, *J* = 7.4 Hz, 2H), 1.01 (t, *J* = 7.4 Hz, 3H); ^{13}C NMR (100 MHz, MeOD) δ 174.37, 172.57, 162.36, 154.84, 146.30, 145.71, 144.52, 140.49, 138.81, 135.64, 134.31, 129.76, 129.26, 128.85, 128.64, 114.62, 81.17, 61.32, 54.98, 53.18, 46.41, 22.21, 17.56, 11.73; LCMS $C_{29}H_{34}N_8O_4S_2$ method (B) R_t = 4.505 min, ESI+ m/z = 623.3 (M+H).

2-(2-((4-Methyl-4'-((4-methylpiperazin-1-yl)sulfonyl)-3'-(trifluoromethoxy)-[1,1'-biphenyl]-3-yl)(propyl)amino) thiazol-4-yl)pyrimidine-4,6-diamine OR0650 (method A, 40%) as a light brown solid. Rf = 0.20 (DCM-MeOH-NH_4OH, 95:5:0.5); ^{1}H NMR (400 MHz, MeOD) δ 8.04 (d, *J* = 8.3 Hz, 1H), 7.81 (dd, *J* = 8.3, 1.7 Hz, 1H), 7.73-7.72 (m, 1H), 7.69 (dd, *J* = 7.9, 2.0 Hz, 1H), 7.66 (d, *J* = 2.0 Hz, 1H), 7.56 (d, *J* = 7.9 Hz, 1H), 7.26 (s, 1H), 5.54 (s, 1H), 4.01 (brs, 2H), 3.27-3.19 (m, 4H), 2.53-2.45 (m, 4H), 2.31 (s, 3H), 2.28 (s, 3H), 1.73 (sext, *J* = 7.4 Hz, 2H), 0.99 (t, *J* = 7.4 Hz, 3H); ^{13}C NMR (100 MHz, MeOD) δ 171.84, 165.58, 161.18, 152.07, 148.09, 147.72, 145.17,

139.95, 138.92, 134.35, 133.68, 130.03, 129.48, 128.48, 126.65, 122.98, 120.66, 111.09, 82.97, 55.37, 54.97, 46.59, 45.86, 22.29, 17.70, 11.60; LCMS $C_{29}H_{33}F_3N_8O_3S_2$ method (B) R_t = 4.530 min, ESI+ m/z = 663.2 (M+H).

***N*-(2-Aminoethyl)-3'-((4-(4,6-diaminopyrimidin-2-yl)thiazol-2-yl)(propyl)amino)-4'-methyl-[1,1'-biphenyl]-4-sulfonamide OR0651** (method C, 100%) as a light yellow solid. Rf = 0.22 (DCM-MeOH-NH_4OH, 90:9:1); ^{1}H NMR (400 MHz, MeOD) δ 7.97 (d, *J* = 8.5 Hz, 2H), 7.87 (d, *J* = 8.5 Hz, 2H), 7.75 (dd, *J* = 8.0, 1.7 Hz, 1H), 7.69 (s, 1H), 7.66 (d, *J* = 1.7 Hz, 1H), 7.58 (d, *J* = 8.0 Hz, 1H), 5.65 (s, 1H), 4.22 (brs, 1H), 3.84 (brs, 1H), 3.18-3.12 (m, 2H), 3.12-3.04 (m, 2H), 2.32 (s, 3H), 1.75 (sext, *J* = 7.4 Hz, 2H), 1.04 (t, *J* = 7.4 Hz, 3H); ^{13}C NMR (100 MHz, MeOD) δ 172.81, 152.43, 145.44, 144.32, 144.07, 140.64, 139.90, 138.72, 134.38, 129.13, 129.01, 128.94, 128.69, 115.92, 80.43, 54.92, 41.39, 40.67, 22.17, 17.50, 11.74. 1; LCMS $C_{26}H_{34}F_3N_8O_2S_2$ method (B) R_t = 4.102 min, ESI+ m/z = 539.3 (M+H).

2-(2-((2-Methyl-5-(4-methyl-6-((4-methylpiperazin-1-yl)sulfonyl)pyridin-3-yl)phenyl)(propyl)amino)thiazol-4-yl) pyrimidine-4,6-diamine OR0652 (method B, 31%) as a light yellow powder. Rf = 0.23 (DCM-MeOH, 90:10); ^{1}H NMR (400 MHz, $CDCl_3$) δ 8.51 (s, 1H), 7.82 (s, 1H), 7.48 (d, *J* = 7.9 Hz, 1H), 7.42 (s, 1H), 7.28 (dd, *J* = 7.9, 1.4 Hz, 1H), 7.20 (d, *J* = 1.4 Hz, 1H), 5.95 (brs, 4H), 5.69 (s, 1H), 4.04 (brs, 2H), 3.40-3.36 (m, 4H), 2.54-2.50 (m, 4H), 2.38 (s, 3H), 2.31 (s, 3H), 2.30 (s, 3H), 1.67 (sext, *J* = 7.4 Hz, 2H), 0.97 (t, *J* = 7.4 Hz, 3H); ^{13}C NMR (100 MHz, $CDCl_3$) δ 170.90, 161.56, 158.18, 154.50, 150.25, 147.16, 142.97, 140.78, 139.57, 137.91, 136.05, 132.93, 130.55, 129.41, 124.70, 112.64, 82.10, 54.50, 53.80, 46.57, 45.93, 21.40, 20.43, 17.62, 11.50; LCMS $C_{28}H_{35}N_9O_2S_2$ method (B) R_t = 4.434 min, ESI+ m/z = 594.2 (M+H).

***N*-(4-Methyl-4'-((4-methylpiperazin-1-yl)sulfonyl)-[1,1'-biphenyl]-3-yl)-*N*-propyl-4-(pyridin-3-yl)thiazol-2-amine OR0659**, a negative control compound closely related to OR0642, was prepared from commercially available 5-bromo-2-methylaniline. Condensation with benzoyl isothiocyanate and saponification gave the corresponding thiourea. Commercially available 3-bromoacetylpyridine was engaged in a Hantzsch thiazole synthesis with the thiourea leading to the corresponding thiazole. The thiazole derivative was then turned into expected *N*-(4-methyl-4'-((4-methylpiperazin-1-yl)sulfonyl)-[1,1'-biphenyl]-3-yl)-*N*-propyl-4-(pyridin-3-yl)thiazol-2-amine OR0659, by Suzuki cross-coupling reaction with commercially available 1-methyl-4-((4-(4,4,5,5-tetramethyl-1,3,2-dioxaborolan-2-yl)phenyl)sulfonyl)piperazine.

***N*-(4-Methyl-4'-((4-methylpiperazin-1-yl)sulfonyl)-[1,1'-biphenyl]-3-yl)-N-propyl-4-(pyridin-3-yl)thiazol-2-amine OR0659** (method B, 29%) as a light brown solid. Rf = 0.29 (DCM-MeOH, 90:10); ^{1}H NMR (400 MHz, $CDCl_3$) ^{1}H NMR (400 MHz, $CDCl_3$) δ 9.09 (d, *J* = 1.9 Hz, 1H), 8.50 (dd, *J* = 4.9, 1.9 Hz, 1H), 8.13 (td, *J* = 7.9, 1.9 Hz, 1H), 7.80 (d, *J* = 8.5 Hz, 2H), 7.71 (d, *J* = 8.5 Hz, 2H), 7.55 (dd, *J* = 8.0, 2.0 Hz, 1H), 7.50 (d, *J* = 2.0 Hz, 1H), 7.47 (d, *J* = 8.0 Hz, 1H), 7.30 (dd, *J* = 7.9, 4.9 Hz, 1H), 6.72 (s, 1H), 3.93 (brs, 2H), 3.09-3.03 (m, 4H), 2.53-2.47 (m, 4H), 2.33 (s, 3H), 2.27 (s, 3H), 1.81 (sext, *J* = 7.4 Hz, 2H), 1.01 (t, *J* = 7.4 Hz, 3H); ^{13}C NMR (100 MHz, $CDCl_3$) δ 170.64, 148.83, 148.48, 147.70, 144.65, 144.06, 139.04, 137.68, 134.07, 133.34, 132.95, 131.06, 128.55, 128.43, 127.54, 127.28, 123.51, 102.87, 54.40, 54.10, 46.01, 45.74, 21.31, 17.65, 11.61; LCMS $C_{29}H_{33}N_5O_2S_2$ method (B) R_t = 5.191 min, ESI+ m/z = 548.2 (M+H).

General Procedure for the Synthesis of 2-[2-(biaryl-3-ylamino)-thiazol-4-yl]-pyrimidine-4,6-diamine hydrochloride salts. To a solution of appropriate 2-[2-(biaryl-3-ylamino)-thiazol-4-yl]-pyrimidine-4,6-diamine (1.5 mmol) in a mixture of methanol-dichloromethane (5:2, 35 mL) was added dropwise a 1N hydrogen chloride solution in diethyl ether (3.2 mL, 3.2 mmol). The resulting solution was stirred at room temperature for 30 minutes and concentrated under reduced pressure until few volumes. The residue was isolated from Et_2O, under vigorous stirring, to afford expected 2-[2-(biaryl-3-ylamino)-thiazol-4-yl]-pyrimidine-4,6-diamine hydrochloride salt.

2-(2-((4-Methyl-4'-((4-methylpiperazin-1-yl)sulfonyl)-[1,1'-biphenyl]-3-yl)(propyl)amino)thiazol-4-yl)pyrimidine-4,6-diamine hydrochloride salt OR0624 (97%) as a light yellow powder. ^{1}H NMR (400 MHz, MeOD) δ 7.92 (d, *J* = 8.7 Hz, 2H), 7.89 (d, *J* = 8.7 Hz, 2H), 7.76 (dd, *J* = 8.0, 1.6 Hz, 1H), 7.68 (s, 1H), 7.67 (d, *J* = 1.6 Hz, 1H), 7.59 (d, *J* = 8.0 Hz, 1H), 5.66 (s, 1H), 4.27 (brs, 1H), 3.89 (brs, 1H), 3.30-3.236 (m, 4H), 3.10-3.06 (m, 4H), 2.66 (s, 3H), 2.32 (s, 3H), 1.76 (sext, *J* = 7.4 Hz, 2H), 1.04 (t, *J* = 7.4 Hz, 3H). ^{13}C NMR (100 MHz, MeOD) δ 172.76, 152.50, 145.92, 144.37, 144.16, 140.43, 138.88, 135.53, 134.41, 129.76, 129.20, 129.01, 128.81, 115.88, 80.48, 54.97, 54.43, 45.56, 44.37, 22.20, 17.52, 11.78. LCMS $C_{28}H_{34}N_8O_2S_2$ method (B) R_t = 4.351 min, ESI+ m/z = 579.3 (M+H).

2-(2-((2-Methyl-5-(6-((4-methylpiperazin-1-yl)sulfonyl)pyridin-3-yl)phenyl)(propyl)amino)thiazol-4-yl)pyrimidine-4,6-diamine hydrochloride salt OR0634 (91%) as a light yellow powder. ^{1}H NMR (400 MHz, MeOD) δ 9.06 (d, *J* = 2.1 Hz, 1H), 8.38 (dd, *J* = 8.2, 2.1 Hz, 1H), 8.10 (d, *J* = 8.2 Hz, 1H), 7.84 (dd, *J* = 8.0, 1.8 Hz, 1H), 7.79 (d, *J* = 1.8 Hz, 1H), 7.72 (s, 1H), 7.67 (d, *J* = 8.0 Hz, 1H), 5.67 (s, 1H), 4.25 (brs, 1H), 4.16-4.08 (m, 2H), 3.93 (brs,

1H), 3.65-3.58 (m, 2H), 3.38-3.34 (m, 2H), 3.28-3.22 (m, 2H), 2.96 (s, 3H), 2.36 (s, 3H), 1.79 (sext, *J* = 7.4 Hz, 2H), 1.06 (t, *J* = 7.4 Hz, 3H). ^{13}C NMR (100 MHz, MeOD) δ 172.66, 155.69, 152.35, 149.57, 144.61, 144.03, 140.17, 139.77, 137.71, 137.33, 134.69, 129.43, 129.19, 124.49, 115.98, 80.47, 55.09, 54.28, 45.11, 43.60, 22.18, 17.60, 11.77. LCMS $C_{27}H_{33}N_9O_2S_2$ method (A) R_t = 4.152 min, ESI+ m/z = 580.3 (M+H).

2-(2-((4-Methyl-4'-((4-methylpiperazin-1-yl)sulfonyl)-2'-(trifluoromethyl)-[1,1'-biphenyl]-3-yl)(propyl)amino) thiazol-4-yl)pyrimidine-4,6-diamine hydrochloride salt OR0642-HCl (93%) as a light yellow powder. ^{1}H NMR (400 MHz, MeOD) δ 8.17 (s, 1H), 8.15 (dd, *J* = 8.1, 1.7 Hz, 1H), 7.74 (d, *J* = 8.0 Hz, 1H), 7.69 (s, 1H), 7.57 (d, *J* = 8.0 Hz, 1H), 7.41 (dd, *J* = 7.9, 1.6 Hz, 1H), 7.34 (s, 1H), 5.67 (s, 1H), 4.28 (brs, 1H), 4.07-3.97 (m, 2H), 3.85 (brs, 1H), 3.67-3.57 (m, 2H), 3.33-3.27 (m, 2H), 2.99-2.87 (m, 2H), 2.92 (s, 3H), 2.34 (s, 3H), 1.72 (sext, *J* = 7.4 Hz, 2H), 1.02 (t, *J* = 7.4 Hz, 3H). ^{13}C NMR (100 MHz, MeOD) δ 172.73, 152.34, 146.43, 144.00, 143.15, 139.26, 138.98, 136.95, 135.12, 133.51, 132.38, 130.94, 130.62 (q, *J* = 31.0 Hz), 126.71 (q, *J* = 5.4 Hz), 124.68 (q, *J* = 274.0 Hz), 115.99, 80.46, 54.70, 53.95, 44.53, 43.49, 22.09, 17.51, 11.75. ^{19}F NMR (376 MHz, MeOD) δ -58.33. LCMS $C_{29}H_{33}F_3N_8O_2S_2$ method (B) R_t = 4.742 min, ESI+ m/z = 647.2 (M+H).

(*R*)-2-((1-(2-(4-methoxy-3-(2-morpholinoethoxy)phenyl)-5-methylthiazol-4-yl)ethyl)thio)pyrimidine-4,6-diamine *(R)*-DI-87, a positive control compound, was prepared following a modified procedure from literature (see. S. Poddar et al., Biochemical Pharmacology 172 (2020) 113742, DOI: 10.1016/j.bcp.2019.113742). Instead of using an enantioselective synthesis albeit with a low yield, DI-87 was synthesized as a racemic compound, followed by separation of the enantiomers. The enantiomers of DI-87 were separated by chiral HPLC using the following conditions: Chiralpak IA (250 x 10 mm), hexane / ethanol with 0.1% of triethylamine / dichloromethane (50/30/20) as mobile phase, flow-rate = 5 mL/min, UV detection at 254 nm. Each enantiomer of DI-87 was obtained with ee>99%. Spectroscopic data were in agreement with those reported in the literature.

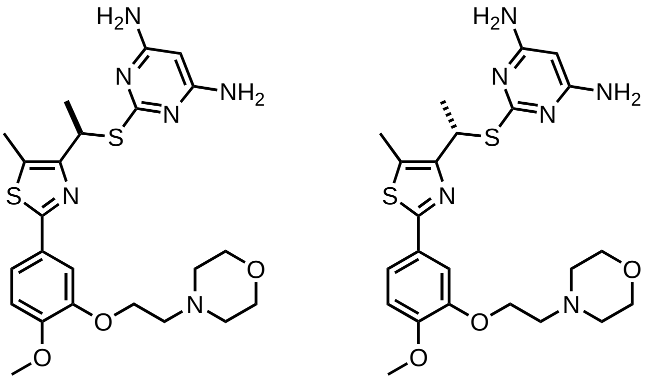

***(R)*-DI-87** ***(S)*-DI-87**

Supplementary Figure 24. Chemical structures of DI-87 enantiomers; (*R*)-DI-87 and (*S*)-DI-87.

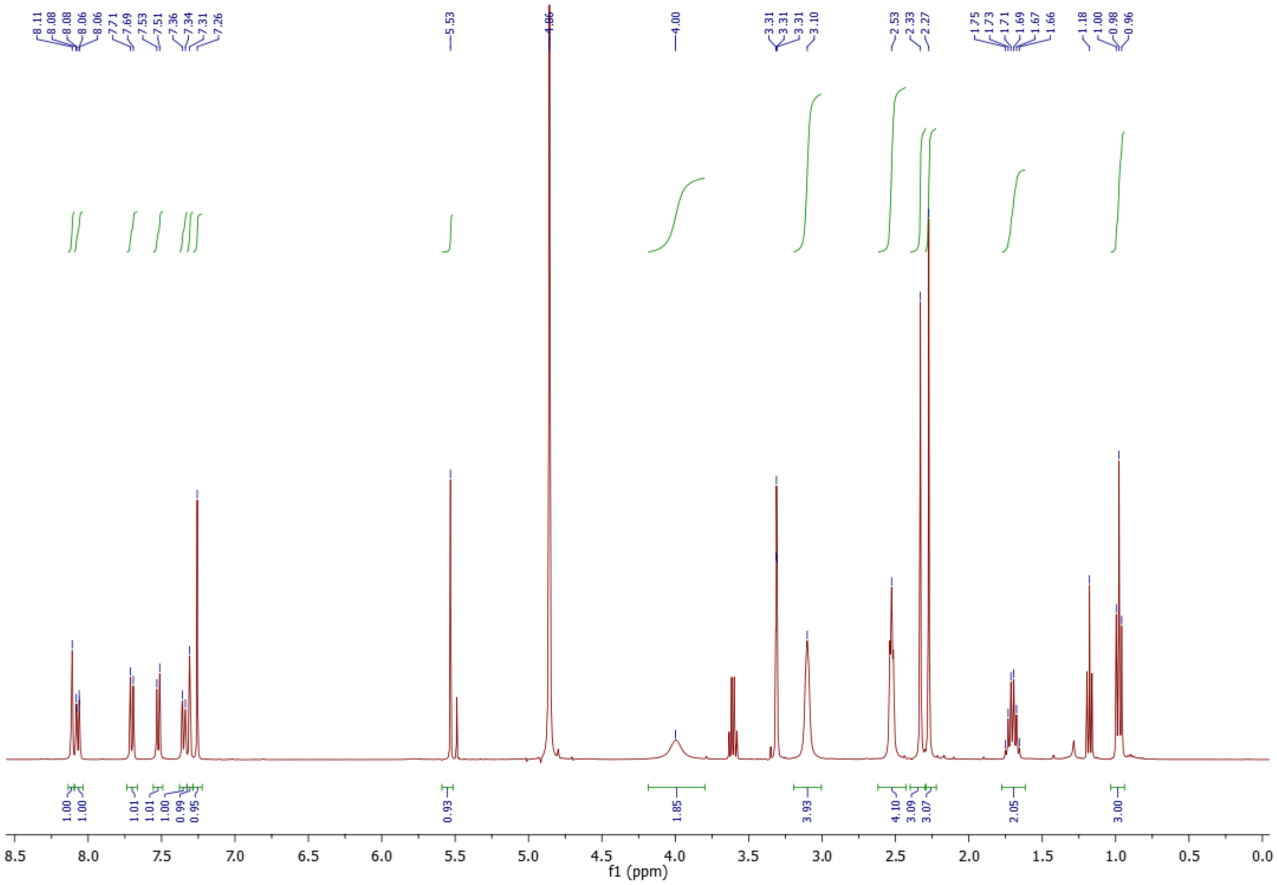

Supplementary Figure 25. ^{1}H NMR (400 MHz, MeOD) spectrum of OR0642.

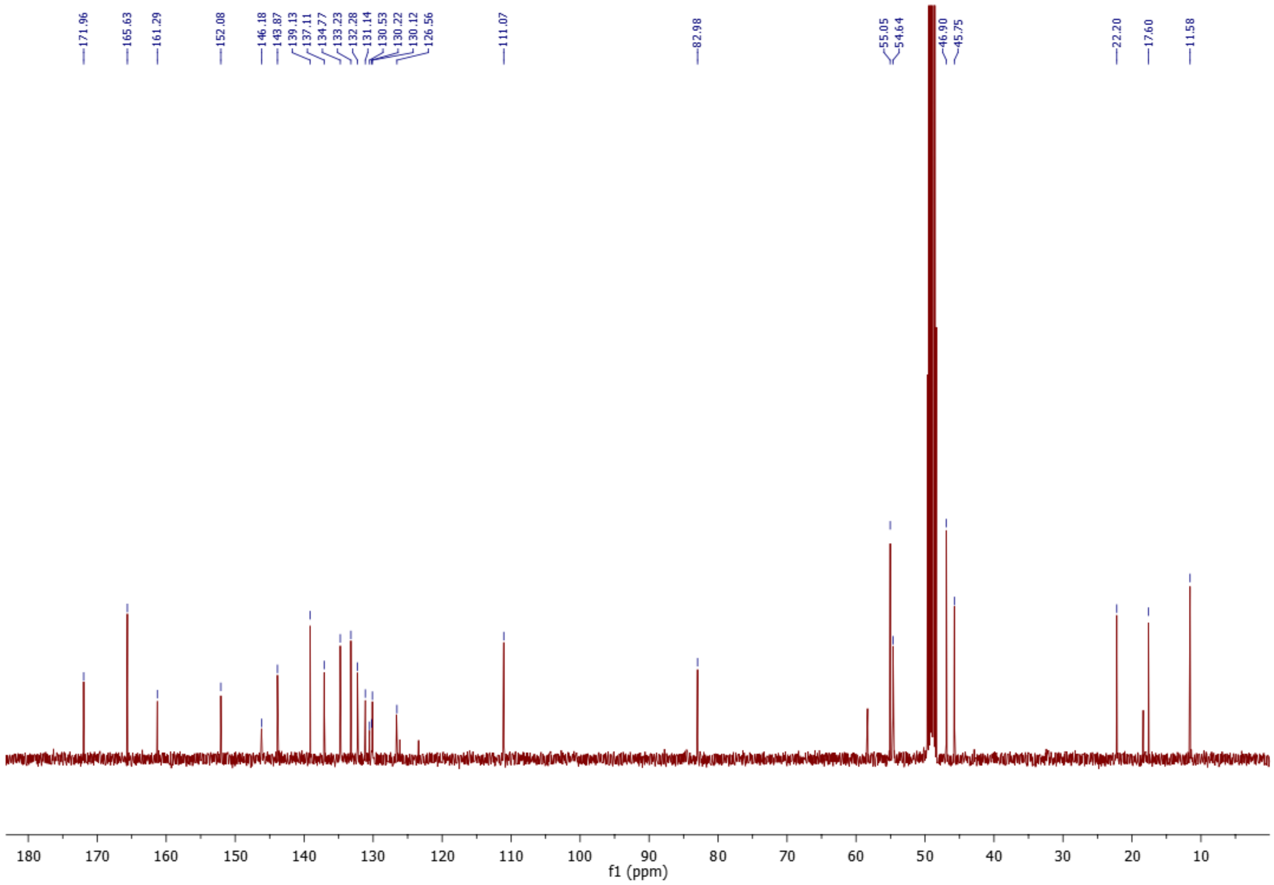

Supplementary Figure 26. ^{13}C NMR (100 MHz, MeOD) spectrum of OR0642.

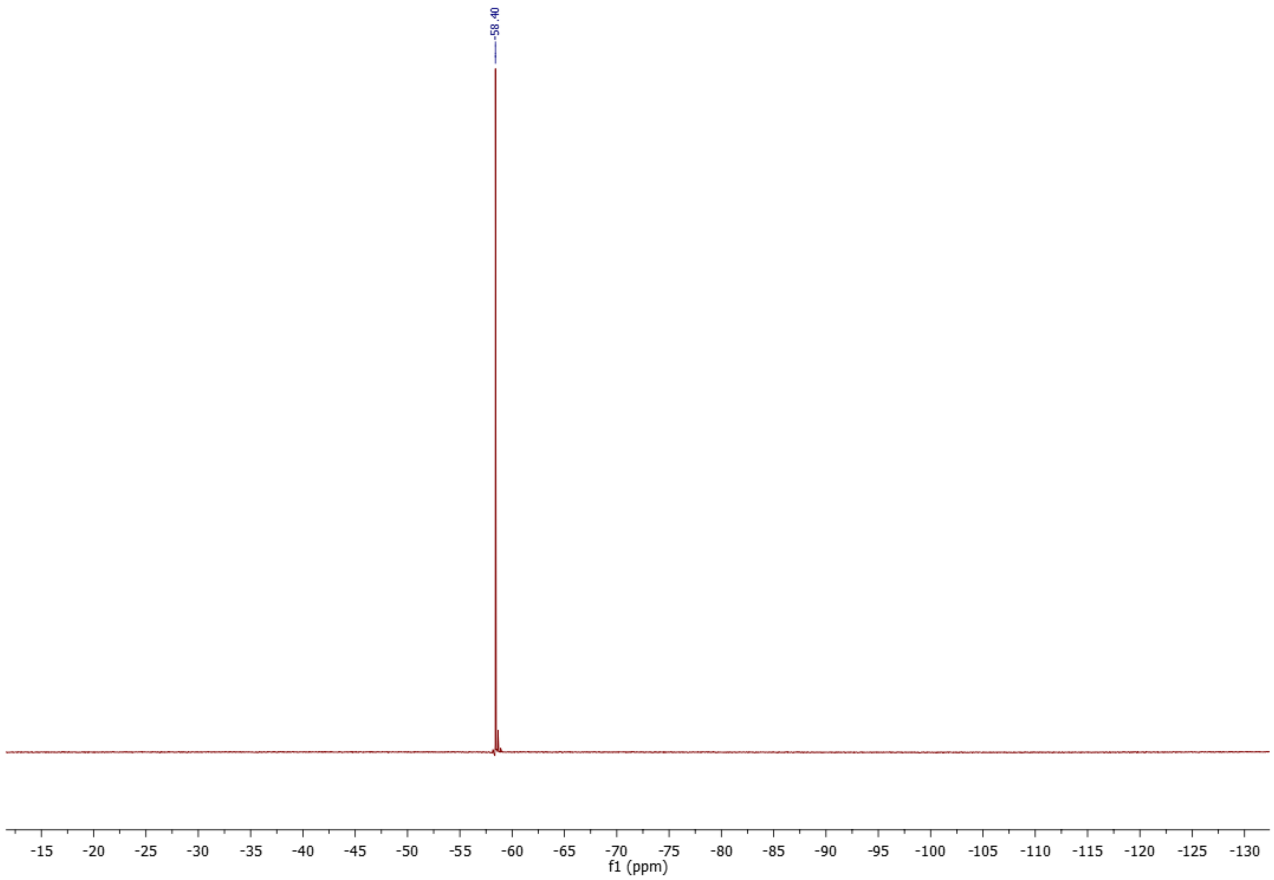

Supplementary Figure 27. ^{19}F NMR (376 MHz, MeOD) spectrum of OR0642.

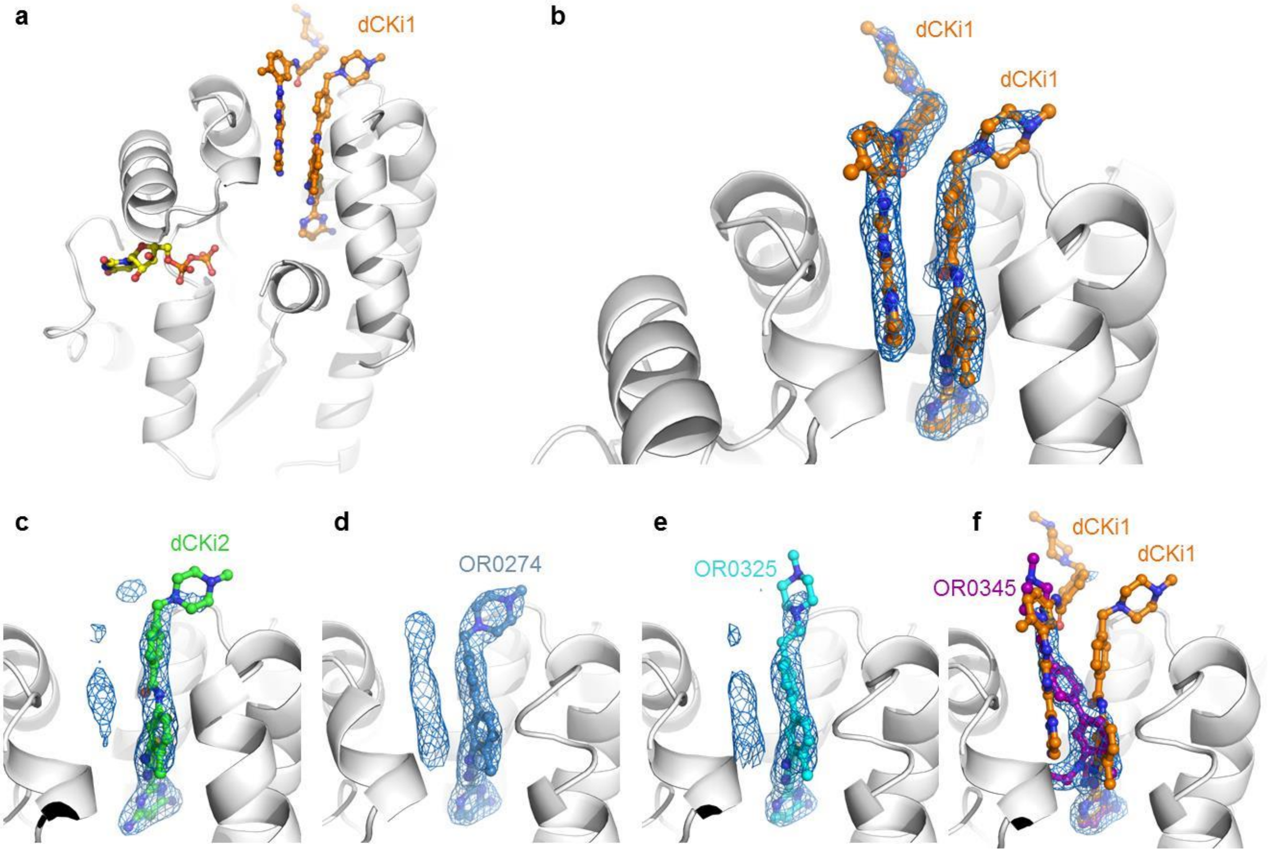

Supplementary Figure 28. Residual electron densities in dCKi1, dCKi2, OR0274, and OR0325 structures. a Structure of dCK in complex with dCKi1 showing the two ligands per protein. **b** Detail of the electronic density of the two dCKi1 ligands. **c-e** A residual electron density parallel to the C-D rings was also observed for several compounds. Electron densities for compounds dCKi2 (**c**), OR0274 (**d**), and OR0325 (**e**) are showed. This indicates a potential low affinity second binding site confirmed by the complex ITC thermograms observed with dCKi1 and dCKi2 (Supplemental Figure 2). **f** When comparing dCKi1 (orange) to OR0345 (purple), the addition of a N-propyl moiety on OR0345 filled the hydrophobic cavity, shifted the compound and prevented the binding of the second molecule.

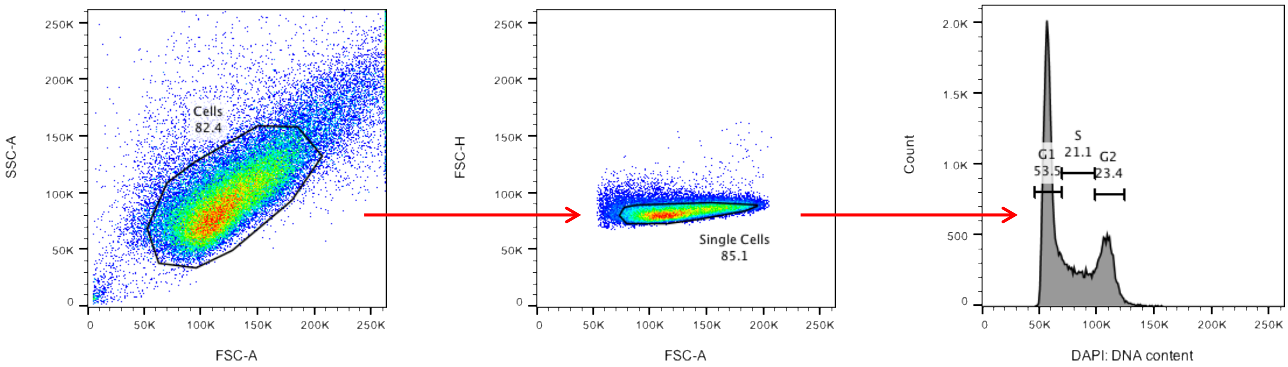

Supplementary Figure 29. Flow cytometry gating strategy for cell cycle analysis. Example of the gating hierarchy of a representative sample (DMSO) in the CCRF-CEM cell line.

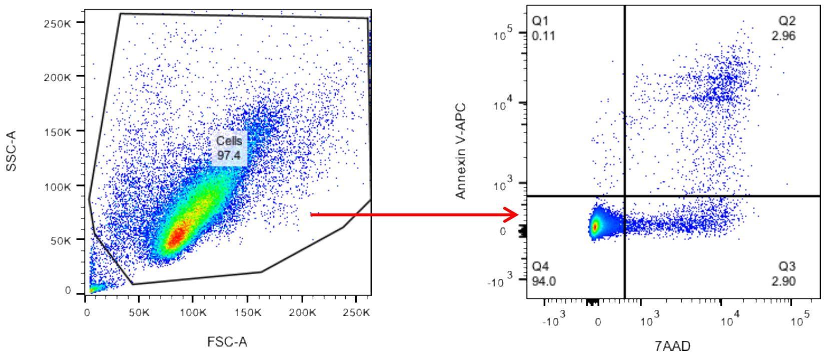

Supplementary Figure 30. Flow cytometry gating strategy for apoptosis assay. Example of the gating hierarchy of a representative sample (DMSO) in the CCRF-CEM cell line.

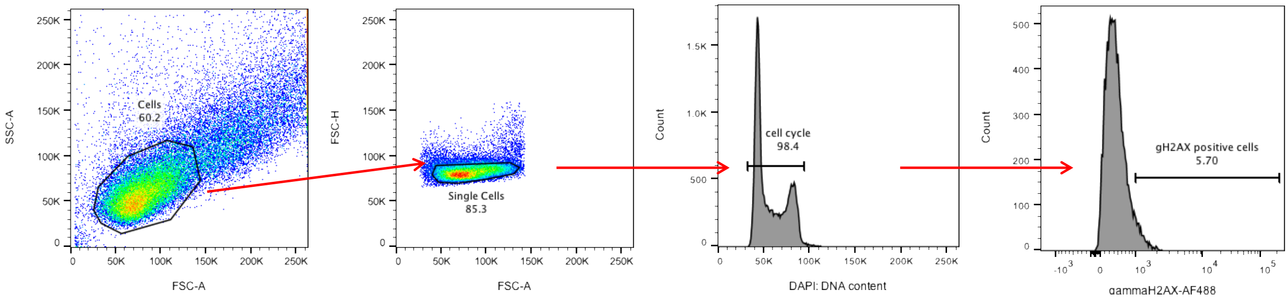

Supplementary Figure 31. Flow cytometry gating strategy for DNA damage assay (ɣH2AX). Example of the gating hierarchy of a representative sample (DMSO) in the CCRF-CEM cell line.

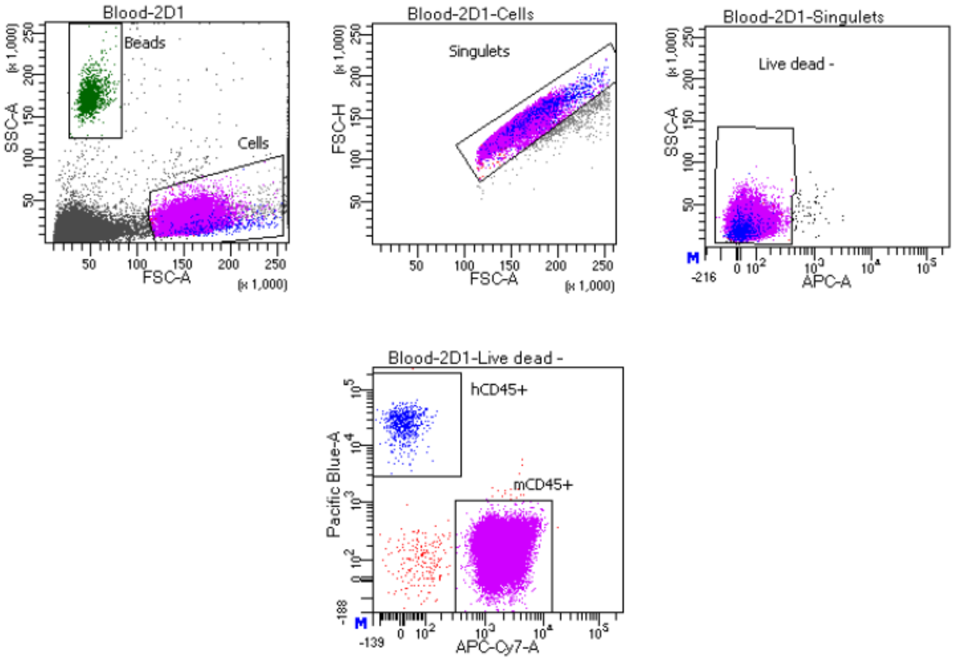

Supplementary Figure 32. Flow cytometry gating strategy in the CDX model. Example of the gating strategy used to determine the fraction of human blasts (Live Dead-/hCD45+/mCD45- cells) in the CCRF-CEM mouse model using flow cytometry.

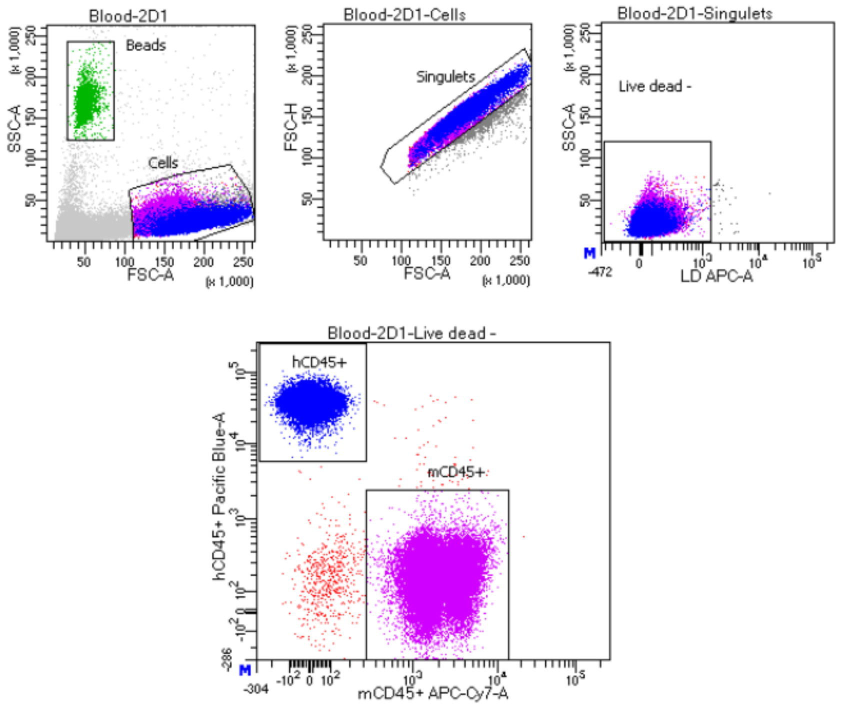

Supplementary Figure 33. Flow cytometry gating strategy in the PDX model. Example of the gating strategy used to determine the fraction of human blasts (Live Dead-/hCD45+/mCD45- cells) in the PDX mouse model UPNT525 using flow cytometry.
