# Supplementary material for: From a drug repositioning to a structure-based drug design approach to tackle acute lymphoblastic leukemia
Source: Nat Commun. 2023 May 29;14:3079. doi: 10.1038/s41467-023-38668-2 (PMC10227015; doi:10.1038/s41467-023-38668-2)
Supplement: Supplementary file 2 — Reporting Summary [file 41467_2023_38668_MOESM2_ESM.pdf]

nature portfolio

Corresponding author(s): Magali Saez-Ayala, Patrice Dubreuil, Sébastien Combes & Xavier Morelli

Last updated by author(s): Apr 13, 2023

Reporting Summary

Nature Portfolio wishes to improve the reproducibility of the work that we publish. This form provides structure for consistency and transparency in reporting. For further information on Nature Portfolio policies, see our Editorial Policies and the Editorial Policy Checklist.

Statistics

For all statistical analyses, confirm that the following items are present in the figure legend, table legend, main text, or Methods section.

n/a	Confirmed	
☐	☒	The exact sample size (*n*) for each experimental group/condition, given as a discrete number and unit of measurement
☐	☒	A statement on whether measurements were taken from distinct samples or whether the same sample was measured repeatedly
☐	☒	The statistical test(s) used AND whether they are one- or two-sided<br>*Only common tests should be described solely by name; describe more complex techniques in the Methods section.*
☒	☐	A description of all covariates tested
☒	☐	A description of any assumptions or corrections, such as tests of normality and adjustment for multiple comparisons
☐	☒	A full description of the statistical parameters including central tendency (e.g. means) or other basic estimates (e.g. regression coefficient) AND variation (e.g. standard deviation) or associated estimates of uncertainty (e.g. confidence intervals)
☐	☒	For null hypothesis testing, the test statistic (e.g. *F*, *t*, *r*) with confidence intervals, effect sizes, degrees of freedom and *P* value noted<br>*Give P values as exact values whenever suitable.*
☒	☐	For Bayesian analysis, information on the choice of priors and Markov chain Monte Carlo settings
☒	☐	For hierarchical and complex designs, identification of the appropriate level for tests and full reporting of outcomes
☐	☒	Estimates of effect sizes (e.g. Cohen's *d*, Pearson's *r*), indicating how they were calculated

Our web collection on statistics for biologists contains articles on many of the points above.

Software and code

Policy information about availability of computer code

Data collection	Bio-Rad CFX Manager 3.1 (Thermal shift assay), MicroCal Origin 9.1 (Isothermal titration calorimetry), PHERAstar Control Software 5.70 R5 (enzymatic assays), MXCuBE (MXCuBE3 version) (X-ray collection), CLARIOstar Control Software 5.70 R2 (cellular assays), PhotonIMAGER Optima Software (Bioluminiscence analysis), BD FACSDiva Software 9.0.1 (Flow cytometry)
Data analysis	Chemaxon Standardizer 20.16.0 and SeeSAR Software 9 (in silico DOTS), Bio-Rad CFX Manager 3.1 (Thermal shift assay), GraphPad Prism Software 9.4.0 (enzymatic assays, cellular assays, statistics), MicroCal Origin 9.1 (Isothermal titration calorimetry), Mars Data Analysis Software 4.0 R2 (enzymatic assays), XDS suite (version Jan 10, 2022) (X-ray processing), CCP4i2 version 1.1.0 built on 02/Mar/21 (DIALS/xia2, PHASER, REFMAC5, COOT, PRODRG) (X-ray processing), Molecular Operating Environment Software 2022.02 (Protein-ligand interaction diagrams), Mars Data Analysis Software 3.42 R5 (cellular assays), BD FACSDiva Software 9.0.1 (Flow cytometry), FlowJo Software 10.7.1 (cell cycle analysis, apoptosis assays, γH2A.X measurements, proliferation indexes), GraphPad Prism Software 8.0.2 (statistical analysis for animal studies)

For manuscripts utilizing custom algorithms or software that are central to the research but not yet described in published literature, software must be made available to editors and reviewers. We strongly encourage code deposition in a community repository (e.g. GitHub). See the Nature Portfolio guidelines for submitting code & software for further information.

Data

Policy information about availability of data

All manuscripts must include a data availability statement. This statement should provide the following information, where applicable:

- Accession codes, unique identifiers, or web links for publicly available datasets
- A description of any restrictions on data availability
- For clinical datasets or third party data, please ensure that the statement adheres to our policy

Structural data for dCK in complex with dCKi1, dCKi2, OR0642, OR0274, OR0325, OR0345, OR0602, OR0624, OR0634, and OR0635 have been deposited in the Protein Data Bank (PDB), with the accession codes 7ZI1, 7ZI2, 7ZI3, 7ZI5, 7ZI6, 7ZI7, 7ZI8, 7ZI9, 7ZIA, and 7ZIB, respectively. The starting model for molecular replacement is also available in the PDB (4KCG). Source data are provided with this paper. The other supplementary data that support the findings of this study are available from the corresponding author upon request.

Human research participants

Policy information about studies involving human research participants and Sex and Gender in Research.

Reporting on sex and gender	Sex and gender were not considered in the study design, as PDX samples were randomly selected to perform the proof of concept from those available.
Population characteristics	Age and sex of the patients reflect the clinical manifestation of the disease.
Recruitment	Patients were enrolled in the GRAALL-2003-2005 trials (registered on http://clinicaltrials.gov as follows: GRAALL-2003, #NCT00222027; GRAALL-2005, #NCT00327678).
Ethics oversight	Diagnostic peripheral blood or bone marrow samples were collected after informed consent according to the Declaration of Helsinki.

Note that full information on the approval of the study protocol must also be provided in the manuscript.

Field-specific reporting

Please select the one below that is the best fit for your research. If you are not sure, read the appropriate sections before making your selection.

[x] Life sciences   [ ] Behavioural & social sciences   [ ] Ecological, evolutionary & environmental sciences

For a reference copy of the document with all sections, see nature.com/documents/nr-reporting-summary-flat.pdf

Life sciences study design

All studies must disclose on these points even when the disclosure is negative.

Sample size	The choice of the sample size was based on our previous studies using similar experimental procedures. (De Gassart A, et al. Development of ICT01, a first-in-class, anti-BTN3A antibody for activating Vγ9Vδ2 T cell-mediated antitumor immune response. Sci Transl Med. 13(616) (2021). Andrieu, G. P. et al. PRC2 loss of function confers a targetable vulnerability to BET proteins in T-ALL. Blood 138, 1855-1869 (2021). Touzart, A. et al. Epigenetic analysis of patients with T-ALL identifies poor outcomes and a hypomethylating agent-responsive subgroup. Sci Transl Med 13 (2021)).
Data exclusions	No data were excluded from the analyses.
Replication	Experiments were performed independently several times (as detailed in methods section, usually N=3) and all attempts at replication were successful.
Randomization	Mice and samples were randomly assigned in groups.
Blinding	Blinding was not relevant to our study as no measure was subjective.

Reporting for specific materials, systems and methods

We require information from authors about some types of materials, experimental systems and methods used in many studies. Here, indicate whether each material, system or method listed is relevant to your study. If you are not sure if a list item applies to your research, read the appropriate section before selecting a response.

Materials & experimental systems

n/a	Involved in the study
☐	☒ Antibodies
☐	☒ Eukaryotic cell lines
☒	☐ Palaeontology and archaeology
☐	☒ Animals and other organisms
☒	☐ Clinical data
☒	☐ Dual use research of concern

Methods

n/a	Involved in the study
☒	☐ ChIP-seq
☐	☒ Flow cytometry
☒	☐ MRI-based neuroimaging

Antibodies

Antibodies used	Phospho-H2AX (pS139 H2AX, Biolegend, 613402) specific primary antibody, Alexa-Fluor 488 secondary antibody (ThermoFisher, A21202), Cas9 Antibody (7A9-3A3, Santa Cruz, sc-517386), Pacific blue-conjugated anti-hCD45 (Biolegend, 304029) and APC eFluor780-conjugated anti-mCD45 (ThermoFisher Scientific, 47-0451-82)
Validation	Phospho-H2AX (pS139 H2AX, Biolegend, 613402) specific primary antibody: each lot of this antibody is quality control tested by Western blotting.<br>Alexa-Fluor 488 secondary antibody (ThermoFisher, A21202) is delivered with a certificate of analysis for each product lot.<br>Cas9 Antibody (7A9-3A3, Santa Cruz, sc-517386) has been validated by Western blotting.<br>Pacific blue-conjugated anti-hCD45 (Biolegend, 304029): each lot of this antibody is quality control tested by immunofluorescent staining with flow cytometric analysis.<br>APC eFluor780-conjugated anti-mCD45 (ThermoFisher Scientific, 47-0451-82): this 30-F11 antibody has been tested by flow cytometric analysis of mouse bone marrow cells.

Eukaryotic cell lines

Policy information about cell lines and Sex and Gender in Research

Cell line source(s)	CCFR-CEM cells were obtained from Olivier Hermine laboratory, (Paris, France). The generation of Ba/F3 cells expressing wild-type human c-KIT has been previously described by the team (Casteran, N. et al. Signal transduction by several KIT juxtamembrane domain mutations. Oncogene 22, 4710-4722 (2003)) (Gabillot-Carre, M. et al. Rapamycin inhibits growth and survival of D816V-mutated c-kit mast cells. Blood 108, 1065-1072 (2006)). X63-omIL-3 cells were obtained from Fritz Melchers laboratory (Berlin, Germany). The generation of CRIPR dCK- cell line is described in the Methods section.
Authentication	None of the cell lines used were authenticated.
Mycoplasma contamination	All cell lines were tested negative for mycoplasma (Mycoalert detection kit, Lonza)
Commonly misidentified lines (See ICLAC register)	No commonly misidentified cell lines were used in the study.

Animals and other research organisms

Policy information about studies involving animals; ARRIVE guidelines recommended for reporting animal research, and Sex and Gender in Research

Laboratory animals	7 weeks-old male Swiss mice (pharmacokinetics) and 6 to 9 weeks-old male NOD-SCID-IL2R common-γ-chain-knockout mice (NSG) (animals models). Mice were maintained under pathogen-free conditions on a 12-h light and 12-h dark cycle. Temperature was maintained between 20 and 24°C and the hygrometry between 40 and 60%.
Wild animals	The study did not involve wild animals
Reporting on sex	Sex was not considered in the study design. In vivo protocols were realized in male mice.<br>Except to target sexual organs, the safety and the pharmacokinetic of compounds is not affected by the sex of the mouse. In consequence, to avoid variability induced by uncontrolled menstrual cycle in female, only male mice were used. As the safety of OR0642 and characterization of the kinetics of development of the preclinical mouse models for T-ALL (CDX and PDX xenografts) were performed in male mice, we used male mice in the efficacy study.
Field-collected samples	The study did not involve samples collected from the field.
Ethics oversight	All experiments were performed using standard methods in agreement with the French Guidelines for animal handling and approved by the local ethics committee APAFIS#6743 and APAFIS#3671.

Note that full information on the approval of the study protocol must also be provided in the manuscript.

Flow Cytometry

Plots

Confirm that:

- [x] The axis labels state the marker and fluorochrome used (e.g. CD4-FITC).
- [x] The axis scales are clearly visible. Include numbers along axes only for bottom left plot of group (a 'group' is an analysis of identical markers).
- [x] All plots are contour plots with outliers or pseudocolor plots.
- [x] A numerical value for number of cells or percentage (with statistics) is provided.

Methodology

Sample preparation	For in vivo models peripheral blood was obtained to determine the fraction of human blasts using flow cytometry. Mononuclear cells were labelled with Pacific blue-conjugated anti-hCD45 (Biolegend, 304029), APC eFluor780-conjugated anti-mCD45 (ThermoFisher Scientific, 47-0451-82) and live/dead Fixable Far Red Dead Cell Stain kit (ThermoFisher Scientific, L10120) to determine the fraction of human blasts (Live Dead-/hCD45+/mCD45- cells) using flow cytometry. Analyses were performed on a Life Science Research Fortessa flow cytometer with FACSDiva Software 9.0.1 (BD Biosciences). The number of ALL cells/L peripheral blood was determined by using CountBright beads (Invitrogen, C36950).<br>For ex vivo cytometry, blasts (hCD45+ hCD7+) were collected from the experiments, washed by centrifugation at 4°C for five min at 300 x g, and stained with Annexing V/propidium iodide in ice-cold Annexing Binding Buffer (Biolegend) for 15 min. Cells were then analysed by flow cytometry on a BD LSR Fortessa equipped with a high-throughput plate reader system and FACSDiva Software 9. All the analysed cells were leukemic blasts.<br>For cell cycle analysis, cells were harvested after 24hours, fixed in 4% paraformaldehyde, permeabilized with 0.15% Triton X100. and DNA content was determined using 1µg/ml DAPI.<br>For apoptosis assay, cells were harvested after 72hours, and cell death was assayed using Annexin V-APC and 7AAD following manufacturer's instructions (Biolegend, 640930).<br>For γH2A.X measurements, cells were harvested after 24hours, fixed in 4% paraformaldehyde and permeabilized with 0.15% Triton X100. Then, cells were stained with the phospho-H2AX (pS139 H2AX, Biolegend, 613402) specific primary antibody, Alexa-Fluor 488 secondary antibody (ThermoFisher, A21202) and DAPI.
Instrument	Life Science Research Fortessa flow cytometer (BD Biosciences)
Software	BD FACSDiva Software 9.0.1 (BD Biosciences)<br>FlowJo Software 10.7.1 (cell cycle analysis, apoptosis assays, γH2A.X measurements)
Cell population abundance	In the in vivo models: for the CCRF-CEM control group, the human cells (hCD45) represented 1.6% of the living cells (SD= +/-0.6) and for the UPNT525 control group, the hCD45=32% (SD=+/-13).<br>For ex vivo cytometry all the analysed cells were leukemic blasts.<br>For in vitro assays: at least 10,000 events were acquired for every population of interest.
Gating strategy	For in vivo models human CD45 cells were identified as follows: singlet cells (FSC-H versus FSC-A), live dead negative, murine CD45 negative, human CD45 positive cells.<br>For ex vivo cytometry: FSChi SSC, doublet exclusion on FSC-H vs FSC-A, and annexin V APC/PI (PE equivalent) quadrants. Viable cells were identified as AnnexinV-negative, PI-negative.<br>For in vitro assays: debris were excluded based on FSC-A/SSC-A dot plot, and singlets were gated according to FSC-A/FSC-H dot-plot.

- [x] Tick this box to confirm that a figure exemplifying the gating strategy is provided in the Supplementary Information.
